# Selective C–H Activation of Molecular Nanodiamonds via Photoredox Catalysis

**DOI:** 10.1021/acscatal.4c00296

**Published:** 2024-03-01

**Authors:** Hoang T. Dang, Henry T. O’Callaghan, Mikayla M. Wymore, Jennifer Suarez, David B. C. Martin

**Affiliations:** † Department of Chemistry, University of Iowa, Iowa City, Iowa 52242, United States

**Keywords:** Diamondoids, photoredox catalysis, radical functionalization, C−C bond formation, pyrylium photocatalysis

## Abstract

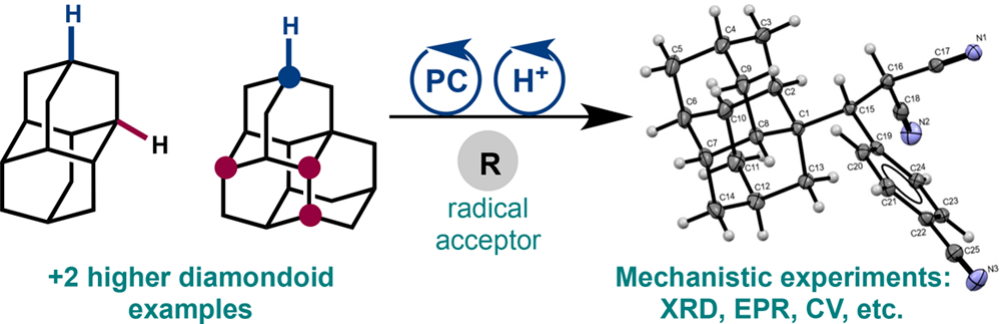

While substituted adamantanes have widespread use in medicinal chemistry, materials science, and ligand design, the use of diamantanes and higher diamondoids is limited to a much smaller number. Selective functionalization beyond adamantane is challenging, as the number of very similar types of C–H bonds (secondary, 2°, and tertiary, 3°) increases rapidly, and H atom transfer does not provide a general solution for site selectivity. We report a method using pyrylium photocatalysts that is effective for nanodiamond functionalization in up to 84% yield with exclusive 3° selectivity and moderate levels of regioselectivity between 3° sites. The proposed mechanism involving photooxidation, deprotonation, and radical C–C bond formation is corroborated through Stern–Volmer luminescence quenching, cyclic voltammetry, and EPR studies. Our photoredox strategy offers a versatile approach for the streamlined synthesis of diamondoid building blocks.

## Introduction

**Figure 1:**
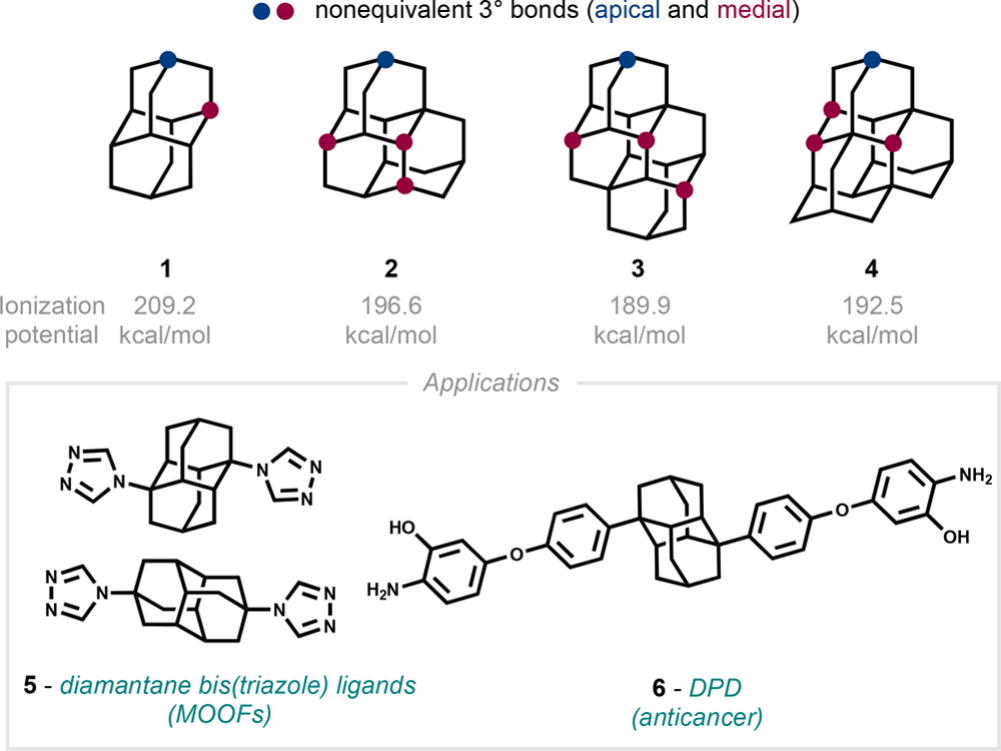
Diamondoid structures and applications.

Molecular nanodiamonds, also known as diamondoids, are polycyclic substructures of the diamond lattice comprising one or more face-fused adamantane units and have shown promising properties with numerous applications in catalysis, materials science, and pharmaceuticals.^1−3^ The second member of the family, diamantane (**1**, Figure 1), has been incorporated into a substantial number of functional compounds in these areas, such as ligand linkers for MOOFs (compound **5**)^4^ and the anticancer compound **6**;^3^ however, further development is hampered by limited availability of functionalized building blocks. In stark contrast to adamantane and even diamantane, higher analogs such as triamantane (**2**), tetramantanes (**3** and **4**), and beyond are virtually unexplored due to challenges in their supply; there are currently no commercial vendors of higher diamondoids, which must be painstakingly isolated from petroleum sources.^5^ Furthermore, while adamantane has been extensively studied as a substrate for C–H functionalization, the increased complexity of diamantane (**1**) and higher diamondoids (**2**–**4**) presents a heightened challenge for achieving regioselectivity, due to increasing numbers of nonequivalent tertiary and secondary C–H bonds. Any methods seeking to enable access to new substituted diamondoids must address their selective activation with new strategies that can distinguish between strong C–H bonds in slightly different environments, without relying on inherent substrate control imparted by deactivating (electron-withdrawing) groups or activating (α-heteroatom or alkene/arene) groups.^6,7^

**Scheme 1:**
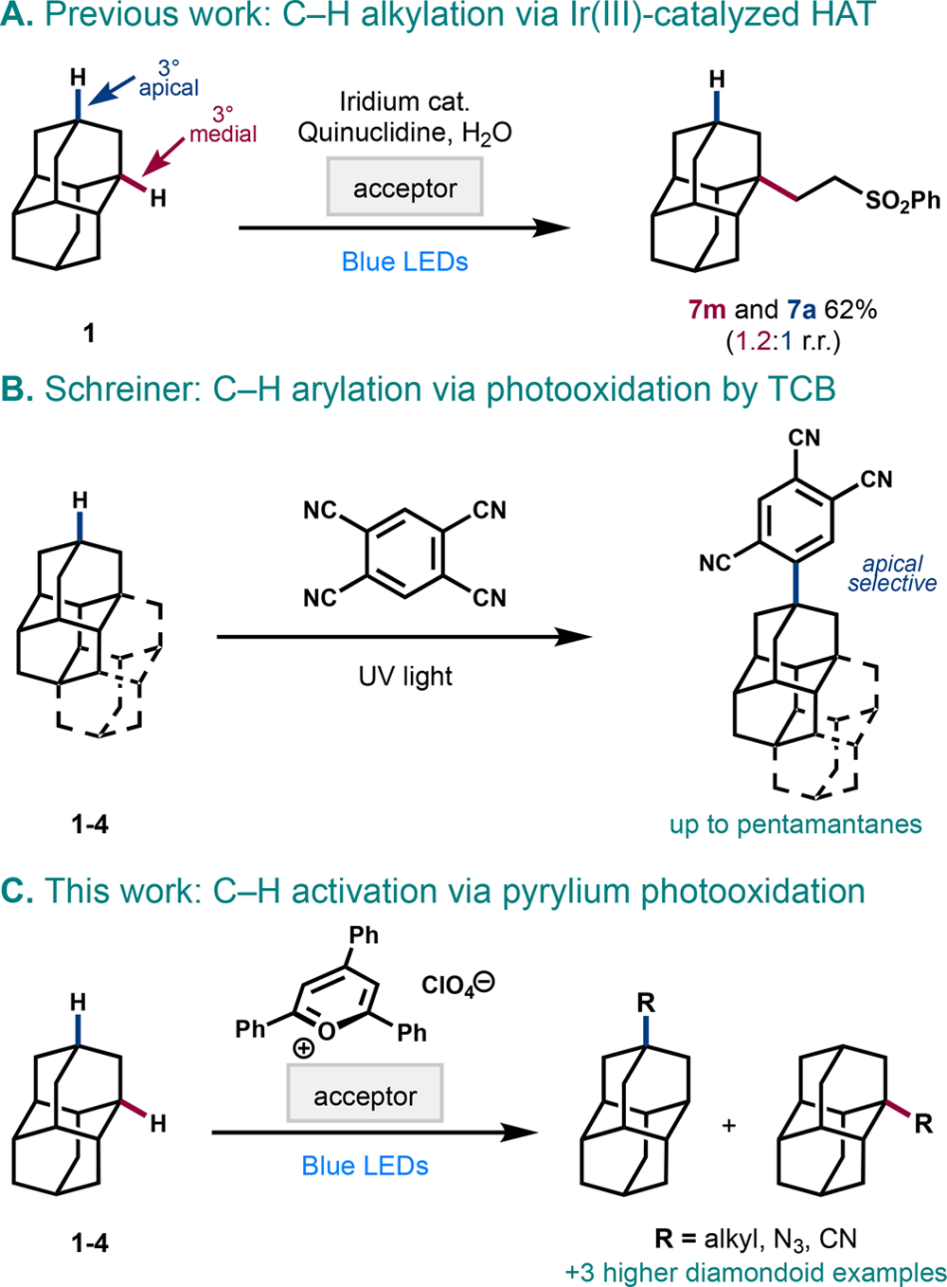
Regioselective Functionalizations of Diamantane and Higher Diamondoids, Including (A) Indirect HAT from Our Group,^8,9^ (B) Apical Arylation from Schreiner,^13^ and (C) the Proposed Functionalization Using Pyrylium Photocatalysis

In 2019 and 2021, our group reported two photocatalytic H atom transfer (HAT) methods enabling the tertiary (3°)-selective alkylation and aminoalkylation of adamantanes, even in the presence of substantially weaker, activated C–H bonds (including those present in ethers and secondary alcohols).^8,9^ The selectivity imparted by the quinuclidine cocatalyst did not translate to high regioselectivity with diamantane (**1**), which undergoes alkylation to give a 1.2:1 ratio of medial/apical products (**7m** and **7a**) under these conditions (Scheme 1A). Other reports of HAT-based methods to directly convert diamondoid C–H bonds to C–C bonds, such as the cyanation reaction using the phthalimido N-oxyl (PINO) radical reported by Schreiner and co-workers,^10^ frequently report functionalization of the medial position as the favored outcome. The photoacetylation of higher diamondoids, also reported by Schreiner, Fokin and co-workers, is a unique example of high selectivity favoring the apical position.^11^ In this case, apical selectivities of 82% up to >95% were attributed to higher polarizability through the apical position based on computational studies. A general, apical-selective HAT manifold remains elusive.

One distinctive feature of diamondoids is their ionization potentials, which decrease with increasing diamondoid size (Figure 1), potentially allowing for unique reactions under oxidative conditions.^12^ For instance, building off the work of Albini on adamantane and simpler hydrocarbons, Schreiner reported a fully apical-selective arylation of diamantane and higher diamondoids using 1,2,4,5-tetracyanobenzene (TCB) under UV light (Scheme 1B).^13,14^ This method was proposed to proceed via direct oxidation of the diamondoid by the singlet excited state TCB^*1^ (*E*_1/2_ = +3.4 V vs SCE in CH_3_CN), followed by selective proton loss from the less hindered apical position, due to elongation of the radical cation along the longer axis.^13,15^ This leads to the apical radical that is captured by TCB^•**–**^, and loss of cyanide delivers the observed product.

We wondered if we could facilitate apical-selective transformations *catalytically* on diamantane and other rare diamondoids by using a sufficiently oxidizing photoredox catalyst under visible light (Scheme 1C). This strategy would decouple the oxidation step from the radical trapping partner, allowing for additional C–C bond forming reactions beyond acetylation and arylation. For this, we envisioned the application of both a highly oxidizing organic photocatalyst^16^ to generate a transient radical cation and a Brønsted base to facilitate deprotonation, leading to apical radical functionalization. The proposed method has precedent in other photocatalytic oxidation/deprotonation processes reported by Wu and Hande, which enable alkylation of moderately activated C–H partners (e.g., allylic, benzylic, etc.).^17,26^ Herein we report the unexpected selectivity outcomes of our investigations of this strategy.

## Results and Discussion

**Scheme 2:**
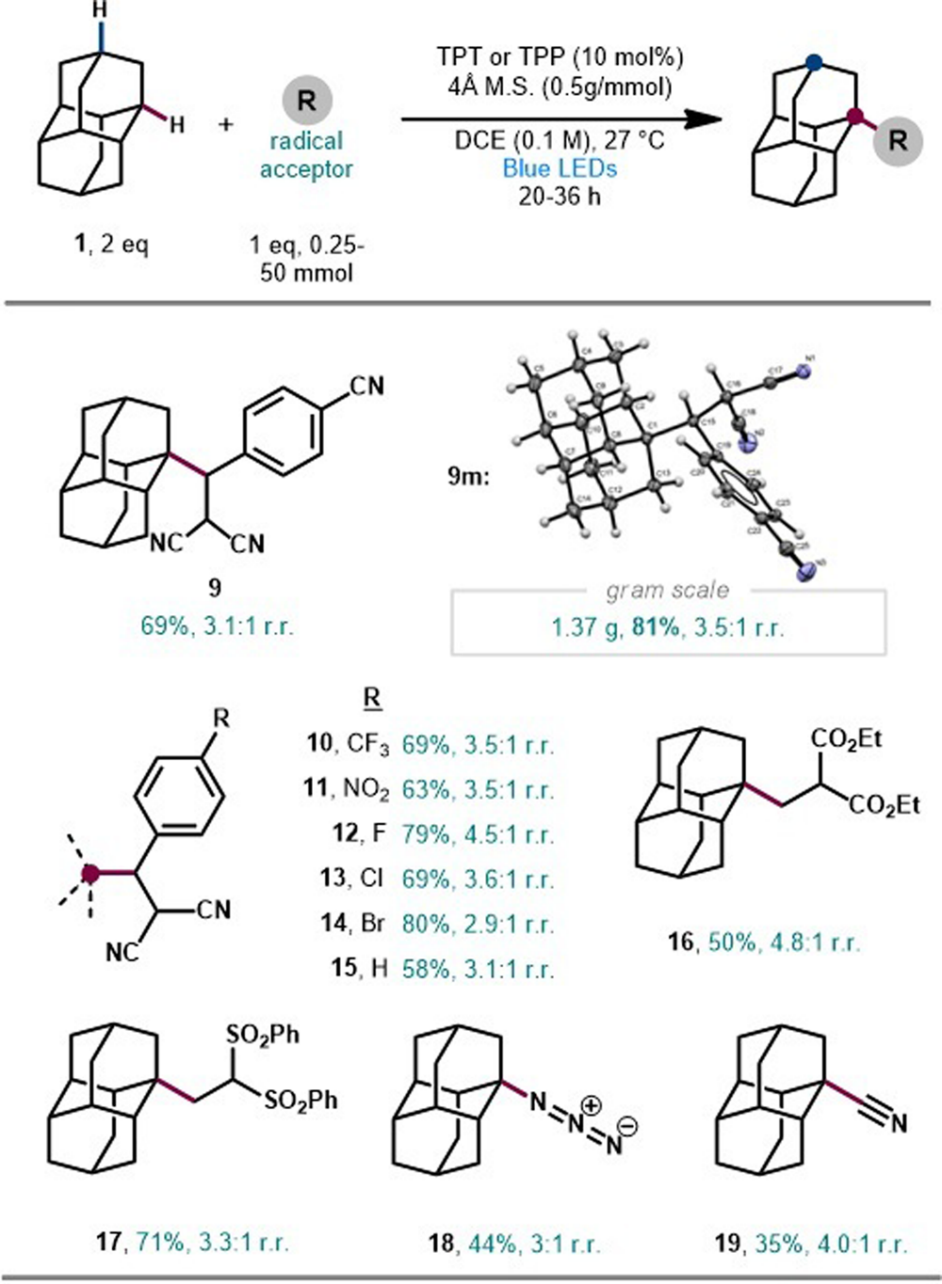
Substrate Scope of Diamantane Functionalization

Our initial exploration of a new diamondoid functionalization manifold commenced with the selection of a suitable radical acceptor for alkylation under the highly photooxidative conditions necessary for radical cation formation. Initial optimization identified suitable conditions with bis(phenylsulfonyl)ethylene as the radical acceptor, showing that the desired transformation was possible (see Table S2). This substrate presented challenges in reliably determining the product ratio and isolating clean products (namely, sulfone **17**, Scheme 2), which led us to shift toward benzylidene malononitriles as the radical acceptor (see Supporting Information for details). Using *p*-cyanobenzylidene malononitrile **8**, the alkylated product **9** (Table 1) could be isolated in clean form by aqueous workup and chromatographic purification, but we were surprised to see that a mixture of regioisomers was formed rather than only the apical product. Using nuclear magnetic resonance (NMR) techniques such as nuclear Overhauser effect (NOE) correlation and single crystal X-ray diffraction (XRD) of malononitrile **9m**, we confirmed that these conditions favor the medial product shown, in a regioisomeric ratio (rr) of ∼3:1 or higher. This unexpected result was consistent across all substrates and conditions described below and roughly matches the statistical ratio expected for 2 apical C–H bonds and 6 medial C–H bonds in diamantane (See Figure S1).

**Table 1:**
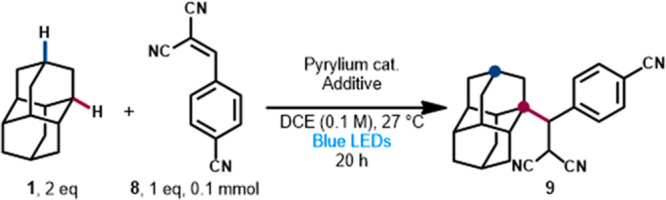
Optimization Studies with Diamantane^a^

entry	pyrylium, amount (mol %)	deviation	yield of **9** (%)	rr (M/A)
1	**A**, 5 mol %	1 equiv of NaF	61	4.3:1
2	**A**, 5 mol %	1 equiv of Li_2_CO_3_	47	4.8:1
3	**A**, 5 mol %	1 equiv of K_2_CO_3_	15	2.8:1
4	**B**, 10 mol %	2,6-lutidine	11	2.0:1
5	**A**, 5 mol %	no base	67	5.7:1
6	**A**, 5 mol %	glovebox setup	98	5.5:1
7	**A**, 5 mol %	100 mg of 3 Å sieves	65	8.3:1
8	**A**, 5 mol %	100 mg of 4 Å sieves	81	4.4:1
9	**A**, 5 mol %	100 mg of 5 Å sieves	63	3.8:1
10	**B**, 10 mol %	100 mg of 4 Å sieves	78	3.9:1
11	**B**, 10 mol %	50 mg of 4 Å sieves	84	3.9:1
12	**B**, 10 mol %	250 mg of 4 Å sieves	78	3.9:1
13	no cat.	50 mg of 4 Å sieves	0	
14	**B**, 10 mol %	no light	0	
15	**B**, 10 mol %	under air	70	3.3:1

a Reactions performed on a 0.1 mmol scale using 2 × 40 W 456 nm lamps over 20 h with fan cooling. Yields and rr determined by ^1^H NMR using phenanthrene as an internal standard.
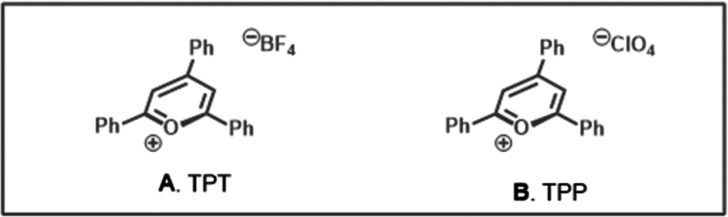

In optimizing the visible-light-induced photocatalytic reaction between diamantane (**1**) and acceptor **8**, we quantified the formation of medial and apical diamantyl benzylmalononitriles **9m** and **9a** while varying catalyst structure and loading, bases, and desiccation methods (Table 1). Initial conditions with NaF yielded a modest 61% conversion in 20 h (entry 1). While we initially hypothesized that the Brønsted base would induce neutral radical formation, other inorganic and organic bases delivered much lower efficiencies (entries 2–4). Higher yields were achieved when base was omitted entirely, suggesting that a base is not necessary (entry 5). We suspected that moisture sensitivity was an issue. To our satisfaction, weighing the reagents in a glovebox resulted in a 98% NMR yield (entry 6), affirming our suspicion that the conditions are moisture-sensitive. We proceeded to optimize with freshly activated molecular sieves as a more practical alternative (entries 7–9) and found that the 4 Å pore size led to the highest efficiency, at 81%.

Results from control reactions shed light on aspects of the mechanism. As expected, the omission of both the catalyst and blue light led to shutdown of the reaction (entries 13 and 14), affirming the necessity of the excited state photocatalyst in the process. Interestingly, it was found that running the reaction under air led to a respectable 70% yield (entry 15), suggesting that oxygen does not deactivate the excited state pyrylium proposed to be responsible for oxidation. Ultimately, optimized conditions consisting of 10% catalyst loading of 2,4,6-triphenylpyrylium perchlorate (TPP) and the addition of 4 Å molecular sieves (0.5 g/mmol substrate) in DCE consistently delivered the highest yields.

With the optimized conditions established, we turned our attention toward the preparative synthesis of functionalized diamondoids from a series of electrophilic radical acceptors (Scheme 2). 4-Cyanobenzyl malononitrile **9** was isolated in 69% yield with a 3.1:1 medial/apical product ratio. Other benzylidenemalononitriles with various arene ring substituents were well incorporated onto diamantane, including trifluoromethyl (**10**, 69% yield), nitro (**11**, 63% yield), halo (**12**–**14**, 69–80% yield), and unsubstituted phenyl (**15**, 58% yield), with regioisomeric ratios ranging from 2.9:1 to 4.5:1. Among other radical acceptors explored, a simple alkylidene malonate coupled efficiently, yielding diester **16** in 50% yield (4.8:1 rr). Bis(phenylsulfonyl)ethylene provided product **17** in 71% yield with a medial/apical ratio of 3.3:1. This method was amenable to scale-up without major modification. The synthesis of malononitrile **9** was slightly more efficient on gram-scale, proceeding in 81% yield and 3.5:1 rr. Overall, the rr’s in Scheme 2 match the ratio of C–H bond types or slightly favor the medial position (>3:1).

Beyond Giese-type radical acceptors, the successful coupling with other functional groups was limited. Inspired by Alexanian’s azidation method, azide **18** was synthesized in 44% yield with a 3:1 rr.^19^ Similar to Schreiner and co-workers’ cyanation approach, *p*-toluenesulfonyl cyanide was converted to diamantyl cyanide **19** in a 35% isolated yield and 4.0:1 ratio.^10^ A number of radical functionalizations including trifluoromethiolation, acylation, and borylation were unsuccessful under these conditions (see Supporting Information for details).

**Scheme 3:**
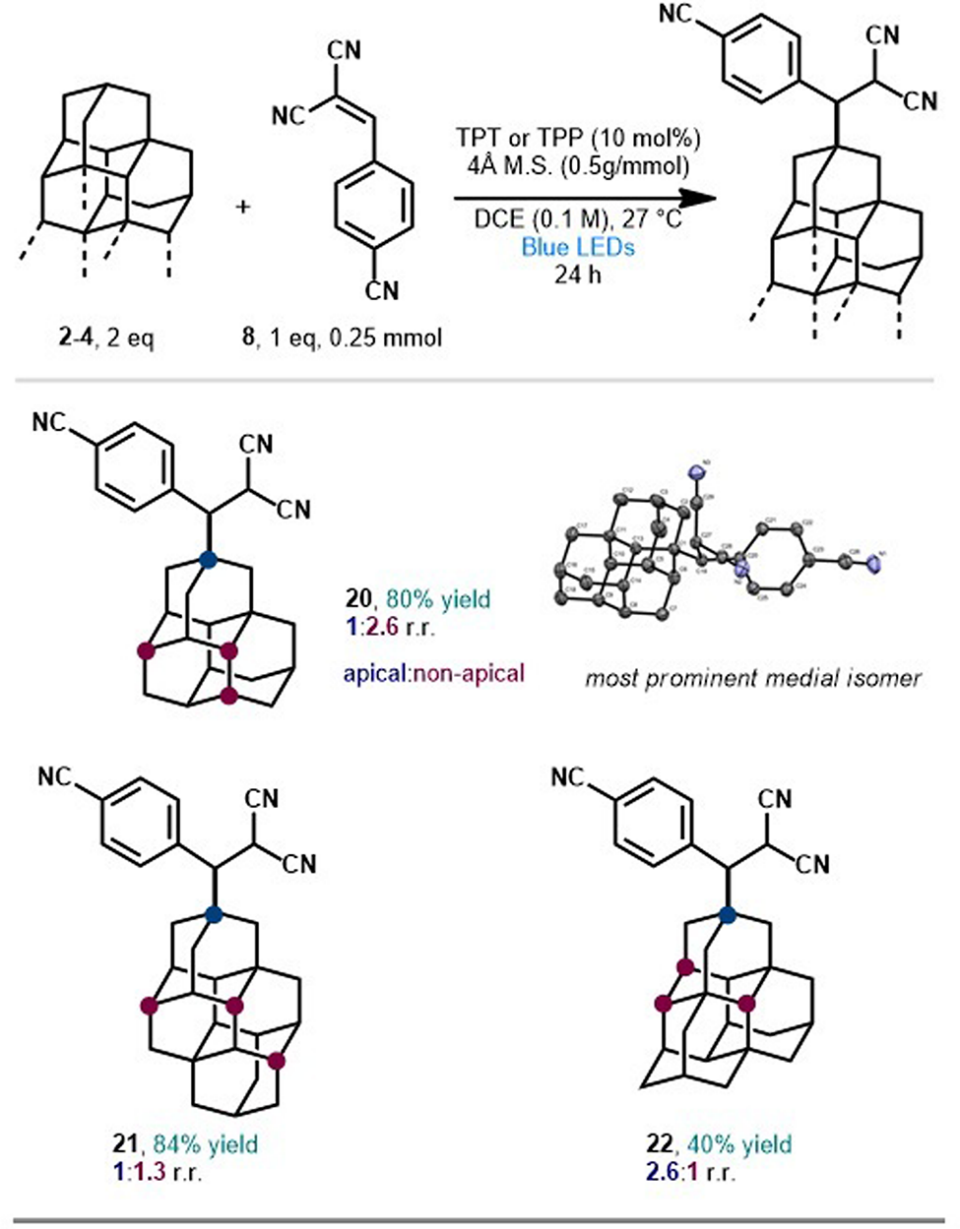
Tertiary-Selective Alkylation of Higher Diamondoids

Gratifyingly, higher diamondoids were also amenable to alkylation with malononitrile **8** under our optimized conditions (Scheme 3). Triamantane underwent alkylation to yield triamantyl malononitrile **20** in 80% yield. Chromatographic separation of individual regioisomers, followed by structural confirmation using NOE-NMR and XRD, revealed a final product ratio of 1:2.6 apical/nonapical. Rare tetramantane isomers, [121]tetramantane and [1(2)3]tetramantane, were efficiently alkylated in 84% and 40% yield, providing **21** and **22**, with 1:1.3 and 2.6:1 apical/nonapical ratios, respectively. These heightened apical/nonapical ratios deviate significantly from the statistical distribution of C–H bond types (2 apical and 8 medial for triamantane, 2 apical and 10 medial for [121]tetramantane, 3 apical and 7 medial for [1(2)3]tetramantane; see Figure S1). This selectivity favoring the apical position is consistent with previous functionalizations of these diamondoids^18^ and correlates with increased steric hindrance of the medial positions in higher analogs. Separation of isomeric products is quite challenging, and limited access to the starting materials prevented further optimization of our protocols to improve the yield of tetramantanes, such as **22**. Notably, all alkylated nanodiamond products in Schemes 2 and 3 are reported for the first time, to the best of our knowledge, highlighting the utility of this approach.

**Figure 2:**
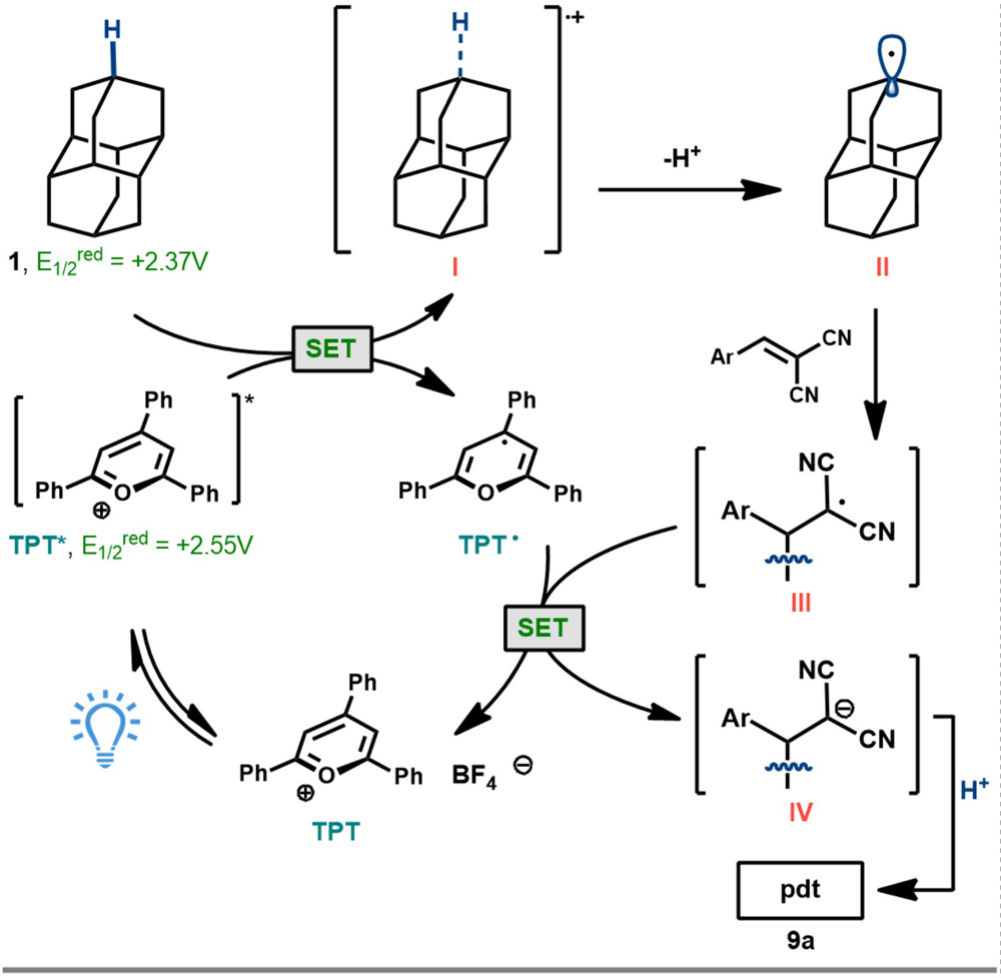
Proposed mechanism of photocatalytic activation.

The proposed mechanism for diamantane functionalization is presented in Figure 2, beginning with the excitation of the ground-state 2,4,6-triphenylpyrylium tetrafluoroborate (TPT) by visible light to the highly oxidizing excited state TPT* (*E*_1/2_^red^ = +2.55 V vs SCE in CH_3_CN).^20^ This leads to the outer-sphere oxidation of diamantane (*E*_1/2_^red^ = +2.37 V vs SCE)^21^ and the generation of diamantyl radical cation **I** and TPT pyranyl radical. Deprotonation of the diamantyl radical cation results in diamantyl radical **II** (apical shown), which is captured by the π-acceptor to form the carbon-centered radical intermediate **III**. Turnover by reduction from the triarylpyranyl radical (*E*_1/2_^red^ = −0.13 V vs SCE) and subsequent protonation of **IV** yields the alkylated diamantane **9a**. While the pathway for the apical product **9a** is illustrated, we rationalize that deprotonation under these conditions is unselective, leading to all possible tertiary radicals, although we cannot exclude other possibilities (vide infra).

**Figure 3:**
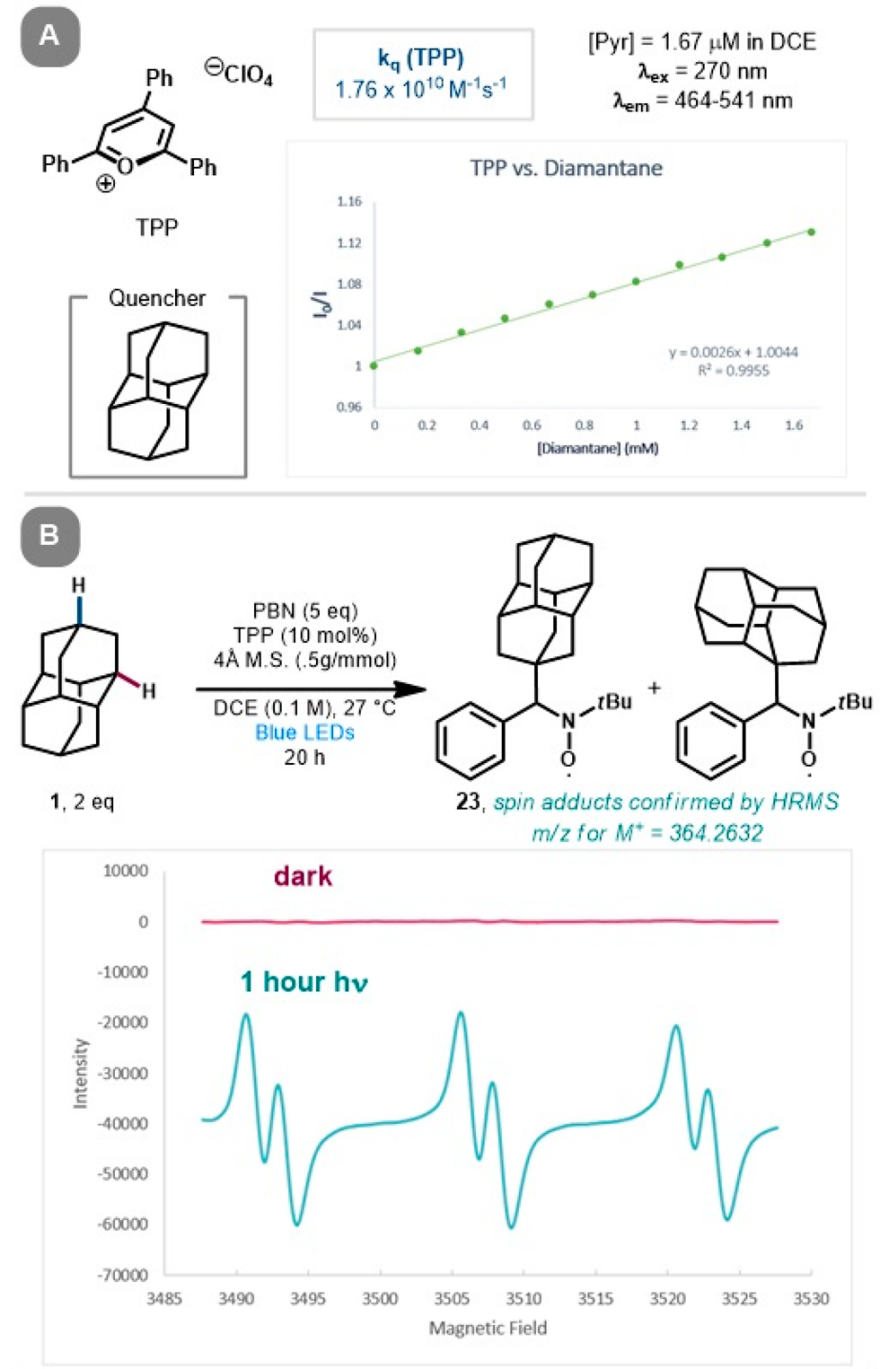
Mechanistic experiments, including (a) Stern–Volmer quenching studies and (B) EPR spectroscopic analysis and HRMS of spin-trapped species, supporting diamantane radical formation.

To support the proposed photooxidation mechanism by the pyrylium catalyst, Stern–Volmer luminescence quenching studies were conducted (Figure 3A). While there was no luminescence quenching by the alkylidene partner **8**, quenching of TPT by diamantane was observed, providing a positive linear slope, indicating diamantane as a competent quencher for the excited state of TPT. The calculated quenching rate constant (*k*_q_) of 2.07 × 10^10^ M^–1^ s^–1^ reflects the attenuation of TPT’s fluorescence in the presence of diamantane. A slightly lower quenching rate was noted for TPP (*k*_q_ = 1.76 × 10^10^ M^–1^ s^–1^), reinforcing TPP’s comparable efficiency to TPT as a suitable catalyst. The weak quenching in Figure 3A reflects the very short excited state lifetime of TPT (4.38 ns) and the low concentrations of diamantane used, limited by the low solubility in DCE. Despite the reported oxidation potential of mesityldiphenylpyrylium tetrafluoroborate (MDPT) appearing to be strong enough (*E*_1/2_ = +2.62 V vs SCE),^20^ fluorescence quenching was comparatively very low and MDPT was not found to be an effective catalyst for this reaction, consistent with this result (see Supporting Information). To validate the oxidation of the diamondoids by TPT/TPP, we used cyclic voltammetry to reveal the oxidation potentials of diamantane (*E*_1/2_^red^ = +2.46 V vs SCE in CH_3_CN) and triamantane (*E*_1/2_^red^ = +2.18 V vs SCE), aligning closely with literature values (see Supporting Information). As expected, oxidation becomes more facile as the cage is extended.^13^

Electron paramagnetic resonance (EPR) spectroscopy with radical spin trapping using *N*-*tert*-butyl-α-phenylnitrone (PBN) was explored to support the presence of carbon-centered radicals during the photoreaction (Figure 3B). Running the reaction with **1** in the presence of PBN and light, with or without an alkylidene acceptor, led to a strong EPR signal, while no signal was observed in the dark. The characteristic hyperfine splitting of the observed spin adduct is consistent with a nitroxyl radical adduct derived from a carbon-centered radical (**23**).^22,23^ The formation of the spin adduct was confirmed by high-resolution mass spectrometry (HRMS), revealing a dominant peak at *m*/*z* 364.2632 corresponding to the oxoammonium ion of the spin adduct. These results are consistent with the proposed mechanism and demonstrate the dependency of radical formation on the interaction of **1** with the excited state TPT*, further supporting the direct oxidation of the diamondoid hydrocarbon by the photocatalyst.

With regard to the selectivity outcome observed, a number of factors could account for the formation of the medial product that was not observed in the arylation reaction that served as inspiration for this chemistry. Schreiner and Fokin proposed that the diamondoid radical cation elongates along the longer axis based on computational analysis, which weakens and acidifies the apical C–H bonds.^13^ Later computational work by Fokin showed that the interaction of radical cation **I** with nucleophilic solvent molecules leads to an alternative structure where medial C–H bonds are elongated and activated, consistent with experimental results showing medial selectivity using electrochemical oxidation in acetonitrile.^24,25^ Contribution of the latter effect could at least partially explain the selectivity outcome under the conditions of our reaction. Alternatively, regioselectivity in Albini’s previous work on the arylation of a variety of hydrocarbons with TCB was rationalized based on ion pairing between the arene radical anion and hydrocarbon radical cation. This ion pairing sterically blocked some positions while leaving other C–H bonds exposed for deprotonation, leading to a rationale for regioselectivity. If ion pairing is also important for the arylation of diamantane and higher diamondoids, then the association of TCB^•–^ around the medial belt could also direct deprotonation to the apical positions. In the case of oxidation by a pyrylium catalyst, the resulting uncharged triarylpyranyl radical is expected to be weakly coordinated and would not influence the regioselectivity of deprotonation. In the absence of this steric blocking effect and potentially in combination with the solvation effects described by Fokin, deprotonation at all tertiary sites would lead to a mixture of both apical and medial radicals, consistent with the outcomes presented here. It should be emphasized that the deviation from the statistical ratio of unique C–H types indicates some selectivity that varies from substrate to substrate, and under no circumstances did we observe functionalization at the secondary positions. At this point, we cannot exclude the possibility of an equilibration of the two radicals after deprotonation. Additional experimental evidence is necessary to probe this possibility and will be reported in due course.

## Conclusions

We have successfully developed a new 3°-selective C–H functionalization protocol for diamondoids using an oxidizing organic photocatalyst and electron-deficient radical acceptors. The reaction is effective for a range of activated acceptors and diamondoids up to tetramantane, with regioselectivities ranging from 4.8:1 to 1:2.6 medial/apical, depending on the identity of the two partners. The initially expected apical product was shown to be the minor isomer in all but one case. Experimental evidence supports the proposed mechanism of oxidation of the diamondoid followed by deprotonation, and solvation of the radical cation in combination with a lack of ion pairing likely contributes to the observed selectivity outcomes. Future research efforts will be focused on fully elucidating the factors governing the selectivity of C–H activation in diamantane and identifying a method that will enable more efficient access to the desirable apical products. Nevertheless, the catalytic functionalization of higher diamondoids with a simple organic photocatalyst under visible light is a significant development that enables direct access to these important hydrocarbon building blocks.

## Supporting Information Available

Experimental procedures, characterization data, ^1^H and ^13^C NMR spectra of all new compounds (PDF)
X-ray crystal structure data of **9m** (CIF)
X-ray crystal structure data of **20** (CIF)

## Supplementary Material

cs4c00296_si_001.pdf

cs4c00296_si_002.cif

cs4c00296_si_003.cif
