# Supplementary material for: Selective C–H Activation of Molecular Nanodiamonds via Photoredox Catalysis
Source: ACS Catal. 2024 Mar 1;14(6):4093–8. doi: 10.1021/acscatal.4c00296 (PMC10949193; doi:10.1021/acscatal.4c00296)
Supplement: Supplementary file 1 — cs4c00296_si_001.pdf [file cs4c00296_si_001.pdf]

Supporting Information

Selective C–H Activation of Molecular Nanodiamonds via Photoredox Catalysis

Hoang T. Dang[a], Henry T. O'Callaghan[a], Mikayla M. Wymore[a], Jennifer Suarez[a], and David B.C. Martin[a]*

*Corresponding Author : david-martin@uiowa.edu

[a]Department of Chemistry, University of Iowa,

Iowa City, Iowa 52242, United States.

Contents

General Methods: 3

A. General Procedures 5

I. General Procedure A: photochemical reactions with pyrylium salts (preparative scale): ... 5

II. Gram-scale synthesis of diamantyl malononitrile 7

III. Synthesis of benzylidene malononitrile derivatives 7

IV. Synthesis of 2,4,6-triarylpyrylium salts 8

B. Optimization Details 9

I. General procedure B: optimization with diamantane and benzylidene malononitriles: 9

II. Optimization results 11

II. Failed substrates and catalysts 20

C. Photochemical Reactions with Diamantane 21

D. Assignment of Regioisomers by 1D-nOe and XRD 30

I. Structural Elucidation using X-Ray Crystallography 30

II. 1-Dimensional nOe NMR Analysis 31

E. Mechanistic Experiments 34

I. Luminescence Quenching Experiment using Stern-Volmer Analysis 34

II. Electron Paramagnetic Resonance (EPR) Spectroscopy 39

III. Alkylation of allylic and benzylic C–H donors 42

IV. Cyclic Voltammetry 43

F. NMR Spectra 45

References 67

General Methods:

All reactions were carried using oven dried or flame dried glassware charged with a magnetic stir bar and conducted under an inert nitrogen atmosphere using typical Schlenk techniques, unless otherwise noted. 1,2-Dichloroethane (DCE) was purified by distillation from CaH_2 under argon. All other solvents were dried by passage through columns of activated alumina or distilled and stored under nitrogen over freshly activated 4 Å sieves or otherwise freshly distilled. All starting materials were prepared according to known literature procedures or used as obtained from commercial sources, unless otherwise indicated. Adamantane and diamantane purchased from commercial sources, while triamantane, [121]tetramantane, and [1(2)3]tetramantane were provided by Dr. Jeremy E. P. Dahl. Reactions were monitored by thin-layer chromatography (TLC) and carried out on 0.25 mm coated commercial silica gel plates (Analtech TLC Uniplates, F254 precoated glass plates) using UV light as visualizing agent. Unless otherwise indicated, silica gel chromatography was performed using flash chromatography on P60 silica. Alternatively, a Yamazen Smart Flash AI-580S system in conjunction with Yamazen Universal Premium 40g columns with the specified gradient elution mode was used when indicated.

^{1}H and ^{13}C NMR spectra were recorded on a Bruker Avance NEO 400, Bruker Avance III 500, or Bruker Avance III 700 MHz spectrometer and were internally referenced to residual protio solvent signal (note: $CDCl_3$ referenced at δ 7.27 ppm for ^{1}H NMR and δ 77.16 ppm for ^{13}C NMR, respectively). Data for ^{1}H NMR are reported as follows: chemical shift (δ ppm), multiplicity (s = singlet, d = doublet, t = triplet, q = quartet, m = multiplet, app=apparent), coupling constant (Hz), and integration. Data for ^{13}C NMR are reported in terms of chemical shift and no special nomenclature is used for equivalent carbons. Selective nOe experiments were performed with the standard Bruker pulse program, selnogp, employing a mixing time of 0.5s and a relaxation delay of 1 second. High-resolution mass spectrometry data were recorded on a Thermo Q-Exactive instrument using direct injection of samples in dichloromethane into the electrospray source (ESI) with positive ionization. Gas Chromatography was carried out using a Shimadzu GC-2010 Plus instrument equipped with a Shimadzu SH-Rxi-5ms column. Column Specifications: 15 m (L), 0.25 mm (ID), 0. 25 µm (d_f), (diphenyl/dimethyl polysiloxane) stationary phase. Luminescence quenching experiments were conducted using a Fluoromax-4 Spectro-Fluorometer with 0.50 mm slits opening.

Electron paramagnetic resonance (EPR) experiments were conducted utilizing state-of-the-art instruments available at the University of Iowa EPR facility. Specifically, a Bruker EMX ESR Spectrometer equipped with a 25 cm magnet, a 90 dB X-band bridge with a microwave frequency counter, and various cavity configurations (TE, TE-double, TM) was employed for high-precision EPR measurements. The instrument featured a liquid nitrogen variable temperature system and a liquid helium variable temperature system to facilitate experiments over a wide range of temperatures. Additionally, a gaussmeter with an in-cavity probe was utilized to ensure accurate field measurements. In addition to the aforementioned system, an HS cavity with an aqua-X system was employed to enhance the versatility of the EPR experiments.

X-ray diffraction experiments were conducted using a Bruker D8 VENTURE DUO diffractometer featuring a IμS 3.0 microfocus source, which operated at 75 W to generate Mo Kα radiation ($\lambda = 0.71073$ Å). Crystals selected for XRD analysis were carefully transferred from their vials and placed on a glass slide in Cargille type NVH immersion oil. A Zeiss Stemi 305 microscope was used to identify suitable specimens from representative material samples. The crystals were collected on a MiTeGen 100-micron MicroLoop and then transferred to the instrument. During data collection, the sample remained under a continuous cold nitrogen stream, maintained at a stable temperature of 100K using an Oxford 800 series cryostat. The collected data were corrected for various factors using the APEX41 software, and numerical absorption and semi-empirical adsorption corrections were applied with SADABS1. SHELXT2 and SHELXL3 within OLEX2 software were employed for initial structure solution and refinement, respectively. Geometric constraints were used for hydrogen atoms bound to carbon atoms.

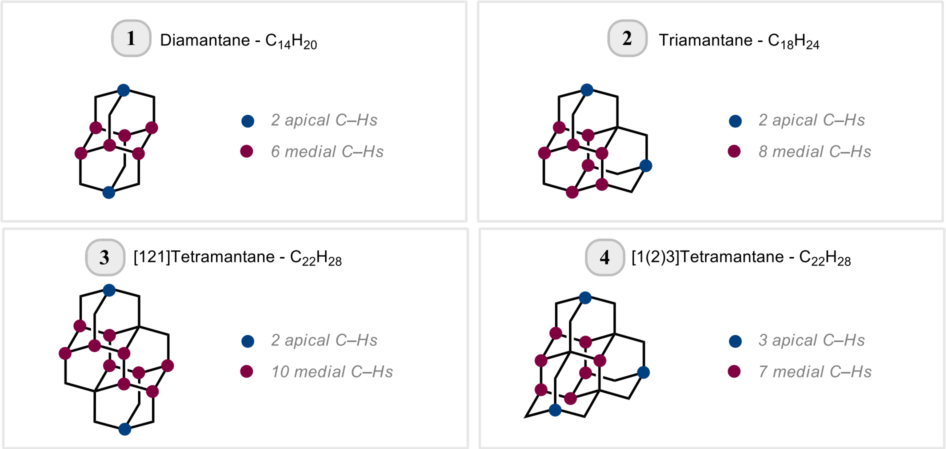

Figure S1. Types of 3° diamondoid C–H bonds.

Described in **Figure S1** are the types and numbers of 3° C–H bonds in diamantane (**1**), triamantane (**2**), [121]tetramantane (**3**), and [1(2)3]tetramantane (**4**) . Diamantane contains 6 medial C–H bonds around its medial belt and 2 apical C–H bonds, establishing a 3:1 statistical ratio of medial:apical C–H bonds. Triamantane owns 8 medial C–H bonds, [121]tetramantane owns 10 medial C–H bonds, and [1(2)3]tetramantane owns 7 medial bonds. Diamondoids **1**, **2**, and **3** all possess 2 apical C–H bonds, whereas **4** uniquely contains 3 apical C–H bonds.

A. General Procedures

I. General Procedure A: photochemical reactions with pyrylium salts (preparative scale):

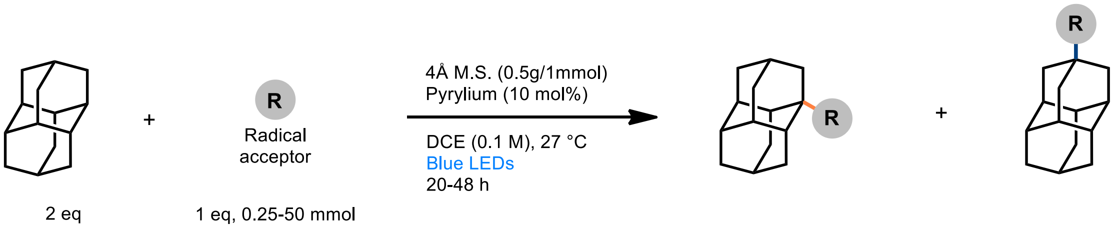

A typical procedure for a photoreaction with diamondoids can be conducted as follows: Diamondoid (1.0 mmol, 2.0 equiv.), pyrylium catalyst (10 mol%), radical acceptor (0.50 mmol, 1.0 equiv.), activated 4Å molecular sieves (125 mg), and dry 1,2-dichloroethane (5.0 mL) were added to an 8-mL vial equipped with a super-micro stir bar. The vial was sealed with a Teflon septum and screw-top cap, and further sealed with parafilm. The resulting mixture was degassed by a freeze/pump/thaw operation: freezing the vial contents in a dry ice/acetone bath, placing the vial under a static vacuum, then allowing the frozen mixture to thaw then backfilled with N_2 (this

cycle was repeated 3 times). The vials were then stirred at room temperature for 20-48h. The reaction mixture was then reduced to dryness under vacuum and the resulting residue was purified using column chromatography (product dry loaded on silica support followed by elution with 50-150 mL hexanes then 5-15% EtOAc/Hexanes) to give a beige or pale white solid as a mixture of regioisomers. Product fractions that are contaminated by remnant aryl aldehyde were combined and reduced to dryness under vacuum, and then subjected to procedure **S4** (see *Optimization Details*). All isolated diamondoid products were further characterized by ^{1}H and ^{13}C nuclear magnetic resonance (NMR) spectroscopy, infrared spectroscopy, and mass spectrometry.

II. Gram-scale synthesis of diamantyl malononitrile

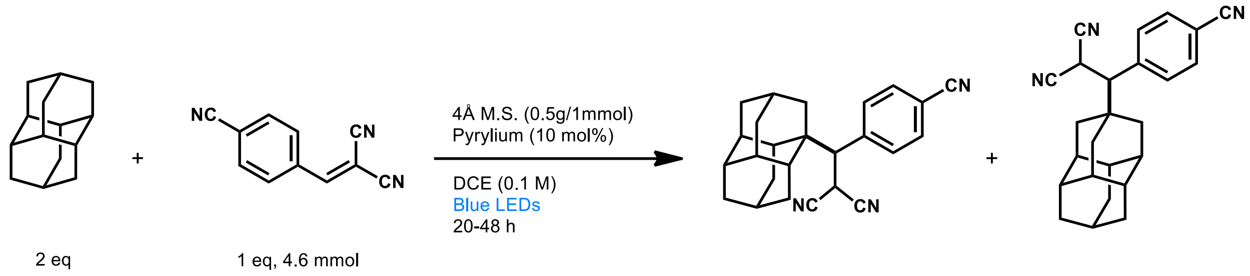

A typical procedure for a photoreaction with diamondoids can be conducted as follows: Diamondoid (13.8 mmol, 3.0 equiv.), pyrylium catalyst (10 mol%), radical acceptor (4.6 mmol, 1.0 equiv.), activated 4Å molecular sieves (2.8 g), and dry 1,2-dichloroethane (46.0 mL) were added to a 50-mL Schlenk tube equipped with a micro stir bar. The resulting mixture was degassed by a freeze/pump/thaw operation: freezing the vessel contents in a dry ice/acetone bath, placing the vial under a static vacuum, then allowing the frozen mixture to thaw then backfilled with N_2 (this cycle was repeated 3 times). The vessel was then stirred at room temperature for 60h. The reaction mixture was then reduced to dryness under vacuum and the resulting residue was purified using column chromatography (product dry loaded on silica support followed by elution with 500 mL hexanes then 5-15% EtOAc/Hexanes) to give **5** (1.37 g, 81% yield, 3.5:1 medial:apical) as a mixture of regioisomers.

III. Synthesis of benzylidene malononitrile derivatives

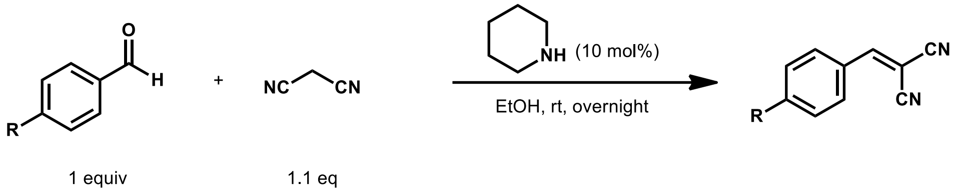

The synthesis of benzylidene malononitriles were adapted from known Knoevenagel condensation conditions. To a round bottom flask equipped with a magnetic stir bar were added malononitrile (7.00 mmol, 1 equiv.) and ethanol (7 mL). Aryl aldehyde (8.40 mmol, 1.2 equiv.) was added followed by dropwise addition of piperidine. In some cases, the omission of piperidine led to a cleaner reaction, so this reagent could be omitted. The reaction mixture was stirred at room temperature for 30 minutes or until the mixture is clouded with precipitate. The solids were then collected via vacuum filtration and rinsed with a cold solution of hexane/ethanol (V/V＝10:1). If

necessary, recrystallization of the benzylidene malononitrile product is typically done with 10% acetone/ethanol.

IV. Synthesis of 2,4,6-triarylpyrylium salts

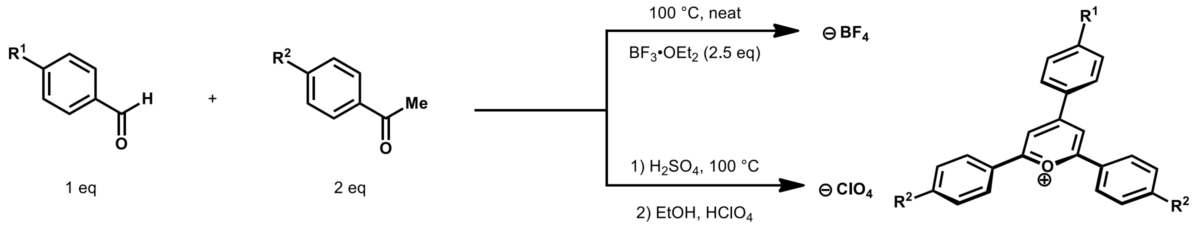

The procedure for the synthesis of 2,4,6-triarylpyrylium salts was adapted from previously reported procedures.[1] In a round-bottom flask equipped with a magnetic stir bar, acetophenone (2 eq) was mixed with benzaldehyde (1 eq) at room temperature and then heated to 100 °C, adding a few drops of $HClO_4$ and stirring for 30 minutes. The mixture was cooled to room temperature, followed by addition of acetone (3 mL / 1 mmol) and then precipitation of the product with copious amounts of diethyl ether. The bright-yellow pyrylium salt obtained was washed with cold acetone and diethyl ether. Recrystallization of the pyrylium salts can be done with DCE with minimal drops of DCM.

B. Optimization Details

I. General procedure B: optimization with diamantane and benzylidene malononitriles:

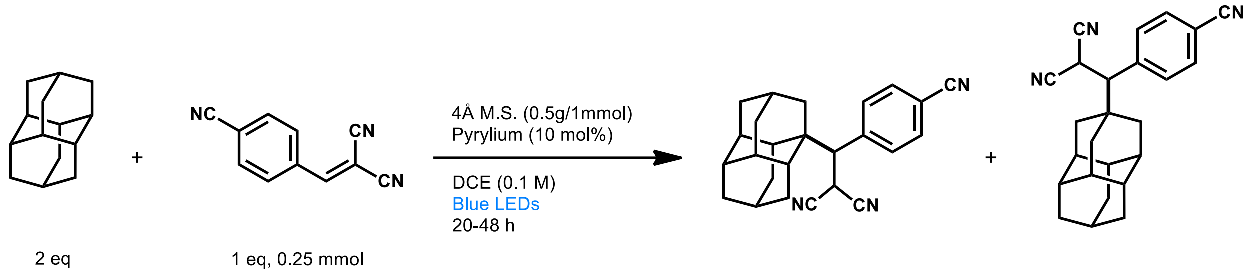

Diamantane (0.20 mmol, 2.0 equiv.), pyrylium catalyst (10 mol%), 4-cyanobenzylidene malononitrile (0.10 mmol, 1.0 equiv.), activated 4Å molecular sieves (50 mg), and dry DCE (1.0 mL) were added to an 8-mL vial equipped with a super-micro stir bar. The vial was sealed with a Teflon septum and screw-top cap, and further sealed with parafilm. The resulting mixture was degassed by a freeze/pump/thaw operation 3 times. The vials were then stirred at room temperature for 20-48h and were irradiated by Kessil LED lamps.

B1. Addition of internal standard for ^{1}H NMR assay:

Phenanthrene (0.10 mmol) dissolved in $CDCl_3$ was added to the crude reaction mixture, followed by thorough mixing via pipette. ~5 Drops of this homogenized sample were transferred to an NMR tube and diluted with $CDCl_3$. An NMR product yield was calculated based on diagnostic methine C–H peaks (medial methine (1H): δ4.17 ppm; apical methine (1H): δ4.28 ppm) relative to phenanthrene (δ8.71, 2H). % Remaining starting material was calculated based on benzylidene @ δ8.00 (1H).

If product R_f overlaps with that of alkylidene, mixture can be treated with $P(OEt)_3$/PMA protocol (see **B2**) followed by column chromatography (**B3**).

*If alkylidene is fully consumed or does not pose issues during purification, skip to column chromatography (**B3**).*

B2. Reaction to remove alkylidene (optional):

Triethyl phosphite (>0.10 mmol) was added to the crude reaction mixture followed by dropwise addition of phosphomolybdic acid (PMA, 20 mol%) at room temperature. The mixture turned dark blue-green and was stirred 30-90 minutes (see referenced protocol for stir durations depending on alkylidene)[2]. The mixture was transferred to a separatory funnel

and washed with H_2O (>5 mL) and extracted with 5-10% EtOAc/Hexanes (3 X 25 mL). The organic layer was dried (Na_2SO_4), filtered, and concentrated in vacuo, followed by purification by silica gel column (**B3**).

Alternatively, if you are treating product fractions contaminated with alkylidene (following chromatographic purification, **B3) with $P(OEt)_3$/PMA, then resulting crude residue can be subjected to short silica plug (eluted with 10% EtOAc/Hexanes) to yield pure product.*

B3. Column purification procedure:

The reaction mixture (**B1**) or organic layer (**B2**) was then reduced to dryness under vacuum and the resulting residue was purified using column chromatography (product dry loaded on silica support followed by elution with 50-150 mL hexanes then 5-15% EtOAc/Hexanes) to give a beige or pale white solid. Product fractions that are contaminated by remnant aryl aldehyde were combined and reduced to dryness under vacuum, and then subjected to procedure **B4**:

B4. Workup to remove aryl aldehyde byproduct (optional):

The following protocol is based on a liquid-liquid extraction method referenced herein.[3] The resulting mixture was dissolved with minimal methanol (2-5 mL), transferred quantitatively to a separatory funnel, then saturated sodium bisulfite (*aq.*) added, shaken for 30-60 s, diluted with 10 mL of water, and extracted with 10% EtOAc/Hexanes (3 X 10 mL). The organic layer was dried (Na_2SO_4), filtered, and concentrated in vacuo to yield the recovered, pure product.

II. Optimization results

Table S1. Optimization with benchtop desiccants

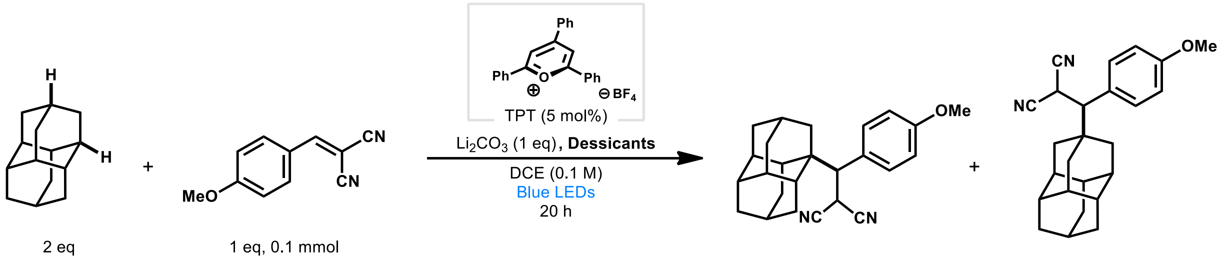

Reaction	Deviation	Time (hr)	% Alkene	% Yield	r.r.
a	-	1	98	0	-
b	-	24	58	25	7.3 : 1
c	5 eq H_2O	24	87	trace	-
d	3 eq Na_2SO_4	18	33	30	9 : 1
e	3 eq $MgSO_4$	18	47	21	9.5 : 1
f	4Å MS	18	34	29	8.7 : 1
g	No Li_2CO_3	22	71	21	4.3 : 1
h	TPT = 0 mol%	24	<100	trace	-
i	10 mol%	24	35	36	8 : 1
j	20 mol%	24	48	36	8 : 1
k	30 mol%	24	50	36	5 : 1

Product yield determined by ^{1}H NMR using dibenzyl ether as an internal standard. Medial product: δ4.12; apical product: δ4.23.

The starting conditions included 1 equivalent of Li_2CO_3 as a base and featured the 4-methoxybenzylidene malononitrile as the radical acceptor. After reacting for 24 hours, the reaction efficiency was recorded at about 25% (b). There was no product identified after 1 hour of reaction time (a), suggesting that this photochemical reaction occurs very slowly. Interestingly, addition of 5 equivalents of water completely shut down the reaction (c), affirming our early suspicions that this reaction is moisture-sensitive. Benchtop desiccants had little effect on reaction efficiency (reactions d-f). It is important to note that the 4A molecular sieves used in this reaction were briefly activated by flame-dry under vacuum for a few minutes; molecular sieves were revisited with more rigorous activation later (**Table S1**). Interestingly when the base was removed, 21% of the product was still formed (g), suggesting that base may not be necessary for the reaction. When the TPT photocatalyst was omitted from the reaction, trace product was observed with almost full recovery of the alkene (h). Reactions with 10% or more TPT were capped at 36% yield (i-k). Given the

reaction's presumed sensitivity to water, we opted to probe the catalyst loading once desiccation is improved.

Table S2. Optimization with bis(phenylsulfonyl)ethylene acceptor.

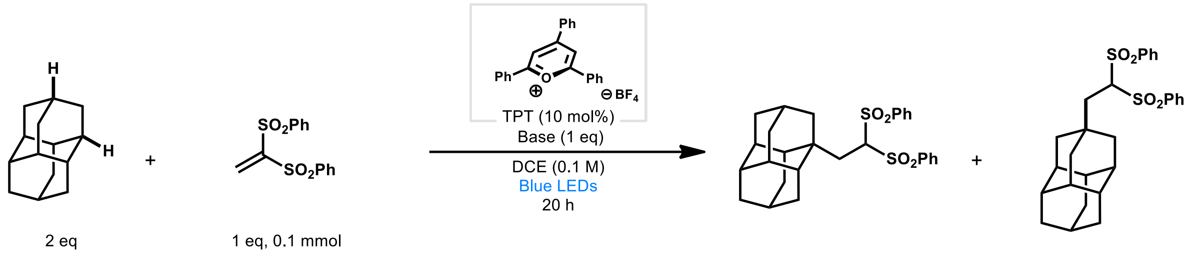

Reaction	Inorganic Base	% Yield	r.r.
a	NaF	21	9.9 : 1
b	NaOAc	57	5.2 : 1
c	$NaHCO_3$	42	4.8 : 1
d	Na_2CO_3	30	1.5 : 1
e	K_2CO_3	39	11 : 1
f	Li_2CO_3	66	3.8 : 1
h	No base	40	4.7 : 1

Product yield determined by GC using dibenzyl ether as an internal standard. Relative response factors for the medial and apical products (respectively) were calculated as follows: 0.394 and 0.352.

As shown in **Table S2**, bis(phenylsulfonyl)ethylene was a compatible radical acceptor paired with a variety of inorganic bases (a-f), with lithium carbonate providing the highest GC yield. Removal of base led to a solemn 40% yield (h). Alkene was not observed on the GC, likely due to substrate decomposition with temperatures above 100 °C.

Table S3. Effects of inorganic and organic bases

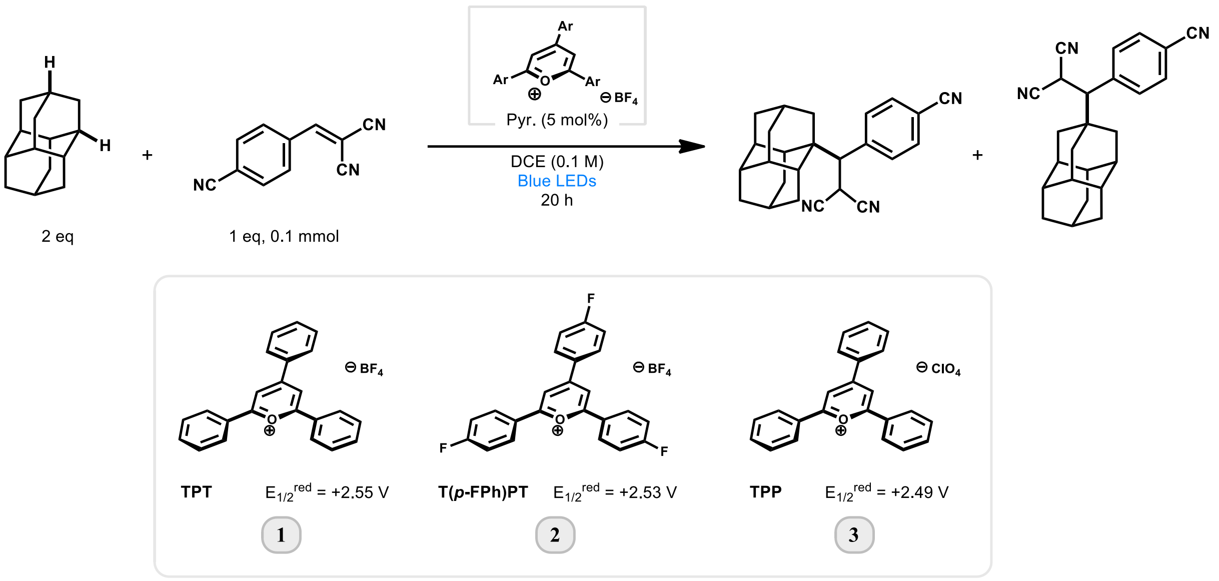

Reaction	Pyrylium	Inorganic Base	% Yield	r.r.
a	1	None	67	5.7 : 1
b	2	None	71	4.5 : 1
c	1	Li_2CO_3	47	4.8 : 1
d	2	Li_2CO_3	55	4.5 : 1
e	1	Na_2HPO_4	20	4.0 : 1
f	1	Na_2CO_3	45	4.6 : 1
g	1	K_2CO_3	15	2.8 : 1
h	1	NaF	61	4.3 : 1
i	3	Pyridine	12	3.0 : 1
j	3	Imidazole	0	-
k	3	2,6-Lutidine	11	2.0 : 1

Product yield determined by ^{1}H NMR using dibenzyl ether as an internal standard. Medial product: δ4.17; apical product: δ4.28.

As is evident in **Table S3**, the addition of inorganic base decreases the reaction efficiency. The omission of base with TPT and T(p-FPh)PT (pyryliums 1 & 2, respectively), led to gratifyingly high yields of 67-71% (a & b). Addition of lithium carbonate led to a decrease in the yield range to 47-55% (c & d). Other bases with varying degrees of hygroscopicity resulted led to even lower yields of alkylated diamantane (e-g). Of all the inorganic base reagents, sodium fluoride led a moderate yield of 61% (h), though this still does not surpass the reaction outcome with no base. Knowing that this reaction is moisture-sensitive, it is speculated that the addition of

some bases (all with varying degrees of hygroscopicity) may influence yields indirectly by acting either as drying agents or by introducing water into the reaction. Overall, the inorganic bases led to an average ratio of 4.2:1 medial:apical products.

Some organic amines were also tried as base additives, albeit to minimal efficiency. Pyridine afforded a 12% yield and a medial:apical ratio of 3:1 (i), whereas 2,6-lutidine led to a slightly lower 11% yield and an even lower ratio of 2:1 (k). Imidazole did not facilitate any observable product formation.

Table S4. Effects of arene substituents

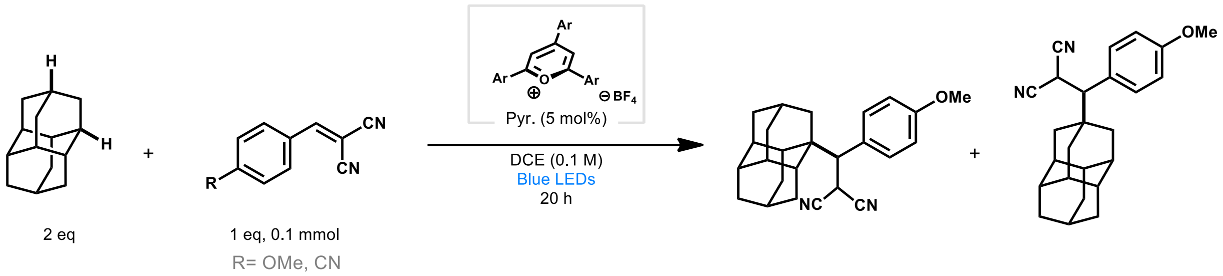

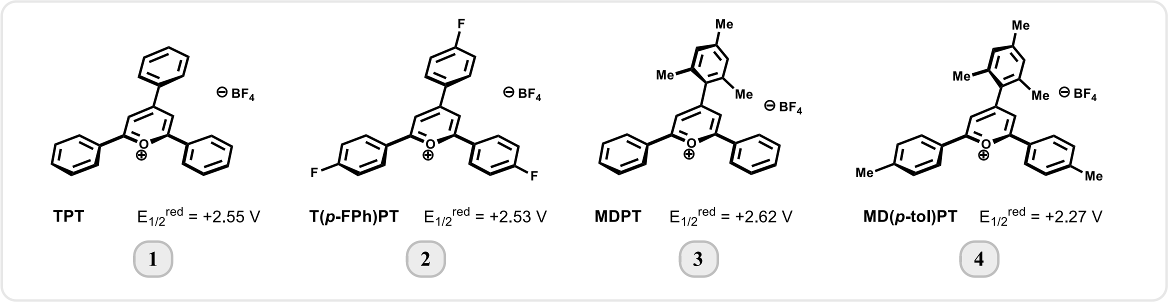

Reaction	R	Pyrylium	% Alkene	NMR Yield (%)	r.r.
a	CN	1	25	67	5.7 : 1
b	CN	2	23	63	5.3 : 1
c	CN	3	88	4	-
d	CN	4	95	trace	-
e	OMe	1	0	55	6.9 : 1
f	OMe	2	26	41	7.2 : 1
g	OMe	3	11	49*	7.2 : 1
h	OMe	4	75	4	-

Product yield determined by ^{1}H NMR using dibenzyl ether as an internal standard. (CN) Medial product: δ4.17; apical product: δ4.28. (OMe): Medial product: δ4.12; apical product: δ4.23.

Reactions carried out with 4-methoxy and 4-cyano substituents on the arene of the radical acceptor revealed the reaction's innate affinity to electron-poor substituents (**Table S4**). TPT and its tris-*p*-fluoro variant (pyryliums 1 and 2, respectively) worked efficiently with both acceptors, with higher efficiency observed for the *p*-cyano substrate. In fact, we have observed on many occasions very similar reaction efficiencies to TPT when T(*p*-FPh)PT is used. MDPT and MD(*p*-tol)PT (pyryliums 3 and 4, respectively), worked with minimal efficiency, as shown in results for reactions c-d and h. Reaction g represents a rare case where MDPT led to significant product formation (NMR yield of 49%), however this result was not reproducible with MDPT. Therefore, despite it having a sufficient oxidation potential for this system, it was concluded to not be a compatible catalyst in these conditions.

Table S5. Effects of anhydrous atmosphere and rigorous desiccation

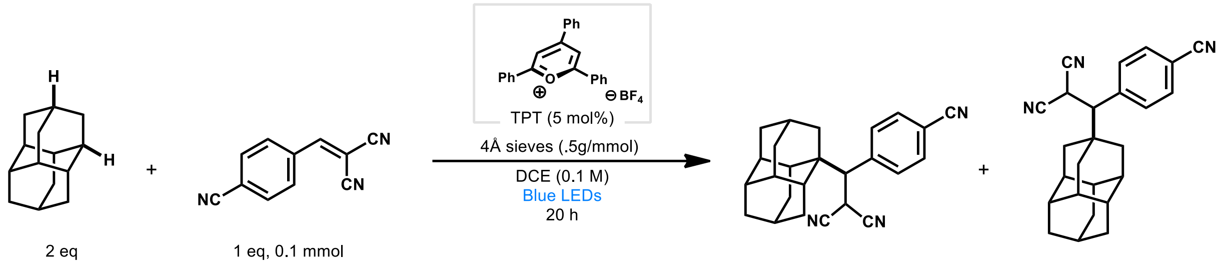

Reaction	Desiccation method	% Alkene	NMR Yield (%)	r.r. (medial:apical)
a	Solids weighed in glovebox	<6	98	5.5 : 1
b	3Å	3	65	8.3 : 1
c	4Å	4.5	81	4.4 : 1
d	5Å	4	63	3.8 : 1

Product yield determined by ^{1}H NMR using dibenzyl ether as an internal standard. Medial product: δ4.17; apical product: δ4.28.

Initial experiments conducted within a glovebox environment led to an impressive 98% product with a 5.5:1 medial:apical ratio (**Table S5**). This result substantiates our earlier suspicions that water adversely affects the reaction. While this data is pivotal for the reaction's efficiency, we sought a more accessible desiccation method than glovebox techniques. Batches of molecular sieves were dried under high vacuum at >230 °C for 24 hours prior to use. Pleasingly, 4Å sieves

provided the most workable NMR yield at 81% with minimal remaining starting material. We opted to proceed with the 4Å-sized sieves with the remaining optimization efforts.

Other organic solvents other than DCE were tested (**Table S6**), with DCM providing the best alternative with 70% yield product at a 3.3:1 ratio (a). Oxygenated solvents and nonpolar solvents did little to facilitate observable product formation (b-e). Acetonitrile, commonly used in other functionalization methods of diamondoids, fashioned a modest yield of 46% and a 5.2:1 product ratio (f). Ethyl acetate, acetone, and trifluorotoluene ($PhCF_3$) all provided low-to-moderate yields of alkylated diamantane in appreciable r.r.s. ranging from 2.6:1 to 4.0:1 (g-i).

Table S6. Survey of organic solvents.

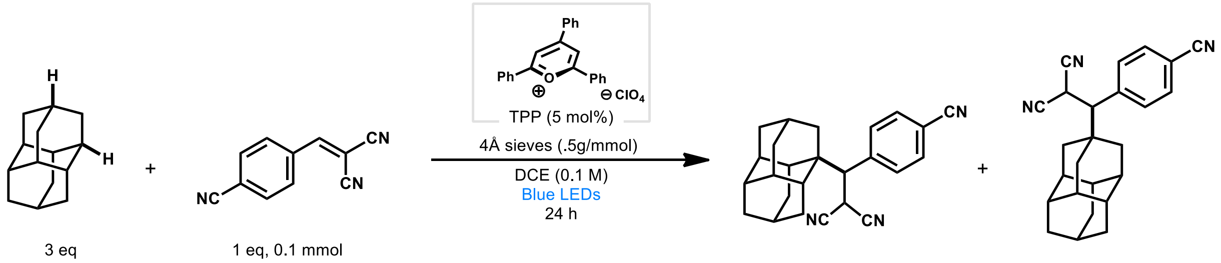

Reaction	Desiccation method	NMR Yield (%)	r.r. (medial:apical)
a	DCM	70	3.3 : 1
b	MeOH	Trace	-
c	THF	0	-
d	Pentane	0	-
e	Toluene	0	-
f	MeCN	46	5.2 : 1
g	EtOAc	5	4.0 : 1
h	Acetone	32	2.6 : 1
i	$PhCF_3$	10	3.8 : 1

Product yield determined by ^{1}H NMR using dibenzyl ether as an internal standard. Medial product: δ4.17; apical product: δ4.28.

Table S7. Catalyst screen

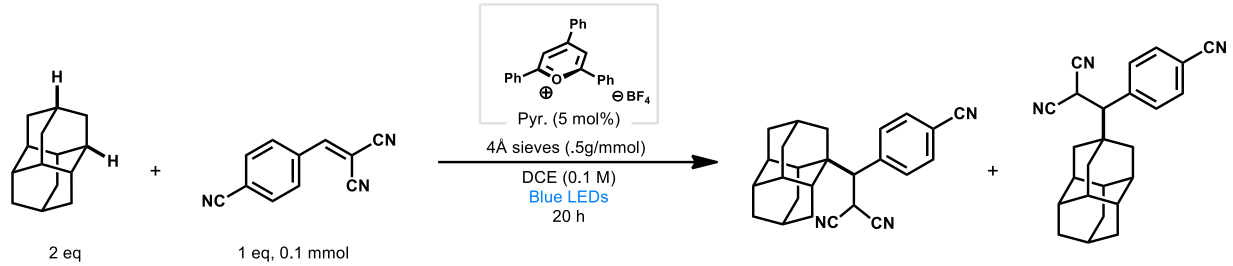

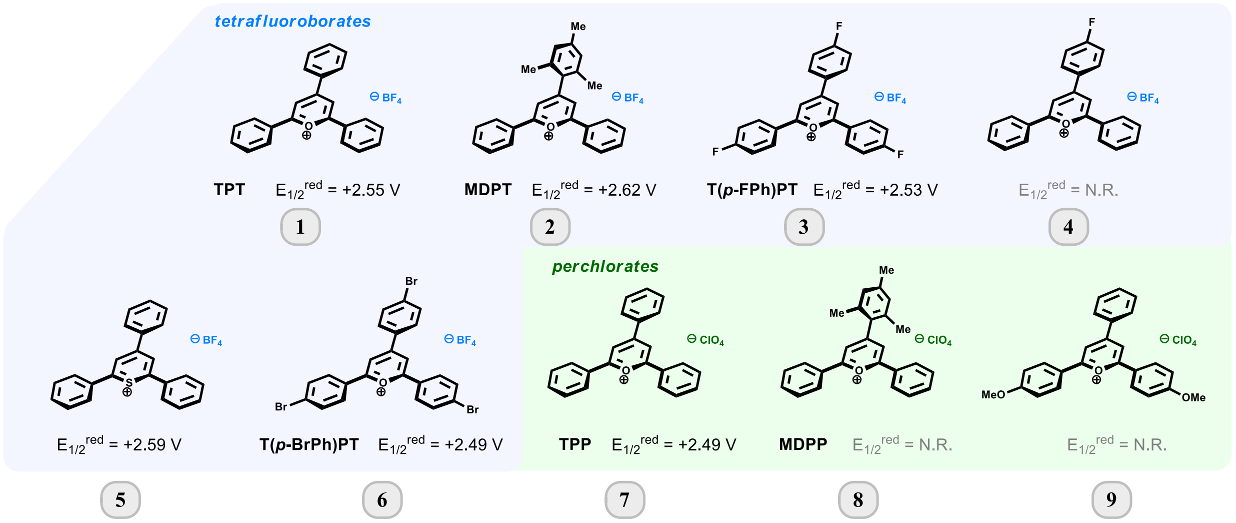

Reaction	Pyrylium	% Alkene	NMR Yield	r.r.
a	1 (TPT)	3	91	4.3 : 1
b	2	87	16	4.3 : 1
c	3*	4	46	4.1 : 1
d	4*	8	27	8.0 : 1
e	5	33	37	2.4 : 1
f	6	100	trace	-
g	7 (TPP)	4	83	3.6 : 1
h	8 *	40	14	6.0 : 1
i	9	97	0	-

Product yield determined by ^{1}H NMR using dibenzyl ether as an internal standard. Medial product: δ4.17; apical product: δ4.28.

With nearly optimized conditions in hand, we performed a photocatalyst screen. Shown in **Table S7** are the results of reactions (with activated sieves and without base) of an array of pyrylium tetrafluoroborate and perchlorate salts. TPT (pyrylium 1) provided the best efficiency of 91%, followed closely by TPP (pyrylium 7) at 83%. The perchlorate showed slightly lower reaction efficiency, but otherwise has frequently outperformed the tetrafluoroborate on several occasions. MDPT (pyrylium 2), a mesitylated pyrylium that was hypothesized to oxidize

diamantane efficiently, led to only 16% yield by NMR with significant remaining starting material. Its perchlorate variant (pyrylium 8), which is presumed to exhibit higher molar absorptivity, performed with similarly low efficiency (14%). Pyryliums 6 and 9 provided little-to-no evidence of product. From this screen, TPT and TPP were selected as the model photocatalysts for this study.

Table S8. Catalyst screen using *N*-methyl or *N*-phenyl acridinium salts

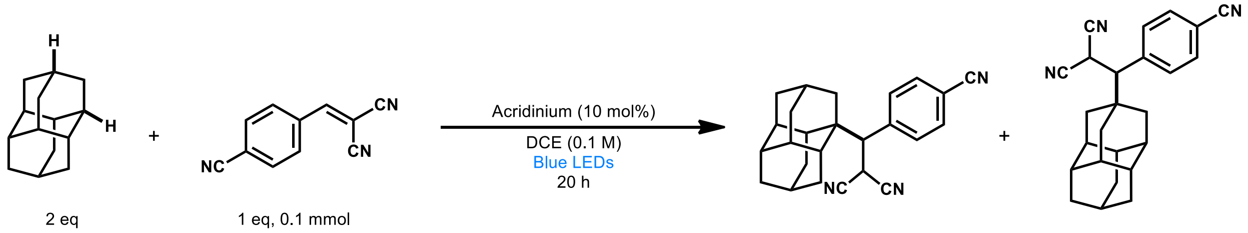

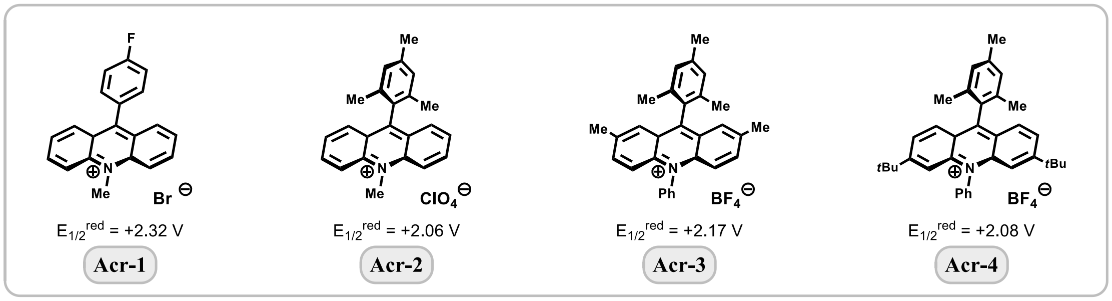

Reaction	Catalyst	% Alkene	NMR Yield	r.r.
a	Acr-1	73	13	5.5 : 1
b	Acr-2	82	Trace	-
c	Acr-3	82	2	Medial
d	Acr-4	99	0	-

Product yield determined by ^{1}H NMR using phenanthrene as an internal standard. Medial product: δ4.17; apical product: δ4.28.

We subjected various photocatalytic acridinium salts to the reaction at a 10% catalyst loading (**Table S8**). **Acr-2**, **Acr-3**, and **Acr-4** were purchased from commercial vendors, while **Acr-1** was synthesized based on a previously reported procedure by Sparr and colleagues.[4] Out of the 4 catalysts, **Acr-1** had the highest potential ($E_{1/2}^{red}$ = +2.32 V vs SCE) closely matching to that of diamantane ($E_{1/2}^{red}$ = +2.37 V), but led to a low yield of 13% over 20 hours (a). Other mesitylated acridinium catalysts provided little-to-no product formation, which was to be expected given their much lower oxidation potentials.

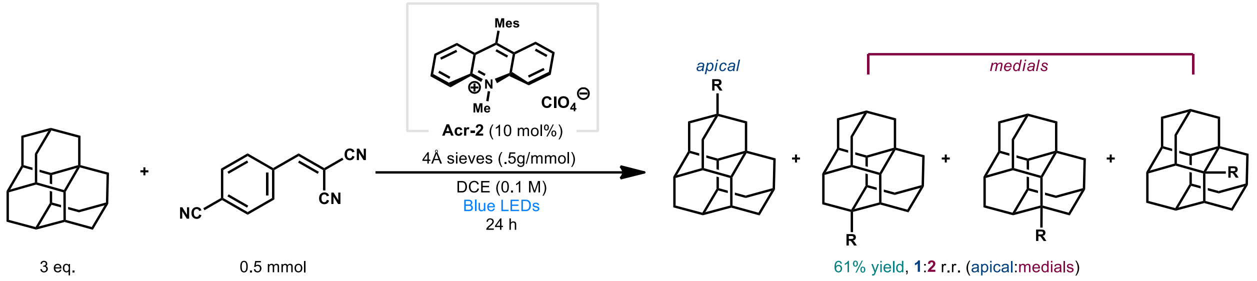

Given triamantane's lower oxidation potential (+2.18 V vs. SCE in MeCN, see **Figure S9**), we tried to couple triamantane and benzylidene using 10 mol% **Acr-2** with 4Å sieves. The alkylation reaction led to a modest 61% yield with a 1:2 ratio of apical:medial products, suggesting that the oxidizing power of acridinium catalysts would be suitable for photoredox-mediated transformations of higher diamondoids.

Table S9. Optimization of catalyst loading, concentration, and amount of sieves

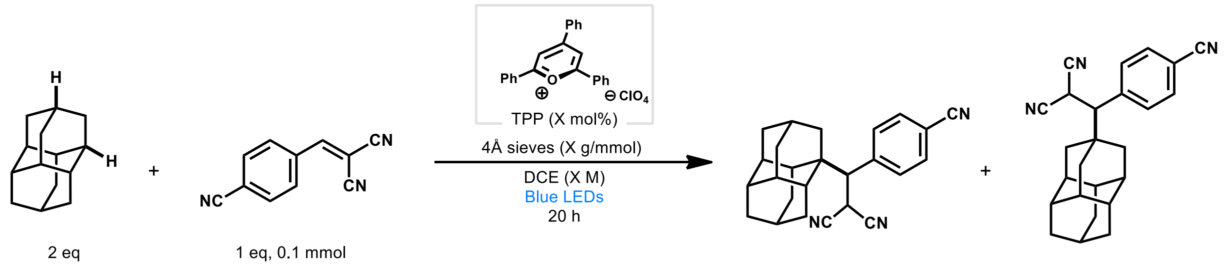

Reaction	Deviation	% Alkene	NMR Yield	r.r.
a	5 mol% TPP	17	71	3.7 : 1
b	7.5 mol% TPP	7	72	4.1 : 1
c	10 mol% TPP	5	78	3.9 : 1
d	12.5 mol% TPP	5	75	3.7 : 1
e	15 mol% TPP	5	56	4.6 : 1
f	0.2 M DCE	79	37	4.3 : 1
g	0.05 M DCE	35	74	3.4 : 1
j	0.03 M DCE	23	84	3.2 : 1
i	0.02 M DCE	21	62	3.4 : 1
j	50 mg M.S.	3	84	3.9 : 1
k	250 mg M.S.	3	78	3.9 : 1
l	325 mg M.S.	3	79	3.9 : 1
m	400 mg M.S.	3	72	4.1 : 1

Product yield determined by ^{1}H NMR using phenanthrene as an internal standard. Medial product: δ4.17; apical product: δ4.28.

Finally, we set out to fine-tune the amounts of catalyst, solvent, and molecular sieves (**Table S9**). With the initial conditions including 10 mol%, 0.1 M DCE, and 100 mg 4Å M.S (c), the product yield was recorded at 78%. Modifying the catalyst loading, either lower or higher (a-b and d-e), resulted in slightly reduced product yields, with 15 mol% catalyst causing a 22% decrease in yield. Reducing the amount of solvent slows the reaction significantly (f). The reaction seems to exhibit better efficiency under more dilute conditions (g-h), although more repetitions of these trials should be performed to formally assert this. Finally, it was found that reducing the weight of molecular sieves to 50 mg per 0.1 mmol of limiting reagent led to the best yield (j). A higher loading of sieves can lead to the stir bar getting stuck, which then decreases the final yield.

From this study, we have determined our optimized conditions: At a 10% catalyst loading, 2,4,6-triphenylpyrylium perchlorate (**TPP**) consistently provided the highest yields of **9** and was determined to be the best catalyst. Furthermore, addition of 4Å molecular sieves (at a loading of 0.5 g per mmol of the substrate) provided the most favorable water-scavenging capacity.

II. Failed substrates and catalysts

The following substrates in **Figure S2** were investigated, and the reactions led to poor yields or no desired products.

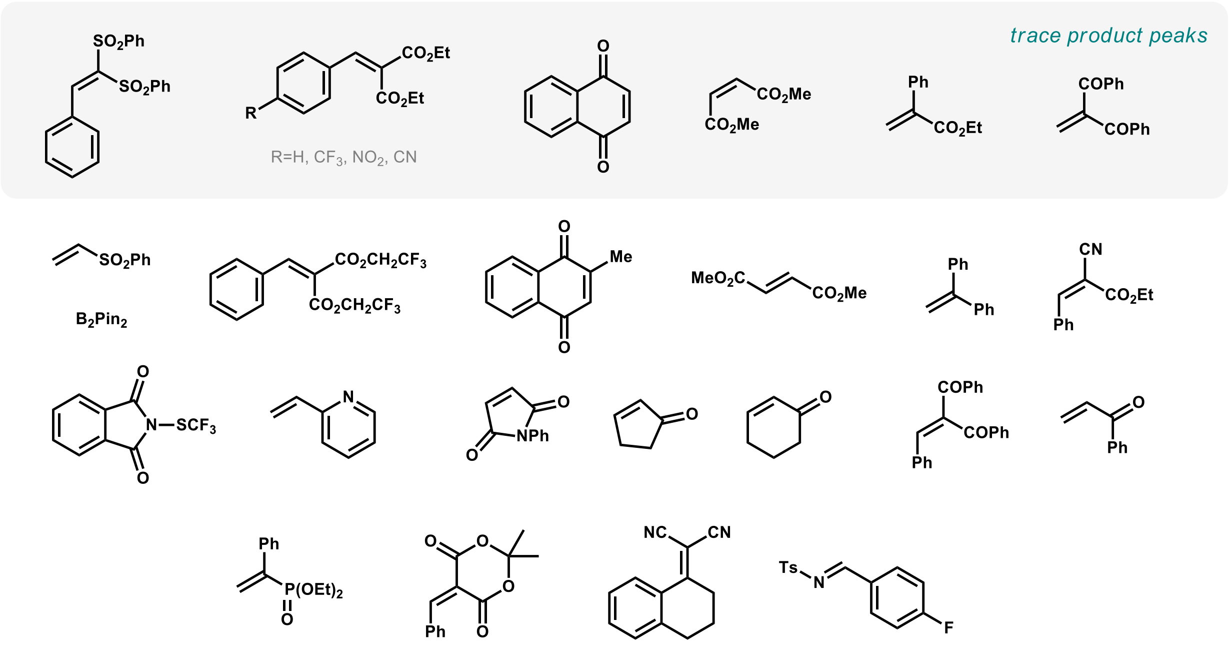

Figure S2
• Failed radical acceptors in photochemical reaction.

C. Photochemical Reactions with Diamantane

2-((Diamant-1-yl)(4-cyanophenyl)methyl)malononitrile (**9**)

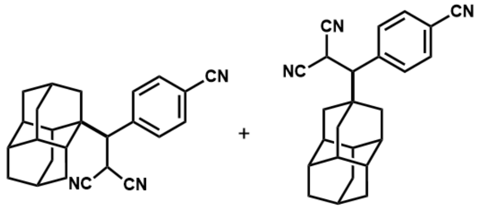

9

According to *General Procedure A*, 4-cyanobenzylidene malononitrile (0.50 mmol, 1.0 equiv), 2,4,6-triphenylpyrylium catalyst (10 mol%), diamantane (1.0 mmol, 2.0 equiv), freshly activated 4Å molecular sieves (125 mg) and DCE (5.0 mL) were reacted for 20 hours. The crude residue was purified by column chromatography on silica gel (2.5-7.5% EtOAc/Hexanes) to afford **9** (61 mg, 69% yield, 3.1:1 medial:apical). Mixture of products: **^{1}H NMR (500 MHz, $CDCl_3$)** δ 7.76 – 7.70 (m, 1H), 7.62 (d, *J* = 8.0 Hz, 2H), 7.56 – 7.52 (m, 1H), 4.28 (d, *J* = 5.1 Hz, 1H), 4.17 (d, *J* = 5.1 Hz, 1H), 4.02 (d, *J* = 5.1 Hz, 1H), 2.92 (d, *J* = 5.2 Hz, 1H), 2.22 – 2.14 (m, 2H), 2.10 – 2.03 (m, 2H), 2.03 – 1.96 (m, 3H), 1.96 – 1.92 (m, 1H), 1.91 – 1.85 (m, 2H), 1.83 – 1.77 (m, 2H), 1.79 – 1.71 (m, 6H), 1.70 – 1.68 (m, 1H), 1.68 – 1.60 (m, 3H), 1.60 – 1.55 (m, 1H), 1.53 – 1.42 (m, 2H). **IR** (neat, cm^{-1}): 2911, 2852, 2226, 1606, 1506, 1471, 1345, 1230. **HRMS** (ESI) m/z calcd for $C_{25}H_{24}N_3$ $(M\text{-}H)^-$ = 366.1965, found 366.1977.

MEDIAL (**9m**): **^{1}H NMR** (500 MHz, $CDCl_3$) δ 7.72 (d, *J* = 8.3 Hz, 1H), 7.61 (d, *J* = 8.1 Hz, 1H), 4.16 (d, *J* = 5.1 Hz, 1H), 4.01 (d, *J* = 5.1 Hz, 1H), 2.20 – 2.12 (m, 1H), 2.09 – 2.02 (m, 1H), 2.02 – 1.96 (m, 2H), 1.95 – 1.92 (m, 1H), 1.89 – 1.87 (m, 1H), 1.82 – 1.78 (m, 1H), 1.77 – 1.66 (m, 6H), 1.66 – 1.60 (m, 1H), 1.59 – 1.55 (m, 1H), 1.52 – 1.41 (m, 2H).**^{13}C NMR** (101 MHz, $CDCl_3$) δ 140.2, 132.3, 130.8, 118.2, 113.3, 112.8, 112.7, 48.9, 40.5, 38.6, 38.5, 38.3, 38.0, 37.7, 37.2, 37.1, 36.9, 36.6, 32.5, 32.4, 26.9, 25.1, 22.6.

APICAL (**9a**): **^{1}H NMR** (400 MHz, $CDCl_3$) δ 7.71 (d, *J* = 8.0 Hz, 1H), 7.53 (d, *J* = 7.9 Hz, 1H), 4.28 (d, *J* = 5.1 Hz, 1H), 2.92 (d, *J* = 5.2 Hz, 1H). 1.87 – 1.85 (m, 3H), 1.81 – 1.78 (m, 1H), 1.71-1.69 (m, 6H), 1.64-1.61 (m, 9H). **^{13}C NMR** (101 MHz, $CDCl_3$) δ 140.8, 132.5, 130.6, 118.3, 113.1, 112.9, 112.8, 57.4, 41.2, 37.5, 37.3, 36.4, 35.2, 25.3, 23.7.

2-((Diamant-1-yl)(4-trifluoromethylphenyl)methyl)malononitrile (**10**)

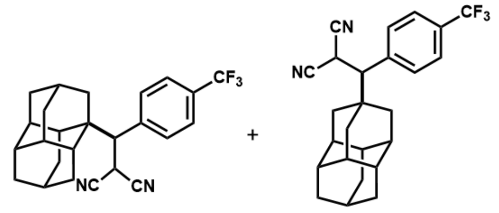

10

According to *General Procedure A*, 4-trifluoromethylbenzylidene malononitrile (0.50 mmol, 1.0 equiv), 2,4,6-triphenylpyrylium catalyst (10 mol%), diamantane (1.0 mmol, 2.0 equiv), freshly activated 4Å molecular sieves (125 mg) and DCE (5.0 mL) were reacted for 20 hours. The crude residue was purified by column chromatography on silica gel (2.5-10% EtOAc/Hexanes) to afford **10** (72 mg, 69% yield, 3.5:1 medial:apical). **^{1}H NMR** (500 MHz, $CDCl_3$) δ 7.68 (d, *J* = 8.1 Hz, 7H), 7.62 (d, *J* = 8.1 Hz, 5H), 7.54 (d, *J* = 8.0 Hz, 3H), 4.29 (d, *J* = 5.1 Hz, 1H), 4.18 (d, *J* = 5.0 Hz, 2H), 4.03 (d, *J* = 5.1 Hz, 2H), 2.93 (d, *J* = 5.1 Hz, 1H), 2.19 (dd, *J* = 13.7, 2.2 Hz, 2H), 2.06 (dd, *J* = 12.3, 2.3 Hz, 2H), 2.02 – 1.97 (m, 3H), 1.95 – 1.92 (m, 1H), 1.92 – 1.85 (m, 3H), 1.82 – 1.77 (m, 1H), 1.77 – 1.69 (m, 5H), 1.69 – 1.59 (m, 5H), 1.58 – 1.53 (m, 1H), 1.51 – 1.42 (m, 2H), 1.35 – 1.23 (m, 3H). **^{13}C NMR** (126 MHz, Chloroform-*d*) δ 139.5, 139.0, 131.0, 130.8, 130.6, 125.7, 125.7 (q, *J* = 3.8 Hz), 124.0 (d, *J* = 272.2 Hz), 113.6, 113.2, 113.1, 113.0, 57.3, 48.8, 41.3, 40.5, 38.8, 38.7, 38.5, 38.2, 37.7, 37.6, 37.4, 37.4, 37.1, 36.7, 36.5, 35.1, 32.7, 32.6, 29.8, 27.1, 25.4, 25.3, 23.8, 22.9. **^{19}F NMR** (471 MHz, $CDCl_3$) δ -62.74, -62.75. **IR** (neat, cm^{-1}): 2916, 2853, 1620, 1458, 1327, 1187. **HRMS** (ESI) m/z calc'd for $C_{25}H_{24}F_3N_2$ $(M-H)^-$ = 409.1886, found 409.1900.

2-((Diamant-1-yl)(4-nitrophenyl)methyl)malononitrile (**11**)

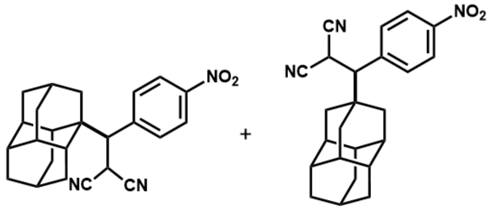

11

According to *General Procedure A,* 4-nitrobenzylidene malononitrile (0.50 mmol, 1.0 equiv), 2,4,6-triphenylpyrylium catalyst (10 mol%), diamantane (1.0 mmol, 2.0 equiv), freshly activated 4Å molecular sieves (125 mg) and DCE (5.0 mL) were reacted for 24 hours. The crude residue was purified by column chromatography on silica gel (10% EtOAc/Hexanes) to afford **11** (62 mg, 63% yield, 3.5:1 medial:apical). **^{1}H NMR** (500 MHz, $CDCl_3$) δ 8.13 (d, *J* = 8.0 Hz, 3H), 7.53 (d, *J* = 8.3 Hz, 2H), 7.49 – 7.42 (m, 1H), 4.15 (d, *J* = 5.1 Hz, 1H), 4.04 (d, *J* = 5.0 Hz, 1H), 3.94 (d, *J* = 5.0 Hz, 1H), 2.84 (d, *J* = 5.0 Hz, 1H), 2.03 (d, 1H), 1.97 – 1.77 (m, 6H), 1.74 (d, *J* = 13.6 Hz, 2H), 1.68 – 1.52 (m, 10H), 1.52 – 1.26 (m, 18H). **^{13}C NMR** (101 MHz, CDCl3) δ. **^{13}C NMR** (126 MHz, $CDCl_3$) δ 142.3, 123.9, 123.8, 113.4, 112.8, 57.2, 48.8, 40.7, 38.8, 38.6, 38.4, 38.2, 37.9, 37.3, 37.3, 37.0, 36.8, 36.5, 32.7, 32.6, 29.8, 27.0, 25.4, 25.3, 23.7, 22.8. **IR** (neat,

cm^{-1}): 2911, 2851, 2254, 1602, 1512, 1459, 1350, 1260. **HRMS** (ESI) m/z calcd for $C_{24}H_{24}N_3O_2$ (M-H)$^-$ = 386.1863, found 386.1877.

2-((Diamant-1-yl)(4-fluorophenyl)methyl)malononitrile (**12**)

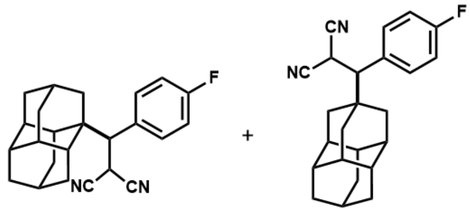

12

According to *General Procedure A*, 4-fluorobenzylidene malononitrile (0.50 mmol, 1.0 equiv), 2,4,6-triphenylpyrylium catalyst (10 mol%), diamantane (1.0 mmol, 2.0 equiv), freshly activated 4Å molecular sieves (125 mg) and DCE (5.0 mL) were reacted for 24 hours. The crude residue was purified by column chromatography on silica gel (5-10% EtOAc/Hexanes) to afford **12** (71.1 mg, 79% yield, 4.5:1 medial:apical). **^{1}H NMR** (400 MHz, $CDCl_3$) δ 7.47 (dd, *J* = 8.3, 5.2 Hz, 2H), 7.42 – 7.35 (m, 1H), 7.11 (t, *J* = 8.5 Hz, 2H), 4.25 (d, *J* = 5.1 Hz, 1H), 4.14 (d, *J* = 5.1 Hz, 1H), 3.95 (d, *J* = 5.1 Hz, 1H), 2.85 (d, *J* = 5.1 Hz, 1H), 2.17 (d, *J* = 13.4 Hz, 1H), 2.09 – 2.01 (m, 1H), 2.01 – 1.95 (m, 2H), 1.95 – 1.83 (m, 3H), 1.83 – 1.76 (m, 1H), 1.76 – 1.69 (m, 7H), 1.69 – 1.58 (m, 4H), 1.51 – 1.43 (m, 1H), 1.43 – 1.40 (m, 1H), 1.37 – 1.30 (m, 1H), 1.22 (t, *J* = 7.0 Hz, 1H). **^{13}C NMR** (101 MHz, $CDCl_3$) δ 162.7 (d, *J* = 248.3 Hz), 131.7 (d, *J* = 8.7 Hz), 130.5 (d, *J* = 3.7 Hz), 115.7 (d, *J* = 21.3 Hz), 113.4 (d, *J* = 43.5 Hz), 48.2, 41.1, 40.2, 38.6, 38.6, 38.4, 38.1, 37.5, 37.5, 37.3, 37.0, 36.5, 36.4, 32.6, 32.4. **IR** (neat, cm^{-1}): 2909, 2851, 1606, 1512, 1460, 1339, 1231. **^{19}F NMR** (282 MHz, $CDCl_3$) δ -113.08, -113.15. **HRMS** (ESI) m/z calcd for $C_{24}H_{24}N_2F$ (M-H)$^-$ = 359.1929, found 359.1930.

2-((Diamant-1-yl)(4-chlorophenyl)methyl)malononitrile (**13**)

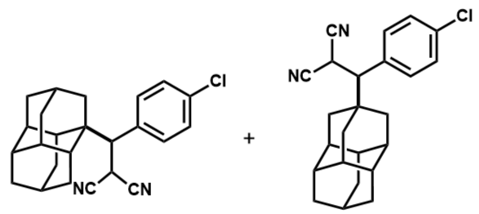

13

According to *General Procedure A,* 4-chlorobenzylidene malononitrile (0.50 mmol, 1.0 equiv), 2,4,6-triphenylpyrylium catalyst (10 mol%), diamantane (1.0 mmol, 2.0 equiv), freshly activated 4Å molecular sieves (125 mg) and 1,2-dichloroethane (5.0 mL) were reacted for 24 hours. The crude residue was purified by column chromatography on silica gel (5% -10% EtOAc/Hexanes) to afford **13** (130.1 mg, 69% yield, 3.6:1 medial:apical). **IR** (neat, cm^{-1}): 2906, 1494, 1096, 832, 748. **^{1}H NMR** (400 MHz, CDCl3) δ 7.43 – 7.41 (d, *J* = 8.6 Hz, 2H), 7.39 – 7.37 (d, *J* = 8.8 Hz, 2H), 7.34 – 7.32 (d, 1H), 4.25 – 4.24 (d, *J* = 5.3 Hz, 1H), 4.14 – 4.13 (d, *J* = 5.1 Hz, 1H), 3.93 – 3.92 (d, *J* = 5.1 Hz, 1H), 2.84 – 2.82 (d, *J* = 5.2 Hz, 1H), 2.17 –

2.13 (m, 1H), 2.05 – 2.01 (m, 1H), 2.01 – 1.97 (m, 2H), 1.97 – 1.91 (m, 1H), 1.91 – 1.86 (m, 2H), 1.80 – 1.76 (m, 2H), 1.77 – 1.62 (m, 7H), 1.62 – 1.59 (m, 3H), 1.48 – 1.41 (m, 2H). **^{13}C NMR** (101 MHz, $CDCl_3$) δ 134.7, 134.6, 133.9, 133.2, 131.3, 128.9, 128.9, 113.6, 113.3, 113.2, 113.1, 56.8, 48.3, 41.1, 40.3, 40.2, 38.6, 38.6, 38.4, 38.0, 37.6, 37.5, 37.3, 37.2, 37.1, 37.0, 36.6, 36.4, 36.0, 34.9, 32.6, 32.4, 29.7, 27.0, 25.3, 25.2, 23.9, 23.0. **HRMS** (ESI) m/z calcd for $C_{24}H_{25}N_2Cl$ $(M)^+$ = 376.1704, found 376.1700.

2-((Diamant-1-yl)(4-bromophenyl)methyl)malononitrile (**14**)

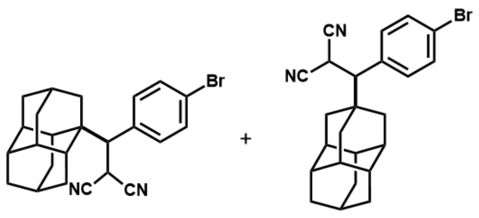

14

According to *General Procedure A*, 4-bromobenzylidene malononitrile (0.50 mmol, 1.0 equiv), 2,4,6-triphenylpyrylium catalyst (10 mol%), diamantane (1.0 mmol, 2.0 equiv), freshly activated 4Å molecular sieves (125 mg) and DCE (5.0 mL) were reacted for 24 hours. The crude residue was purified by column chromatography on silica gel (5-10% EtOAc/Hexanes) to afford **14** (84 mg, 80% yield, 2.9:1 medial:apical). **^{1}H NMR** (500 MHz, $CDCl_3$) δ 7.81 – 7.76 (m, 2H), 7.75 – 7.66 (m, 3H), 7.58 – 7.47 (m, 3H), 7.36 (d, *J* = 8.2 Hz, 2H), 4.25 (d, *J* = 5.2 Hz, 1H), 4.14 (d, *J* = 5.1 Hz, 1H), 3.93 (d, *J* = 5.1 Hz, 1H), 2.83 (d, *J* = 5.2 Hz, 1H), 2.21 – 2.12 (m, 1H), 2.07 – 2.01 (m, 1H), 2.01 – 1.94 (m, 2H), 1.93 – 1.90 (m, 1H), 1.90 – 1.83 (m, 2H), 1.80 – 1.76 (m, 2H), 1.76 – 1.69 (m, 7H), 1.69 – 1.58 (m, 3H), 1.51 – 1.39 (m, 2H). **^{13}C NMR** (126 MHz, $CDCl_3$) δ 133.9, 132.0, 131.8, 123.0, 113.7, 113.2, 48.6, 40.4, 38.8, 38.7, 38.5, 38.2, 37.7, 37.4, 37.2, 36.7, 32.7, 32.6, 29.9, 27.1, 25.3, 23.1. **HRMS** (ESI) m/z calcd for $C_{24}H_{24}BrN_2$ $(M-H)^-$ = 419.1117, found 419.1132.

2-((Diamant-1-yl)(phenyl)methyl)malononitrile (**15**)

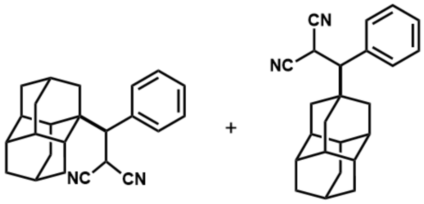

15

According to *General Procedure A*, benzylidene malononitrile (0.50 mmol, 1.0 equiv), 2,4,6-triphenylpyrylium catalyst (10 mol%), diamantane (1.0 mmol, 2.0 equiv), freshly activated 4Å molecular sieves (125 mg) and DCE (5.0 mL) were reacted for 20 hours. The crude residue was purified by column chromatography on silica gel (5% EtOAc/Hexanes) to

afford **15** (49 mg, 58% yield, 3.1:1 medial:apical). **^{1}H NMR** (400 MHz, $CDCl_3$) δ 7.51 – 7.46 (m, 2H), 7.44 – 7.38 (m, 4H), 4.27 (d, *J* = 5.4 Hz, 1H), 4.17 (d, *J* = 5.3 Hz, 1H), 3.97 (d, *J* = 5.3 Hz, 1H), 2.87 (d, *J* = 5.4 Hz, 1H), 2.32 – 2.19 (m, 1H), 2.16 – 2.01 (m, 2H), 2.01 – 1.84 (m, 5H), 1.83 – 1.54 (m, 16H), 1.52 – 1.33 (m, 3H). **^{13}C NMR** (126 MHz, $CDCl_3$) δ 135.6, 134.9, 130.2, 128.7, 128.7, 128.7, 128.6, 113.5, 57.5, 49.0, 41.3, 40.4, 38.8, 38.8, 38.6, 38.3, 37.7, 37.7, 37.5, 37.5, 37.5, 36.8, 36.6, 35.0, 32.8, 32.6, 29.8, 27.1, 25.5, 25.4, 24.1, 23.3. **IR** (neat, cm^{-1}): 2904, 2871, 1500, 1472, 1352, 1218, 1185. **HRMS** (ESI) m/z calcd for $C_{24}H_{25}N_2$ $(M-H)^-$ = 341.2012, found 341.2022.

Diethyl 2-(diamant-1-yl)malonate (**16**)

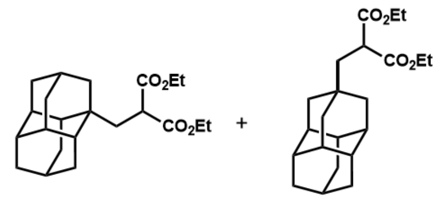

16

According to *General Procedure A*, diethyl methylenemalonate (0.5 mmol, 1.0 equiv), 2,4,6-triphenylpyrylium catalyst (10 mol %), diamantane (1.0 mmol, 2.0 equiv), freshly activated 4Å molecular sieves (125 mg), and 1,2-dichloroethane (5 mL) for 36 hours. The crude residue was purified by column chromatography on silica gel (1-5% EtOAc/Hexanes) to afford **16** (91.7 mg, 49.3% yield, 4.8:1 medial:apical). **^{1}H NMR** (400 MHz, $CDCl_3$) δ 4.2 (q, *J* = 7.1 Hz, 5H), 3.4 (t, *J* = 6.3 Hz, 1H), 3.4 (t, *J* = 6.0 Hz, 1H), 2.2 (d, *J* = 6.1 Hz, 2H), 2.1 – 2.0 (m, 2H), 1.9 – 1.8 (m, 1H), 1.9 – 1.7 (m, 4H), 1.7 – 1.7 (m, 2H), 1.6 (d, *J* = 3.0 Hz, 6H), 1.6 (s, 1H), 1.5 – 1.4 (m, 2H), 1.4 – 1.4 (m, 2H), 1.4 – 1.3 (m, 3H), 1.3 (t, *J* = 7.1 Hz, 7H). **^{13}C NMR** (101 MHz, $CDCl_3$) δ 170.8, 170.6, 61.5, 47.4, 47.0, 42.7, 41.7, 41.6, 40.0, 39.1, 38.6, 38.1, 37.9, 37.9, 37.8, 37.0, 36.4, 36.3, 32.7, 30.4, 27.6, 25.8, 14.2. **IR** (neat, cm^{-1}): 2979, 2868, 1750, 1730, 1461, 1279, 1223, 1175, 1140. **HRMS** (ESI) m/z calcd for $C_{22}H_{33}O_4$ $(M+H)^+$ = 361.2379, found 361.237.

2-((Diamant-1-yl)-1,1-bis(phenylsulfonyl)ethane (**17**)

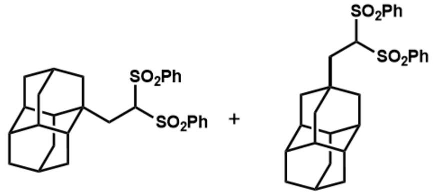

17

According to *General Procedure A*, 1,1-bis(phenylsulfonyl)ethylene (0.50 mmol, 1.0 equiv), 2,4,6-triphenylpyrylium catalyst (10 mol%), diamantane (1.0 mmol, 2.0 equiv), freshly activated 4Å molecular sieves (125 mg) and DCE (5.0 mL) were reacted for 24 hours. The

crude residue was purified by column chromatography on silica gel (2.5-10% EtOAc/Hexanes) to afford **17** (72 mg, 59% yield, 3.3:1 medial:apical). **^{1}H NMR** (400 MHz, $CDCl_3$) δ 8.02 – 7.92 (m, 5H), 7.77 – 7.67 (m, 3H), 7.64 – 7.51 (m, 5H), 4.65 (t, *J* = 4.2 Hz, 1H), 4.54 (t, *J* = 4.0 Hz, 1H), 2.42 (d, *J* = 4.2 Hz, 2H), 2.21 – 2.12 (m, 2H), 2.09 (d, *J* = 4.0 Hz, 1H), 1.97 – 1.91 (m, 1H), 1.87 – 1.77 (m, 4H), 1.75 – 1.60 (m, 10H), 1.60 – 1.52 (m, 3H), 1.52 – 1.36 (m, 6H). **^{13}C NMR** (101 MHz, $CDCl_3$) δ 138.3, 134.4, 134.4, 130.0, 129.8, 129.0, 129.0, 80.9, 42.7, 41.3, 40.4, 38.8, 38.7, 37.7, 37.7, 37.5, 37.5, 36.6, 36.6, 32.9, 31.7, 27.4, 25.5. **IR** (neat, cm^{-1}): 2872, 1583, 1446, 1309, 1144. **HRMS** (ESI) m/z calcd for $C_{28}H_{33}O_4S_2$ $(M+H)^+$ =497.1815, found 497.1805.

1-Azidodiamantane (**18**)

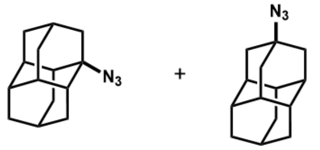

18

According to *General Procedure A*, 4-(trifluoromethyl)benzenesulfonyl azide (0.50 mmol, 1.0 equiv), 2,4,6-triphenylpyrylium catalyst (10 mol%), diamantane (1.0 mmol, 2.0 equiv), freshly activated 4Å molecular sieves (125 mg) and DCE (5.0 mL) were reacted for 20 hours. The crude residue was purified by column chromatography on silica gel (2.5-10% EtOAc/Hexanes) to afford **18** (25 mg, 44% yield, 3.1:1 medial:apical). NMR data are in accordance with literature values.[5] **^{1}H NMR** (500 MHz, $CDCl_3$) MEDIAL: δ 2.16 – 2.07 (m, 3H), 2.04 – 1.95 (m, 2H), 1.88 – 1.82 (m, 2H), 1.82 – 1.73 (m, 3H), 1.73 – 1.64 (m, 7H), 1.52 – 1.46 (m, 2H). APICAL: δ 2.05 – 1.93 (m, 3H), 1.85 – 1.80 (m, 2H), 1.77 (dd, *J* = 11.3, 2.8 Hz, 14H). **^{13}C NMR** (101 MHz, $CDCl_3$) MEDIAL: δ 42.0, 40.4, 39.3, 38.0, 37.2, 36.7, 33.2, 29.9, 29.2, 25.1. APICAL: δ 42.0, 38.9, 37.1, 36.2. **IR** (neat, cm^{-1}): 2913, 2850, 2100, 2082, 1602, 1459, 1340, 1257. Sample was subjected to **GC/MS**: m/z calcd for $C_{14}H_{19}N_3$ $(M)^+$ = 229.1577, found 229.1571.

1-Diamantylcyanide (**19**)

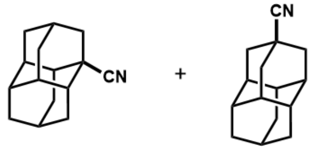

19

According to a modified *General Procedure A*, *p*-toluenesulfonyl cyanide (0.1 mmol, 1.0 equiv), 2,4,6-triphenylpyrylium catalyst (10 mol %), diamantane (0.2 mmol, 2.0 equiv), and 1,2-dichloroethane (1 mL) were reacted for 24 hours in 3 separate vials. The crude residue was combined and purified by column chromatography on silica gel (100% Pentane) to

afford **19** (22.4 mg, 35% yield, 4:1 medial:apical). NMR data are in accordance with literature values.[6] **^{1}H NMR** (400 MHz, $CDCl_3$) δ 2.23 – 2.15 (dt, J = 13.4, 3.1 Hz, 2H), 2.04 – 2.02 (d, J = 3.2 Hz, 2H), 2.02 – 2.00 (d, J = 2.9 Hz, 1H), 2.00 – 1.96 (d, J = 3.1 Hz, 2H), 1.96 – 1.92 (p, J = 3.2 Hz, 1H), 1.91 – 1.84 (dp, J = 6.0, 2.9 Hz, 4H), 1.84 – 1.79 (m, 0.5H), 1.78 – 1.67 (m, 12H). **^{13}C NMR** (100.7 MHz, $CDCl_3$) MEDIAL: δ 124.6, 41.4, 39.0, 38.1, 37.6, 37.0, 36.5, 36.3, 35.0, 25.5, 24.9. APICAL: δ 125.6, 40.8, 37.6, 36.4, 36.1, 28.8, 25.3. **IR** (neat, cm^{-1}): 2907, 2882, 2851, 2226, 1441, 1342, 1049, 804 cm^{-1}. **HRMS** (ESI) m/z calcd for $C_{15}H_{20}N$ $(M+H)^+$ = 214.1596, found 214.1589.

((Triamant-1-yl)(4-bromophenyl)methyl)malononitrile (**20**)

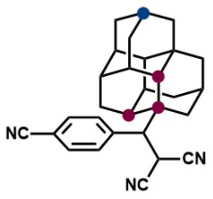

20

According to *General Procedure A*, 4-cyanobenzylidene malononitrile (0.50 mmol, 1.0 equiv), 2,4,6-triphenylpyrylium catalyst (10 mol%), triamantane (1.0 mmol, 2.0 equiv), freshly activated 4Å molecular sieves (125 mg) and DCE (5.0 mL) were reacted for 24 hours. The crude residue was purified by column chromatography on silica gel (5-10% EtOAc/Hexanes) to afford all 4 isomers of **20** (167.6 mg, 80%, 1:2.6 apical:medials). **^{1}H NMR** (500 MHz, $CDCl_3$) δ 7.81 – 7.76 (m, 2H), 7.75 – 7.66 (m, 3H), 7.58 – 7.47 (m, 3H), 7.36 (d, *J* = 8.2 Hz, 2H), 4.25 (d, *J* = 5.2 Hz, 1H), 4.14 (d, *J* = 5.1 Hz, 1H), 3.93 (d, *J* = 5.1 Hz, 1H), 2.83 (d, *J* = 5.2 Hz, 1H), 2.21 – 2.12 (m, 1H), 2.07 – 2.01 (m, 1H), 2.01 – 1.94 (m, 2H), 1.93 – 1.90 (m, 1H), 1.90 – 1.83 (m, 2H), 1.80 – 1.76 (m, 2H), 1.76 – 1.69 (m, 7H), 1.69 – 1.58 (m, 3H), 1.51 – 1.39 (m, 2H). **IR** (neat, cm^{-1}): 2892, 2847, 2229, 1729, 1607, 1458, 1339. **HRMS** (ESI) m/z calcd for $C_{29}H_{28}N_3$ $(M-H)^-$ = 418.2289, found 418.2289.

Isomer A (apical): **^{1}H NMR** (500 MHz, $CDCl_3$) δ 7.71 (d, *J* = 8.1 Hz, 2H), 7.52 (d, *J* = 7.9 Hz 2H), 4.28 (d, *J* = 5.2 Hz, 1H), 2.88 (d, *J* = 5.2 Hz, 1H), 1.89 – 1.83 (m, 1H), 1.83 – 1.78 (m, 2H), 1.75 – 1.52 (m, 14H), 1.36 (s, 2H), 1.29 – 1.25 (m, 2H), 1.16 (s, 2H). **^{13}C NMR** (101 MHz, $CDCl_3$) δ 140.7, 132.5, 130.6, 118.3, 113.1, 112.9, 112.8, 57.3, 48.0, 45.7, 45.0, 40.9, 40.9, 38.1, 37.8, 37.8, 37.6, 37.4, 37.4, 36.8, 34.6, 34.1, 34.0, 27.5, 23.6.

Isomer B: **^{1}H NMR** (500 MHz, $CDCl_3$) δ 7.72 (d, 2H), 7.63 (d, *J* = 8.1 Hz, 2H), 7.56 (d, *J* = 7.8 Hz, 1H), 4.18 (d, *J* = 4.6 Hz, 1H), 4.03 (d, *J* = 4.7 Hz, 1H), 2.23 – 2.08 (m, 2H), 2.08 – 1.97 (m, 3H), 1.95 – 1.75 (m, 6H), 1.75 – 1.67 (m, 5H), 1.67 – 1.59 (m, 2H), 1.51 – 1.44 (m, 2H), 1.42 – 1.25 (m, 5H), 1.25 – 1.18 (m, 1H), 1.18 – 1.02 (m, 1H).

Isomer C: **^{1}H NMR** (500 MHz, $CDCl_3$) δ 7.71 (d, *J* = 8.0 Hz, 2H), 7.59 (d, *J* = 8.0 Hz, 2H), 4.10 (d, *J* = 5.4 Hz, 1H), 4.01 (d, *J* = 5.4 Hz, 1H), 2.18 – 2.12 (m, 1H), 2.08 – 2.01 (m, 2H), 2.01 – 1.96 (m, 1H), 1.95 – 1.90 (m, 1H), 1.89 – 1.83 (m, 1H), 1.83 – 1.79 (m, 1H), 1.78 – 1.68 (m, 4H), 1.67 – 1.63 (m, 1H), 1.62 – 1.56 (m, 2H), 1.56 – 1.54 (m, 1H), 1.52 – 1.48 (m, 1H), 1.48 – 1.38 (m, 4H), 1.38 – 1.31 (m, 2H), 1.20 – 1.16 (m, 1H). **^{13}C NMR** (101 MHz, $CDCl_3$) δ 140.5, 132.3, 130.8, 118.2, 113.4, 112.8, 112.7, 48.2, 47.4, 46.1, 45.3, 44.5, 41.5, 38.6, 37.6, 37.5, 37.2, 37.0, 36.7, 35.7, 34.5, 34.4, 33.2, 32.3, 27.6, 27.0, 22.5.

Isomer D: **^{1}H NMR** (400 MHz, $CDCl_3$) δ 7.78 – 7.68 (m, 1H), 7.56 (d, *J* = 8.0 Hz, 1H), 4.18 (d, *J* = 5.3 Hz, 1H), 4.03 (d, *J* = 5.2 Hz, 1H), 2.23 – 2.16 (m, 1H), 2.14 – 2.07 (m, 1H), 2.05 – 1.97 (m, 2H), 1.93 – 1.87 (m, 1H), 1.86 – 1.77 (m, 4H), 1.77 – 1.60 (m, 8H), 1.60 – 1.51 (m, 1H), 1.52 – 1.42 (m, 2H), 1.31 – 1.25 (m, 1H), 1.25 – 1.11 (m, 2H), 1.10 – 0.99 (m, 1H), 1.00 – 0.94 (m, 1H). **^{13}C NMR** (101 MHz, $CDCl_3$) δ 140.4, 132.4, 130.8, 118.3, 113.5, 112.9, 112.8, 48.4, 47.1, 46.2, 45.1, 44.6, 41.6, 39.0, 37.5, 37.5, 37.3, 37.1, 36.8, 36.2, 34.6, 34.2, 33.4, 32.1, 27.8, 27.0, 23.0.

Separation of the four regioisomers (1 apical, 3 medial) can be accomplished through a series of runs on preparative thin-layer chromatography (prep TLC) plates. The preparative TLC chamber was equilibrated for 10 minutes with 15% EtOAc/Hexanes. The prep plate, initially loaded with the reaction mixture at the baseline, was run in the chamber for up to an hour. This process was repeated 3-5 times until there was sufficient separation of 4 regioisomers on the plate (visualized by UV). Bands corresponding to a single product were carefully transferred from the plate to a filter funnel, which was pre-packed lightly with celite. Elution of the product through the funnel was done using 50% EtOAc/Hexanes, and the mixture was concentrated in vacuo, yielding a single pure triamantane regioisomer.

[121]-((tetramant-1-yl)(4-cyanophenyl)methyl)malononitrile (**21**)

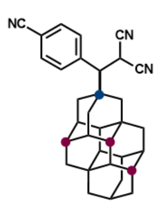

21

According to *General Procedure A*, 4-cyanobenzylidene malononitrile (0.10 mmol, 1.0 equiv), 2,4,6-triphenylpyrylium catalyst (10 mol%), [121] tetramantane (0.2 mmol, 2.0 equiv), freshly activated 4Å molecular sieves (50 mg) and DCE (2.0 mL) were reacted for 24 hours. The crude residue was purified by column chromatography on silica gel (5.0-10% EtOAc/Hexanes) to afford **21** (40.3 mg, 85% yield, 1:1.3 apical:medials). **^{1}H NMR** (400 MHz, $CDCl_3$) δ 7.73 (d, J = 8.53 Hz, 4H), 7.61

(d, J = 8.04 Hz, 2H), 7.54 (d, J = 7.17 Hz, 2H), 4.29 (d, J = 5.11 Hz, 1H), 4.18 (d, J = 5.25 Hz, 1H), 4.02 (d, J = 5.33 Hz, 1H), 2.91 (d, J = 5.13 Hz, 1H), 2.17 (d, J = 13.37 Hz, 1H), 2.04 (d, J = 14.40 Hz, 3H), 1.92 – 1.86 (m, 2H), 1.84 – 1.80 (m, 2H), 1.78 – 1.68 (m, 12H), 1.68 – 1.64 (m, 4H), 1.64 – 1.58 (m, 4H), 1.58 – 1.53 (m, 16H), 1.53 – 1.49 (m, 2H), 1.49 – 1.45 (m, 2H), 1.46 – 1.42 (m, 2H), 1.42 – 1.35 (m, 4H). **^{13}C NMR** (126 MHz, CDCl3) δ140.73, 140.52, 132.52, 132.43, 130.91, 118.32, 113.39, 113.08, 112.98, 112.91, 112.87, 112.78, 57.47, 48.47, 48.33, 48.00, 47.22, 46.68, 46.53, 46.48, 46.32, 45.60, 45.12, 45.03, 44.84, 44.56, 44.13, 44.10, 40.85, 40.48, 38.92, 38.65, 38.17, 38.07, 37.80, 37.64, 37.44, 37.01, 36.70, 36.34, 35.88, 32.47, 32.42, 32.35, 31.98, 31.73, 31.11, 30.79, 29.85, 27.84, 27.55, 27.08. **HRMS** (ESI) m/z calcd for $C_{33}H_{33}N_3$ $(M\text{-}H)^-$ = 470.2596, found 470.2604.

[1(2)3]-((tetramant-1-yl)(4-cyanophenyl)methyl)malononitrile (**22**)

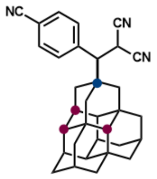

22

According to *General Procedure A*, 4-cyanobenzylidene malononitrile (0.10 mmol, 1.0 equiv), 2,4,6-triphenylpyrylium catalyst (10 mol%), [1(2)3] tetramantane (0.2 mmol, 2.0 equiv), freshly activated 4Å molecular sieves (50 mg) and DCE (2.0 mL) were reacted for 24 hours. The crude residue was purified by column chromatography on silica gel (5.0-10% EtOAc/Hexanes) to afford **22** (18.6 mg, 39.4% yield, 2.6:1apical:medials). **^{1}H NMR** (500 MHz, CDCl3) δ 7.73 (d, 4H), 7.54 – 7.49 (m, 4H), 4.26 (d, J = 5.13 Hz, 1H), 4.11 (d, J = 5.64 Hz, 1H), 4.00 (d, J = 5.62 Hz, 1H), 2.85 (d, J = 5.09 Hz, 1H), 2.17 – 2.05 (m, 2H), 2.00 – 1.93 (m, 3H), 1.92 – 1.86 (m, 6H), 1.86 – 1.81 (m, 2H), 1.75 (m, 2H), 1.74 – 1.69 (m, 6H), 1.69 – 1.63 (m, 12H), 1.62 – 1.58 (m, 12H), 1.50 – 1.38 (m, 9H). **^{13}C NMR** (101 MHz, $CDCl_3$) δ 140.6, 132.5, 132.4, 130.8, 118.3, 113.1, 113.0, 112.7, 57.4, 55.0, 54.7, 47.9 (d, *J* = 2.2 Hz), 45.7, 45.3, 45.2, 45.0, 44.9, 44.8, 44.5, 44.4, 44.4, 44.4, 44.2, 44.1, 43.6, 41.7, 40.7, 38.3, 37.7, 37.6, 37.6, 37.4, 34.3, 34.1, 33.8, 33.5, 32.8, 32.4, 32.4, 29.9, 28.5, 28.0, 27.8, 27.5, 23.6, 22.8. **HRMS** (ESI) m/z calcd for $C_{33}H_{33}N_3$ $(M\text{-}H)^-$ = 470.2596, found 470.2600.

D. Assignment of Regioisomers by 1D-nOe and XRD

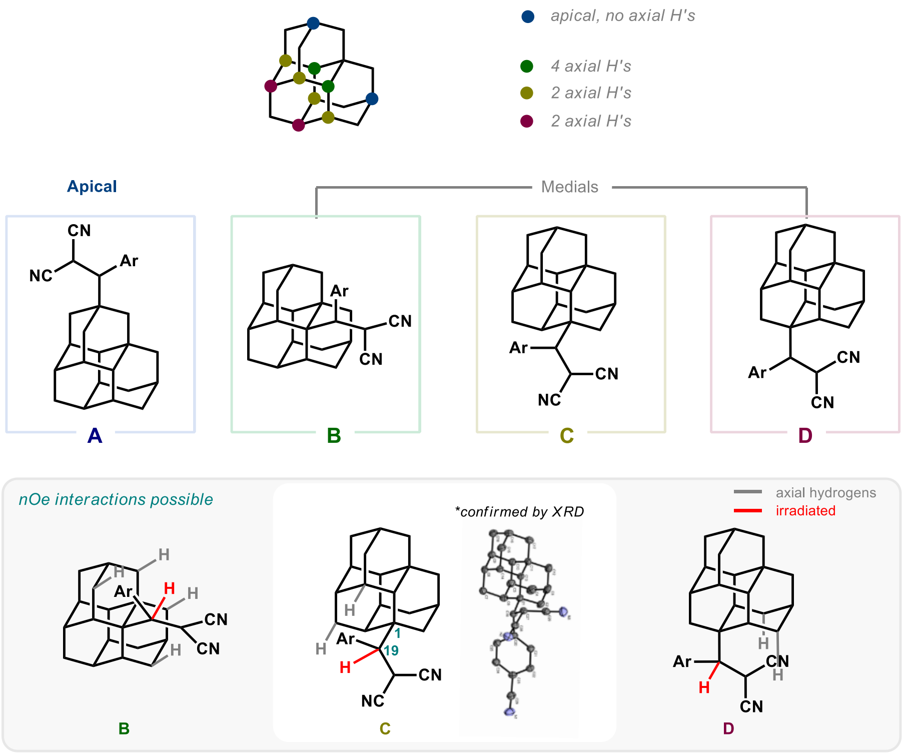

Figure S3. Possible tertiary alkylation products of triamantane and possible nOe interactions.

Shown in **Figure S3** are the four possible alkylation products derived from triamantane.

<u>I. Structural Elucidation using X-Ray Crystallography</u>

XRD quality crystals were obtained via slow diffusion methods. Purified diamondoid products were dissolved in minimal amounts of solvent before being filtered through a plug of cotton into an 8 mL vial. The uncapped vial was then placed inside a larger screwcap glass cylinder filled halfway with sand and excess anti- solvent, allowing for the anti-solvent to slowly defuse diffuse into the vial over time. The lid was screwed on, sealed with parafilm and placed in a stable location free devoid of excessive disruptive vibrations and the vial was checked routinely every 2-3 days until crystals of appropriate size and morphology developed.

After chromatographic isolation of each triamantyl regioisomer, we conducted slow-diffusion recrystallization to obtain single crystals suitable for X-ray crystallography analysis. The resulting crystals were subjected to X-ray diffraction (XRD) to determine precise molecular structure of the product. According to Figure X (above, make proper figures if other structures

acquired), the benzylic methine position (C19) is covalently attached to carbon atom C1, which corresponds to the structure of regioisomer C.

II. 1-Dimensional nOe NMR Analysis

In the context of the reaction involving triamantane, it is important to note that there are three possible medial products and one apical product. Therefore, before characterizing them through selective 1D nuclear Overhauser effect (nOe) and ^{1}H NMR experiments, we wanted to evaluate the possible nOe interactions. For regioisomer B, its benzylic methine proton should be adjacent to four axial protons (**Figure S3**, bottom). Thus, when this proton is irradiated in selective 1D nOe experiments (red), its proximal proton environment should exhibit enhancements for these four axial protons at the least. In the cases of regioisomers C and D, their exocyclic benzylic protons should exhibit proximity to two nearby axial protons. Irradiation at these benzylic positions should lead to prominent enhancement of two pairs of axial hydrogen interactions. Furthermore, because the NMR spectra of the apical product are much simpler and more distinguishable than the other medial isomers, it was not subjected to nOe analysis.

To analyze regioisomer C, selective 1D-nOe experiments were conducted by irradiating the methine hydrogen resonating at δ = 4.01 ppm. The results revealed enhancements of 10% and 19% for a single hydrogen at δ=2.05 ppm and one at δ=2.18 ppm, respectively. Additionally, a small enhancement of 6% was observed for the proton at δ=1.56 ppm. This pattern of nOe enhancements is suggestive of the methine being adjacent to 2 axial hydrogens and at least 2 secondary hydrogens, which is consistent with the structure of regioisomer C. Furthermore, results from XRD analysis further corroborate the identity of this compound as regioisomer C.

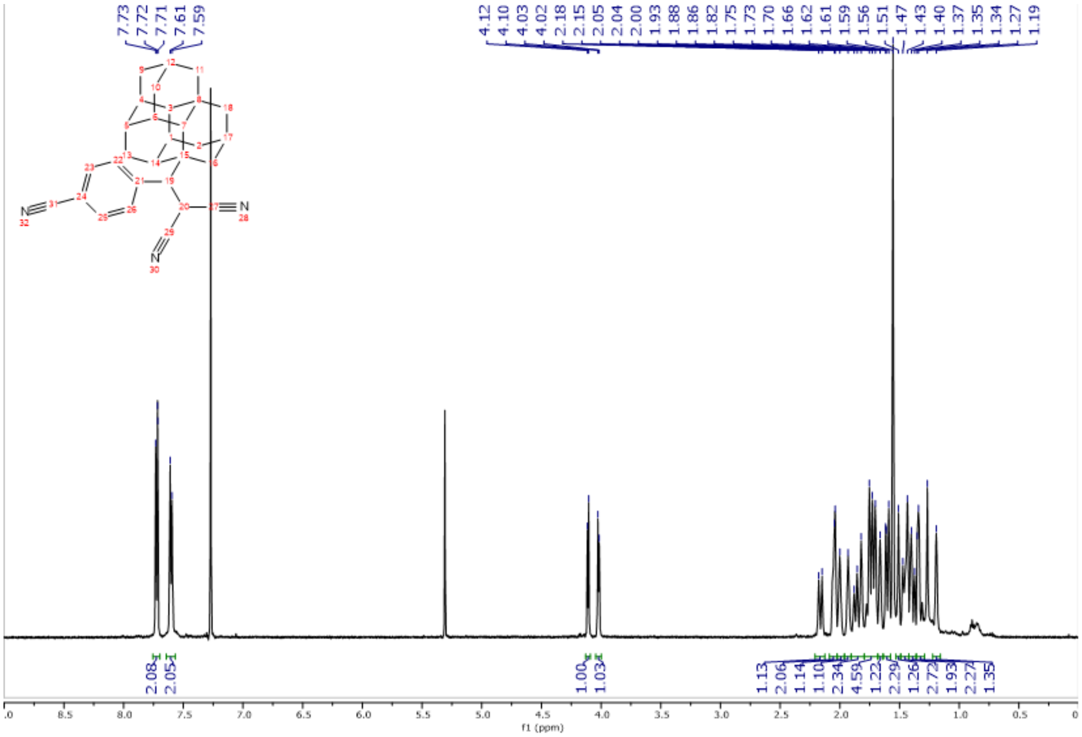

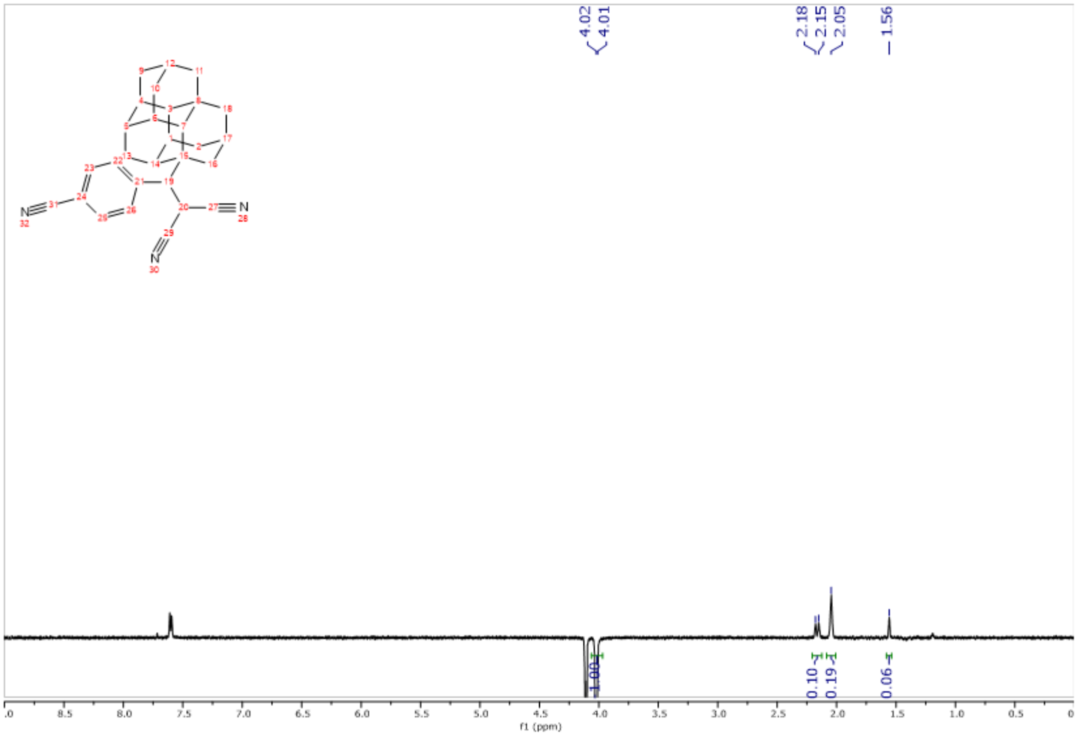

With a mixture labeled regioisomer D, upon irradiation of H1 (at δ=4.05 ppm) was irradiated, the nOe spectrum revealed enhancements of 15% and 9% for nearby hydrogens located at δ2.2 ppm and δ2.0 ppm, respectively. Together with XRD-confirmation of regioisomer B, the

minimal proximity of this benzylic C–H to hydrogens on the cage suggests a C–C attachment with few coaxial C–H bonds. Thus, we assert the identity of this compound to be regioisomer D.

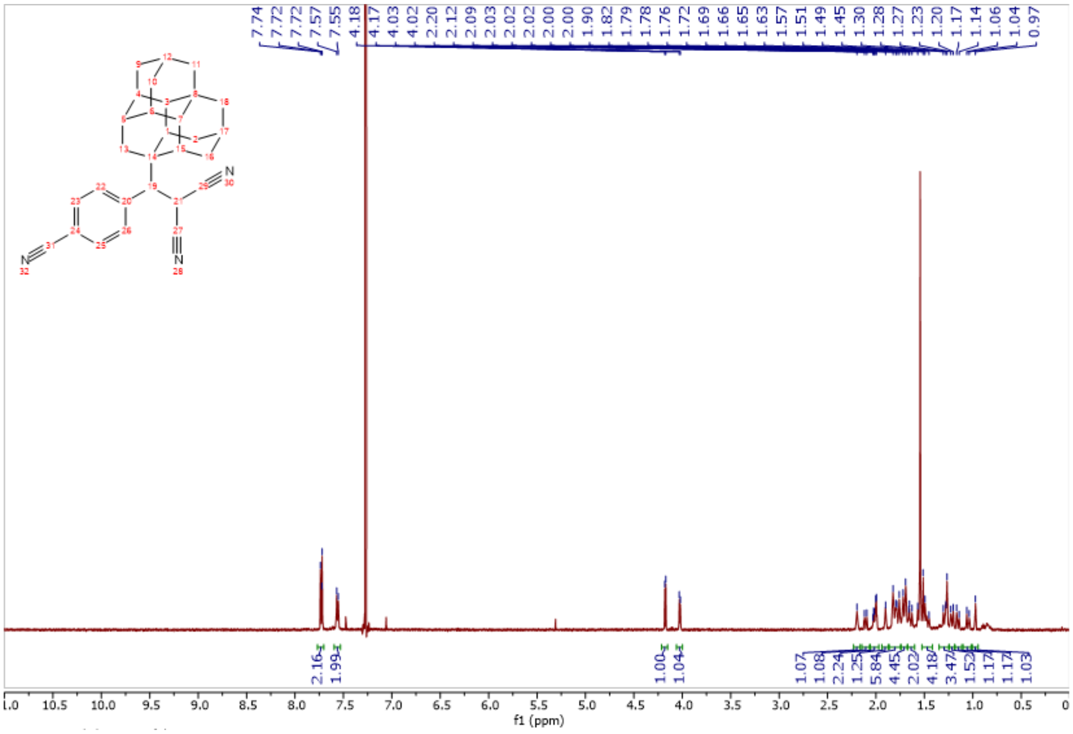

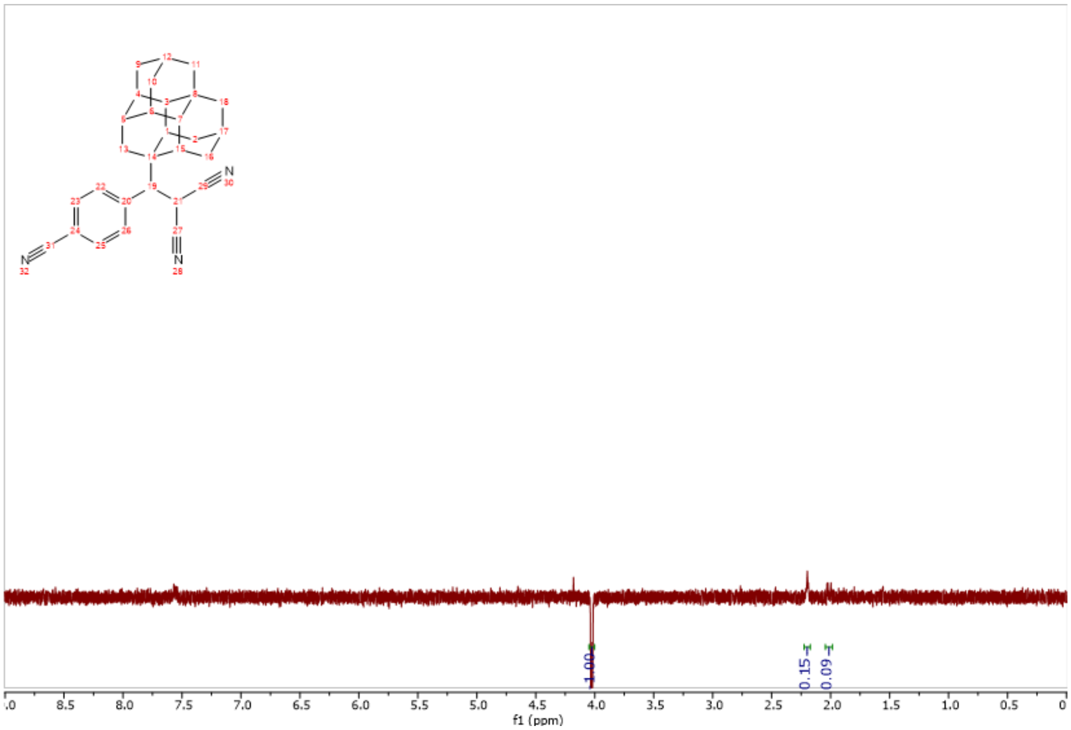

E. Mechanistic Experiments

I. Luminescence Quenching Experiment using Stern-Volmer Analysis

This procedure was adapted from published work by Prof. Mathieu Frenette (UQAM) for the emission quenching measurements of quenchers in solution.[7] A stock solution of catalyst was prepared by dissolving 4.0 mg (0.01 mmol, 2mM) of TPT in 5 mL DCE. To a short-neck quartz cuvette containing 3 mL of DCE, 2.5 µL of the TPT stock solution was added. The cuvette was sealed with a rubber septum. Then, another stock solution of quencher was prepared by dissolving 18.8 mg (0.1 mmol, 10 mM) of diamantane in 10 mL DCE with sonication. Both the cuvette containing the TPT solution and a small vessel containing quencher solution (also sealed with a rubber septum) were degassed by bubbling DCE-saturated nitrogen for 30 min. A gas-tight syringe was pumped and back-filled with the DCE-saturated nitrogen 3 times, loaded with quencher solution (500 µL), and then inserted in the cuvette. The junction was sealed with parafilm to further limit possible introduction of oxygen. Between each increment of injection, the cuvette was gently shaken to mix the quencher solution, and the emission was recorded in the 350-650 nm range using a **Fluoromax-4 Spectro-Fluorometer** with 0.50 mm slits opening and 270 nm excitation. I_0/I was calculated as a ratio between the sums of the total emissions between 350 and 650 nm with and without quencher; it was then plotted against the quencher's concentration with each injection. The quenching rate constant (k_q) was obtained as the slope divided by the lifetime and diamantane concentration, using linear regression according to the Stern-Volmer equation:

$$\frac{I_0}{I} = 1 + k_q \, \tau_0[\text{diamantane}]$$

where I_0 is the luminescence intensity without the quencher, I is the intensity with the quencher, and τ_0 is the lifetime of the photocatalyst. In the case of **TPT, TPP, and T(*p*-FPh)PT**, τ_0 = 4.38 ns.[8] In general, these experiments lend support to the proposed mechanism involving concomitant photooxidation and deprotonation leading to a diamondoid radical. Diamantane was a sufficient quencher for excited state pyryliums including TPT and TPP (**Figure S4**). Their quenching rates with diamantane were calculated to be $2.07 * 10^{10}$ $M^{-1}s^{-1}$ and $1.76 * 10^{10}$ $M^{-1}s^{-1}$, respectively. Given the typical length of a reaction (20-24 hours), these fast rates are somewhat surprising.

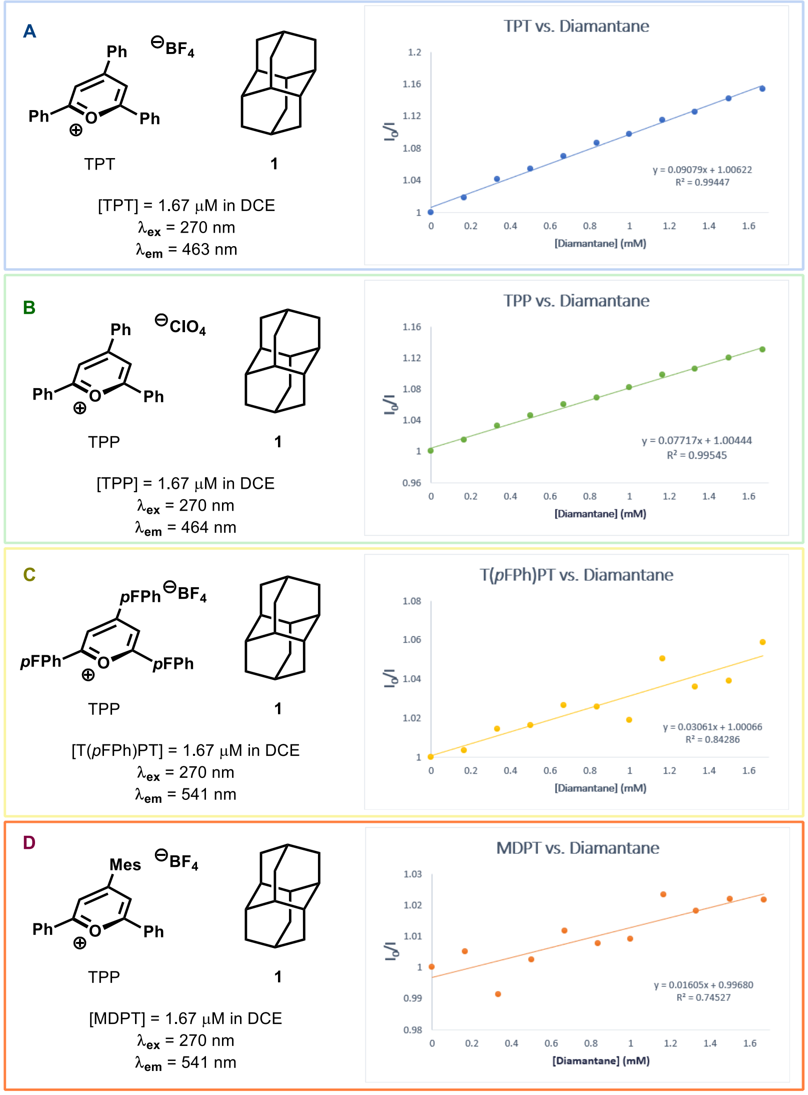

Figure S4

- Stern-Volmer plots of pyrylium photocatalysts with diamantane.

Furthermore, 4-mesityl-2,6-diphenylpyrylium tetrafluoroborate was predicted to be a sufficient catalyst for the oxidation of diamantane (E_{red} (**MDPT**) = +2.62 V). However, diamantane was an unproductive quencher for **T(*p*FPh)PT**, as evident in the lower quenching rate 6.99 x 10^9 $M^{-1}s^{-1}$. The fluorescence lifetime of **MDPT** was not known so its quenching rate was not evaluated.

Calculation of the rate of quenching: For increased accuracy, significant figures of the slope were extended manually via Excel. With a slope of m = 0.09079 for TPT, k_q was calculated as follows:

TPT:

$I_o/I = 1 + k_q * \tau_0 * [\text{diamantane}]$

$\text{Slope} = k_q * \tau_0$

$k_q = \text{slope}/\tau_0$

$k_q = 0.09079\ \text{mM}^{-1}/4.38 * 10^{-9}\ \text{s} * (1000\ \text{mM}\ /1\ \text{M})$

$k_q = 2.07 * 10^{10}\ \text{M}^{-1}\text{s}^{-1}$

Other pyrylium quenching rates were calculated in a similar manner.

TPP:

$k_q = 0.07717\ \text{mM}^{-1}/4.38 * 10^{-9}\ \text{s} * (1000\ \text{mM}\ /1\ \text{M})$

$k_q = 1.76 * 10^{10}\ \text{M}^{-1}\text{s}^{-1}$

T(*p*FPh)PT:

$k_q = 0.03061\ \text{mM}^{-1}/4.38 * 10^{-9}\ \text{s} * (1000\ \text{mM}\ /1\ \text{M})$

$k_q = 6.99 * 10^{9}\ \text{M}^{-1}\text{s}^{-1}$

Luminescence quenching of the benzylidene coupling partner **8** with excited state TPP was also probed by Stern-Volmer analysis (**Figure S5**). For this, a stock solution of catalyst was prepared by dissolving 0.8 mg (0.002 mmol, 2mM) of TPT in 5 mL DCM. To a short-neck quartz cuvette containing 3 mL of DCM, 2.5 μL of the TPT stock solution was added. The cuvette was sealed with a rubber septum. Then, another stock solution of quencher was prepared by dissolving 179.2 mg (1 mmol, 100 mM) of diamantane in 10 mL DCM with sonication. Both the cuvette and vessel containing quencher solution were degassed for 30 min. A gas-tight syringe was pumped and back-filled with the DCM-saturated nitrogen 3 times, loaded with quencher solution (100 μL), and then inserted in the cuvette. The junction was sealed with parafilm to further limit possible introduction of oxygen. The emission was recorded in the 385-650 nm range with 0.50 mm slits opening and 370 nm excitation.

Because the benzylidene absorbs in the UV region, these experiments were done with an excitation wavelength of 370 nm, to mitigate interference by its excited state. Conducting measurements in DCM led to a quenching plot with a flat non-positive line, suggesting that TPP's fluorescence is not effectively quenched by alkylidene **8**. Therefore, the benzylidene malononitrile was determined not to be an efficient quencher and alternative pathways involving electron or energy transfer between excited state pyrylium and benzylidene are hereby ruled out.

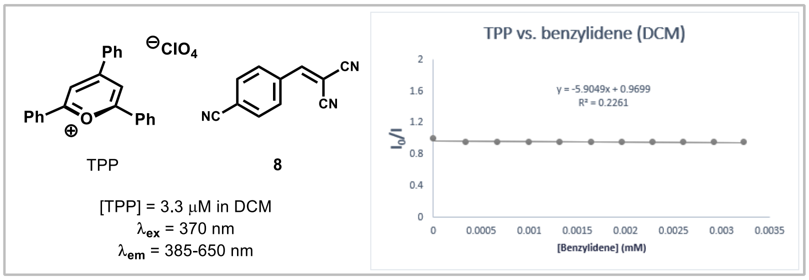

Figure S5.

- Stern-Volmer plots of TPP with benzylidene **8**.

Given the negative slopes for both Stern-Volmer plots in **Figure S5**, the quenching rate of TPP in the presence of benzylidene quencher is zero.

All of the raw luminescence quenching data for the four pyryliums tested with diamantane quencher are tabulated in **Tables S10–S13**. Quenching data for TPP in the presence of benzylidene **8** can be found in **Table S14**.

Table S10. Raw luminescence quenching data for TPT vs. diamantane										
[**1**] (mM)	0	0.164	0.323	0.476	0.625	0.769	0.909	1.05	1.18	1.30
I_0/I	1	1.0063	1.0234	1.0318	1.0418	1.054	1.0547	1.0655	1.0715	1.0715

Table S11. Raw luminescence quenching data for TPP vs. diamantane										
[**1**] (mM)	0	0.167	0.333	0.500	0.667	1.00	1.17	1.33	1.50	1.67
I_0/I	1	1.0146	1.0323	1.0458	1.0602	1.0687	1.0818	1.0910	1.1052	1.1194

Table S12. Raw luminescence quenching data for T(*p*FPh)PT vs. diamantane										
[**1**] (mM)	0	0.167	0.333	0.500	0.667	1.00	1.17	1.33	1.50	1.67
I_0/I	1	1.0033	1.0141	1.0161	1.0264	1.0257	1.0187	1.0502	1.0357	1.0389

Table S13. Raw luminescence quenching data for MDPT vs. diamantane										
[**1**] (mM)	0	0.167	0.333	0.500	0.667	1.00	1.17	1.33	1.50	1.67
I_0/I	1	1.0051	0.9913	1.0025	1.0117	1.0076	1.0010	1.0234	1.0181	1.0217

Table S14. Luminescence quenching data for TPP vs. benzylidene in DCM (average of 3 trials)											
[**8**] (mM)	0	0.332	0.662	0.99	1.316	1.639	1.961	2.28	2.597	2.913	3.226
I_0/I	1	0.9596	0.9551	0.9547	0.9547	0.9549	0.9560	0.9555	0.9561	0.9572	0.9594

II. Electron Paramagnetic Resonance (EPR) Spectroscopy

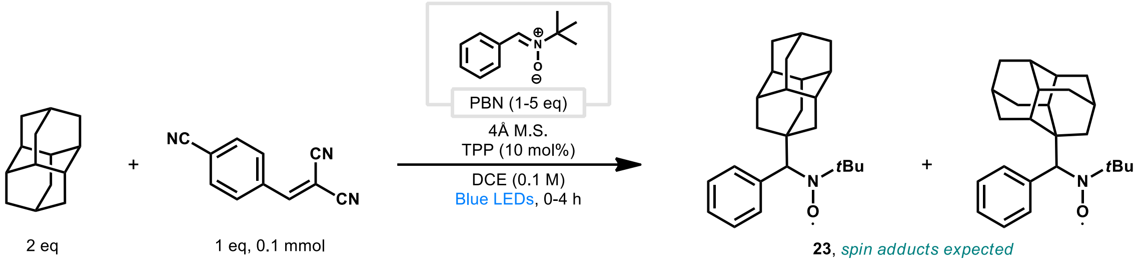

Photoreactions were set up according to **General Procedure B**, using 4-cyanobenzylidene malononitrile as the radical acceptor, along with an equimolar or excess amount of the spin-trap *N*-*tert*-butyl-α-phenylnitrone (CAS#: 3376-24-7) (PBN, 0.1-0.5 mmol). After the vials were sealed and degassed, they were then stirred at room temperature either in the dark or under irradiation between a pair of blue (456 nm) 40 W Kessil lamps.

Electron paramagnetic resonance (EPR) spectroscopy in conjunction with PBN-spin trapping was used to probe the formation of carbon-centered radicals in the photoreaction system. EPR spectra were gathered using a Bruker EMX X-band ESR Spectrometer equipped with an ER 4119HS high-sensitivity cavity. R instrument settings were: Center field, 3508.3 G (using g = 2.0046 for *para*-benzosemiquinone in water as standard); sweep with of 40 G or 60 G. Samples were prepared for EPR analysis by transferring the reaction mixture into a specialized setup of EPR sample tubes. Due to the high dielectric constant of DCE (ε = 10.38)[9], samples were first

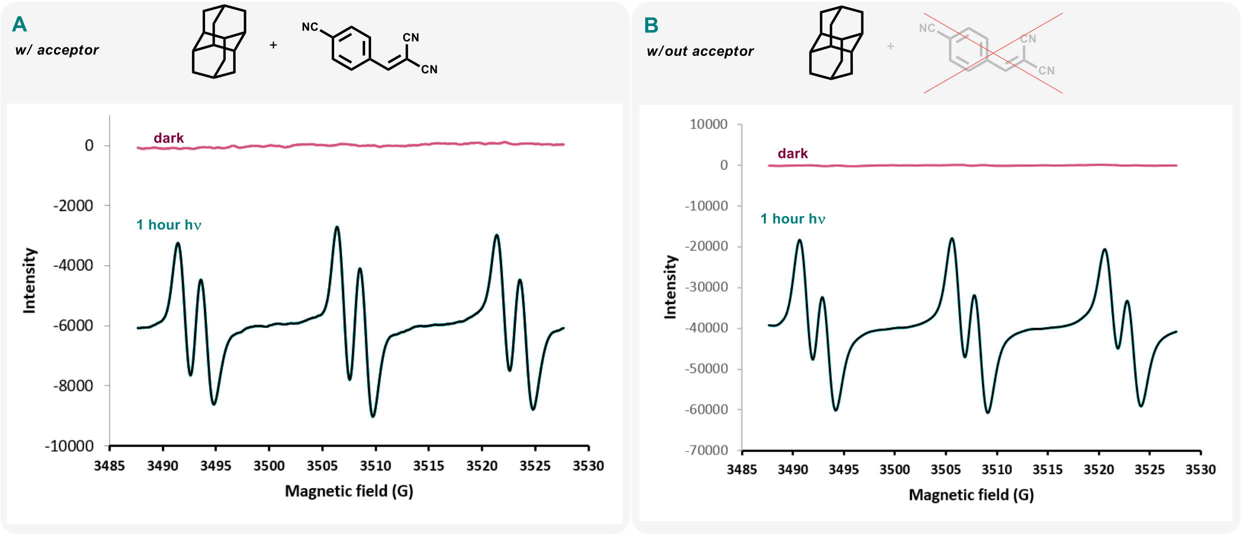

Figure S6.

EPR spectra of diamantane and PBN spin-trap (A) with and (B) without radical acceptor.

loaded into capillary tubes (Hirschmann® melting point tube (100 mm x 0.8 mm x 1 mm) (Sigma-Aldrich, USA), capillary tubes were then supported in quartz EPR sample tube (4 mm O.D., Wilmad-LabGlass, Vineland, NJ) to minimize the interference of the electric field by the solvent. EPR spectra were obtained from reactions set up under different irradiation conditions (dark, 1 hour light, 4 hour light) at room temperature.

In the absence of light only a very weak background signal from a spin adduct of the PBN spin trap ($a^N \approx 14$ G, $a^H \approx 2$ G) was observed, **Figure S6A**. When the photoreaction was irradiated for 1 hour, we observed a robust EPR spectrum of a PBN spin adduct ($a^N \approx 14$ G, $a^H \approx 2$ G). These hyperfine splitting constants are consistent with the spin trapping of carbon-centered radicals by PBN.[10] The symmetry in the spectrum suggests the presence of only one dominant spin adduct. The concentration of the background spin adduct in the dark reaction is less than 2-3% of the spectrum of the irradiated sample.

Given the presence of a signal consistent with trapping of a carbon-centered radical, we wondered if this paramagnetic species could form in the absence of the radical acceptor. Thus, another set of photoreactions were set up again according to **General Procedure B** (with 0.1-0.5 mmol PBN), this time omitting the benzylidene malononitrile. After the vials were sealed and degassed, they were stirred at room temperature either in the dark or under irradiation.

Closely resembling the outcomes of the previous experiments, we observed the presence of noisy signals across the EPR spectrum when the dark reaction was subjected to EPR analysis (**Figure S6B**). Under light irradiation, we observed a distinct splitting into hyperfine lines. Notably, having this event reoccur in the absence of benzylidene malononitrile suggests that the same paramagnetic species is generated with or without the radical acceptor. This is consistent with our proposal that the photocatalyst undergoes a direct oxidation even with the diamondoid hydrocarbon in DCE under visible light. It is conceivable that the diamantane radical cation undergoes deprotonation by the solvent, giving rise to a diamantyl radical. We hypothesize that this diamantyl radical is the identity of the carbon-centered radical detected through EPR in our study.

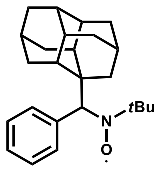

23

HRMS (ESI) m/z calcd for $C_{25}H_{34}NO^+$ $(M)^+$ = 364.2640, found 364.2632.

III. Alkylation of allylic and benzylic C–H donors

Presented in **Table S15** is a survey of allylic and benzylic C–H donors amenable to our reaction conditions with TPT. Tetramethylethylene was successfully alkylated, achieving product in 72% and 67% yield (a & b). Benzylic C–H donors, including *para*-xylene, indane, and *para*-methoxytoluene were well tolerated. *Para*-xylene led to 60-70% alkylated product (c & d), indane led to 64-66%, and *para*-methoxytoluene was least efficient at a yield of 16-30% (e & f). Since these C–H donors have been shown to undergo direct photooxidation followed by proton loss, leading to respective allylic or benzylic radicals.[11] This is further support for our proposed mechanism of photooxidative proton loss.

Table S15. Alkylation of allylic and benzylic C–H donors using benzylidene malononitriles

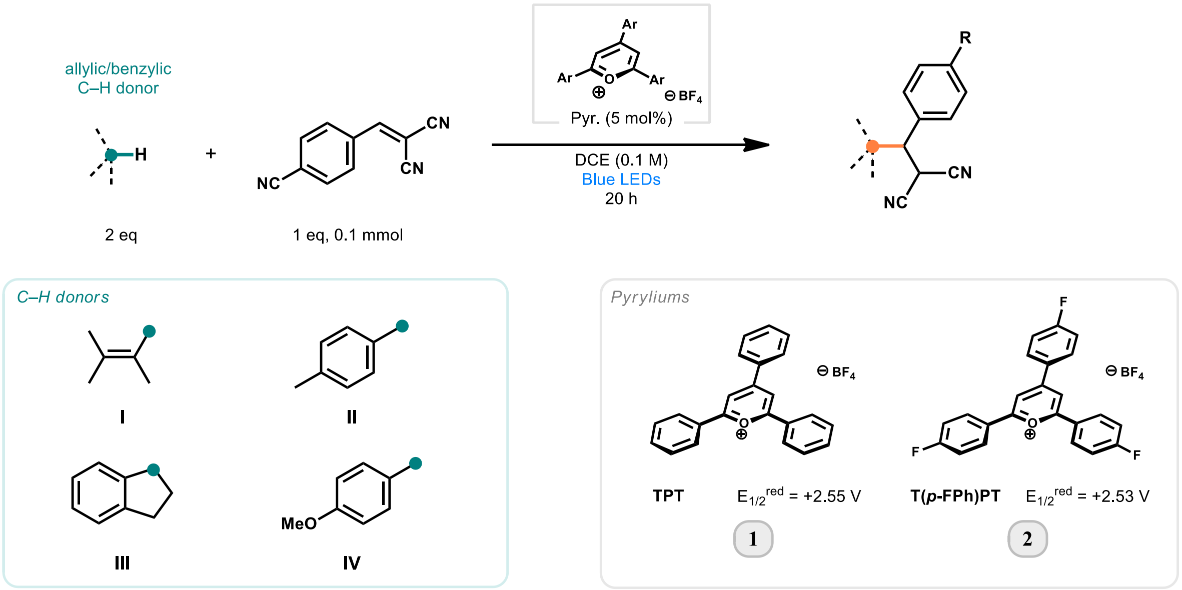

Reaction	Pyrylium	C–H donor	% Yield
a	1	I	72
b	2	I	67
c	1	II	60
d	2	II	70
e	1	III	64
f	2	III	66
g	1	IV	16
h	2	IV	30

Product yield determined by ^{1}H NMR using dibenzyl ether as an internal standard.

IV. Cyclic Voltammetry

Cyclic Voltammetry was performed on Pine research classic WaveNow potentiostat. The cell consisted of a glassy carbon working electrode with a surface area of 0.07 cm^2 . The electrode was polished with an alumina-water slurry as reported by Dempsy and coworkers.[12] A Pt wire auxiliary electrode, and a Pt-wire pseudo-reference electrode with a Fc/ Fc^+ couple as an internal standard correction was used. The data was subsequently adjusted to SCE by the addition of +0.38 V. All potentials are measured in acetonitrile solution of 100 mM tetrabutylammonium hexafluorophosphate.

Experimental cyclic voltammograms for adamantane, diamantane, and triamantane are shown in **Figures S7**, **S8**, and **S9**, respectively. Diamantane (+2.46 V) and triamantane (+2.18 V) are compatible substrates for oxidation by excited state pyrylium.

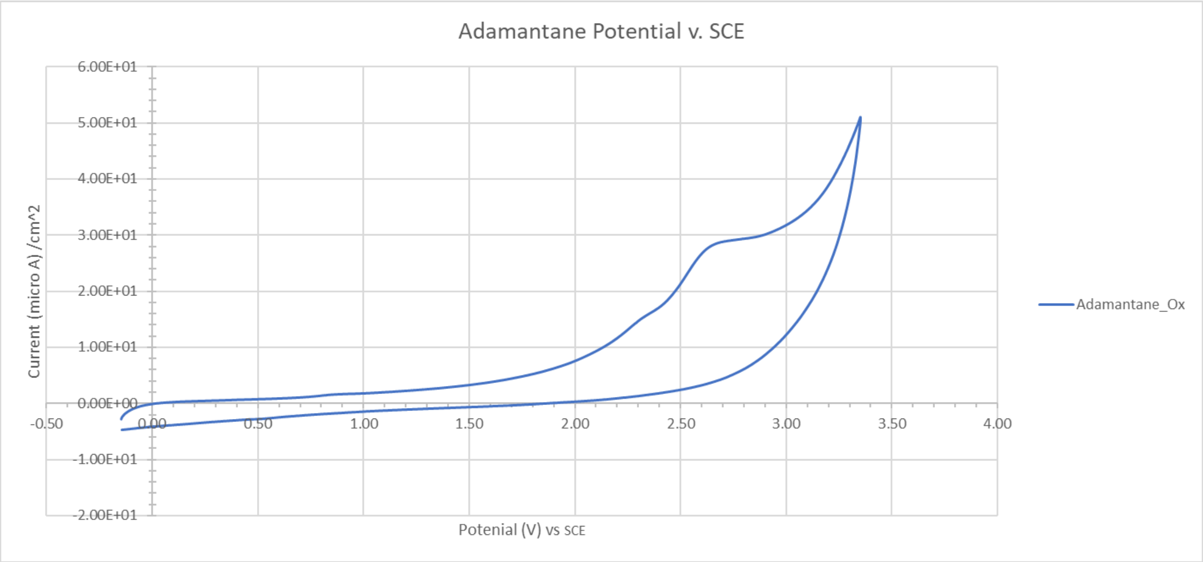

Figure S7: CV of 1 mM adamantane ($E_{1/2}^{red}$ = +2.55 V vs. SCE in CH_3CN)

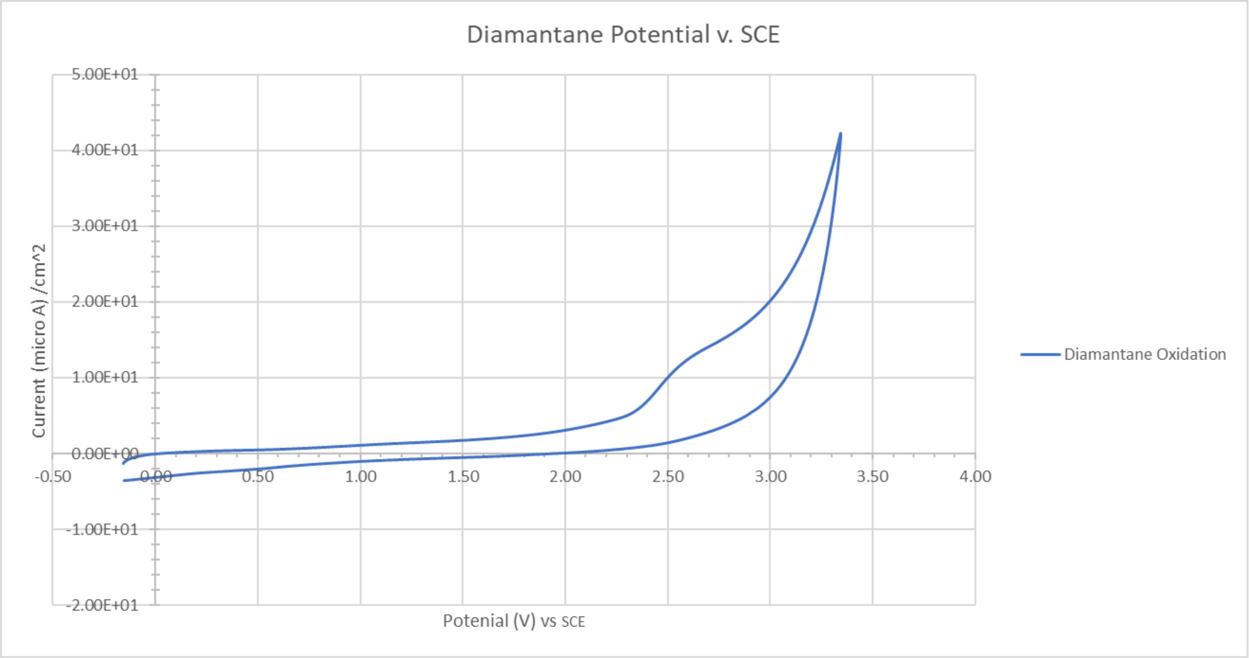

Figure S8: CV of 1 mM diamantane ($E_{1/2}^{red}$ = +2.46 V vs. SCE in CH_3CN)

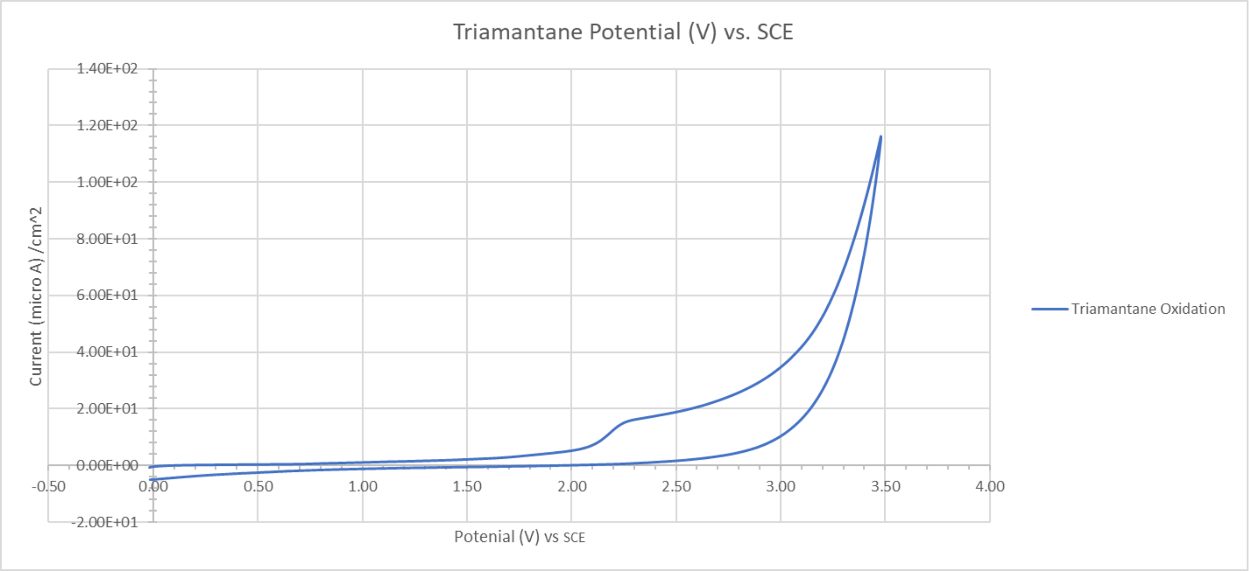

Figure S9: CV of 1 mM triamantane ($E_{1/2}^{red}$ = +2.18 V vs. SCE in CH_3CN)

F. NMR Spectra

2-((Diamant-1-yl)(4-cyanophenyl)methyl)malononitrile (**9**)

Mixture

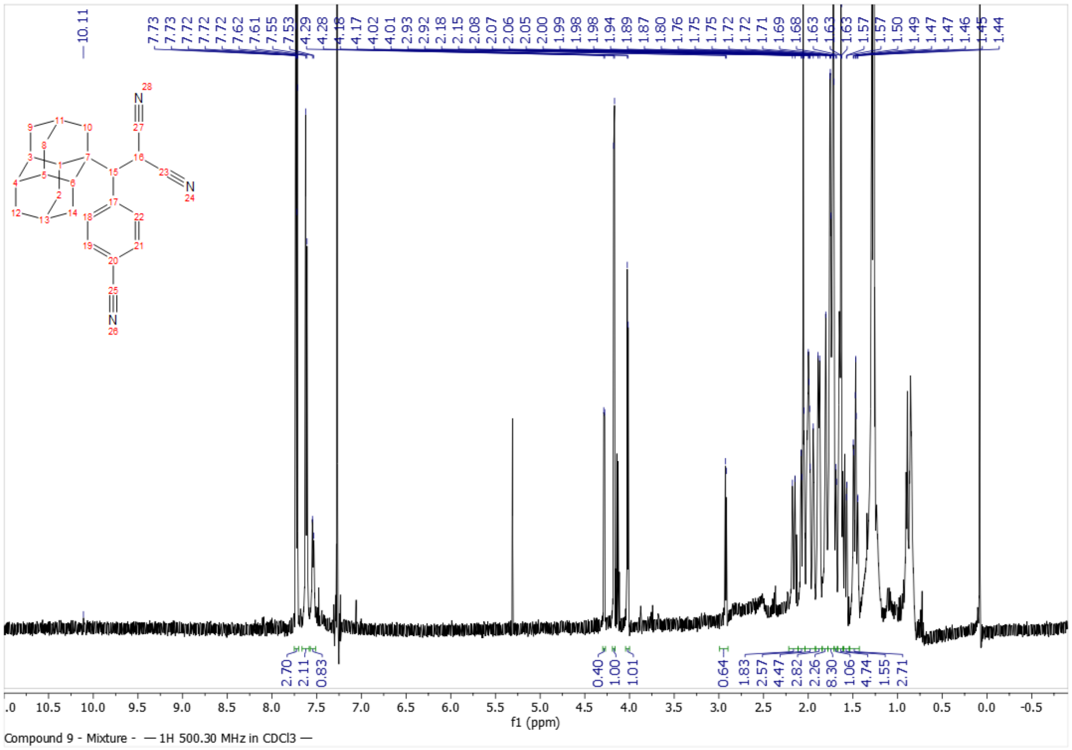

Medial product (**9m**):

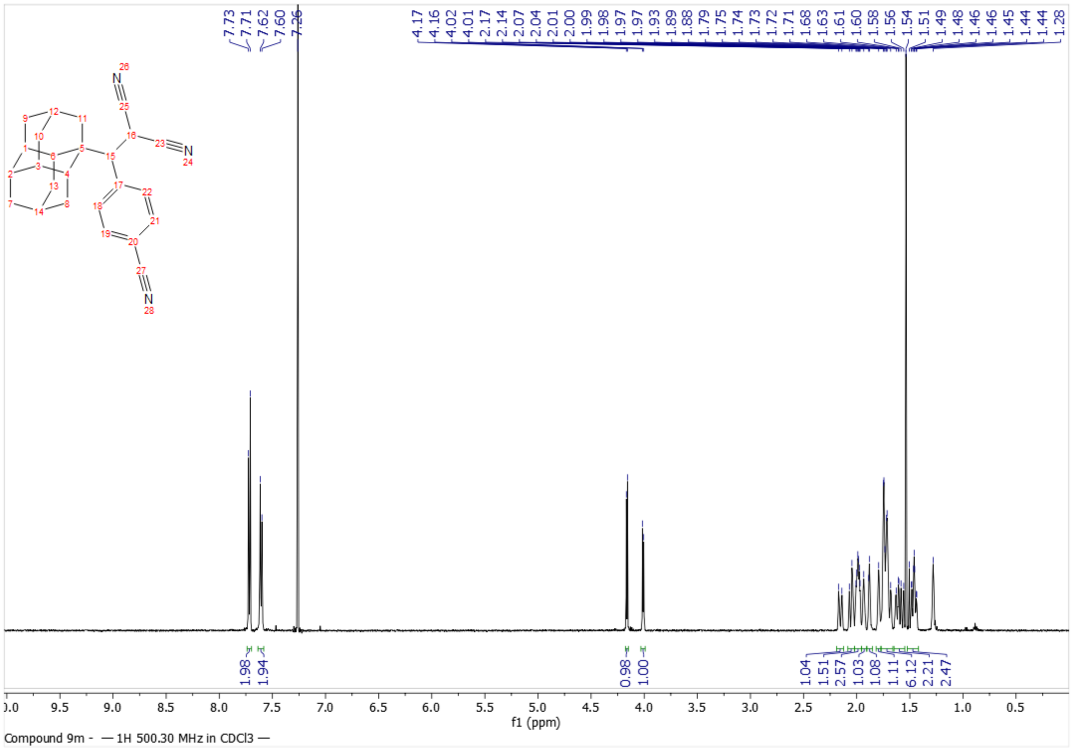

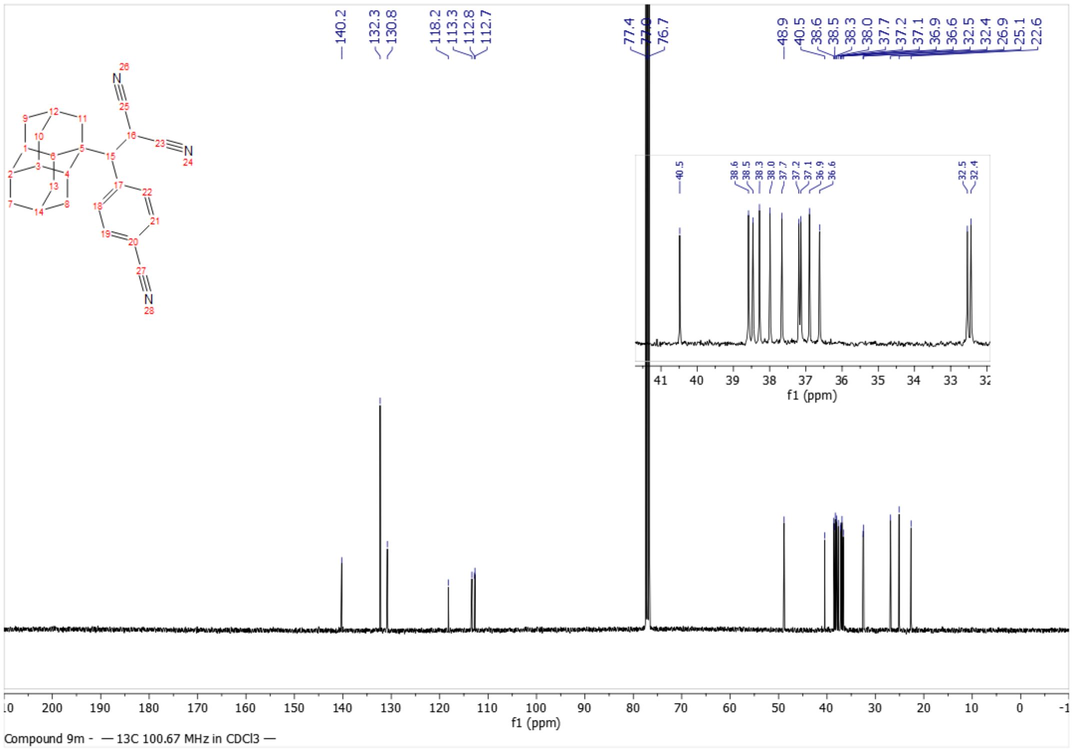

Apical product (**9a**):

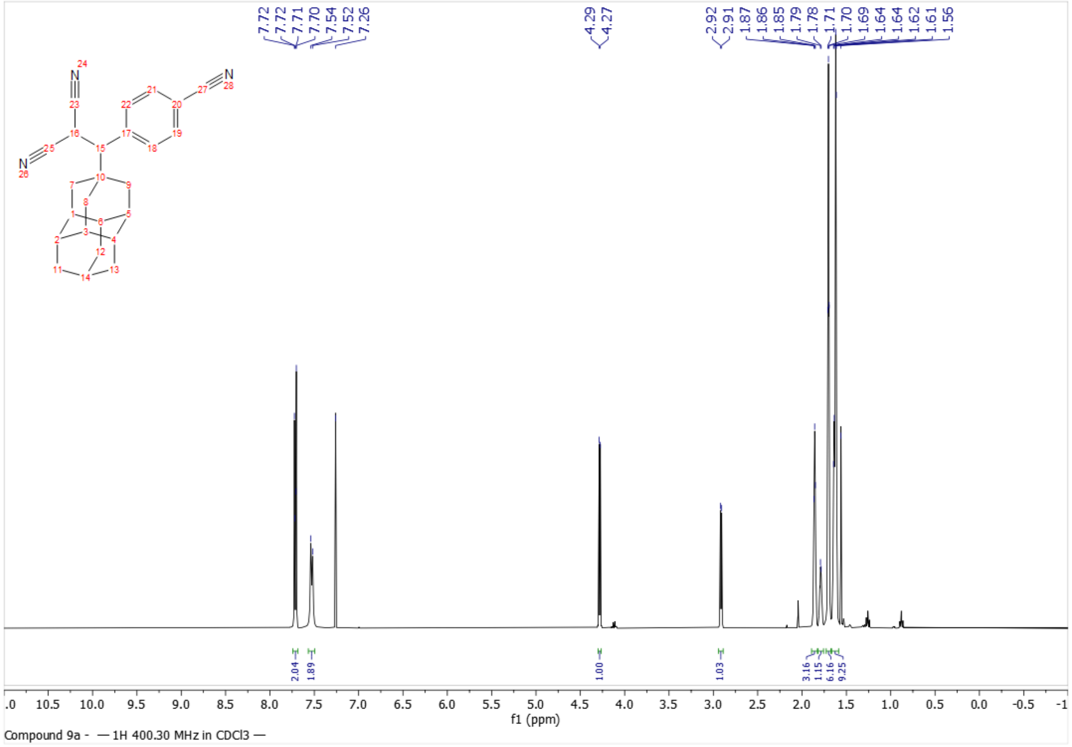

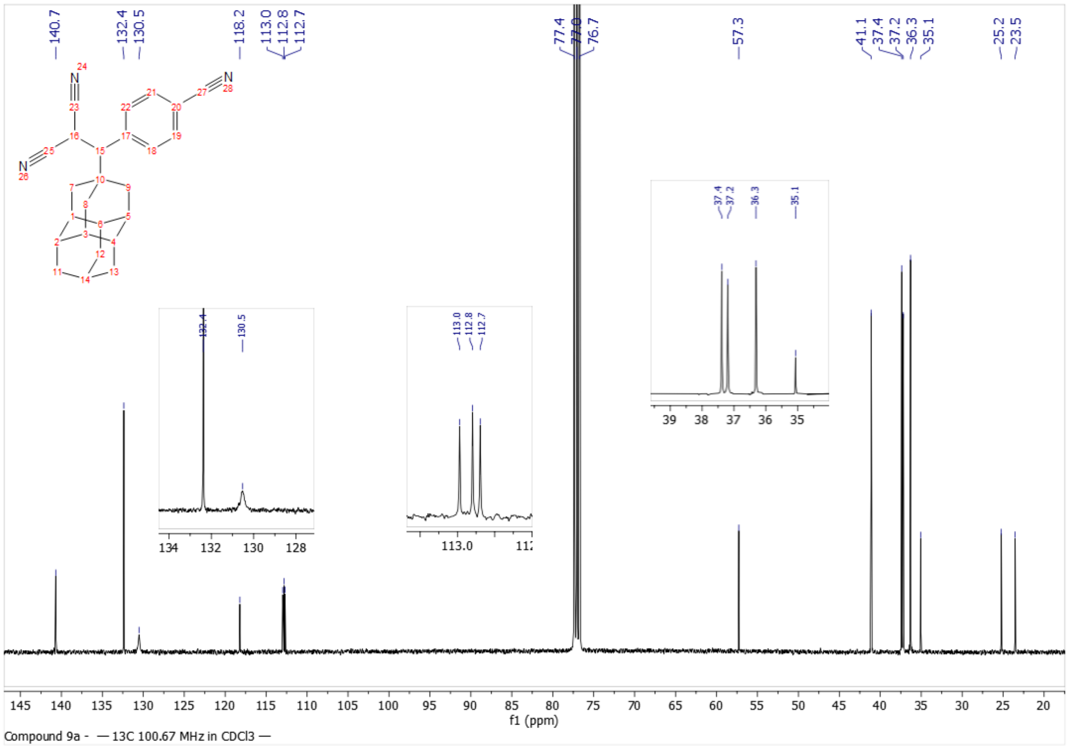

2-((Diamant-1-yl)(4-trifluoromethylphenyl)methyl)malononitrile (**10**)

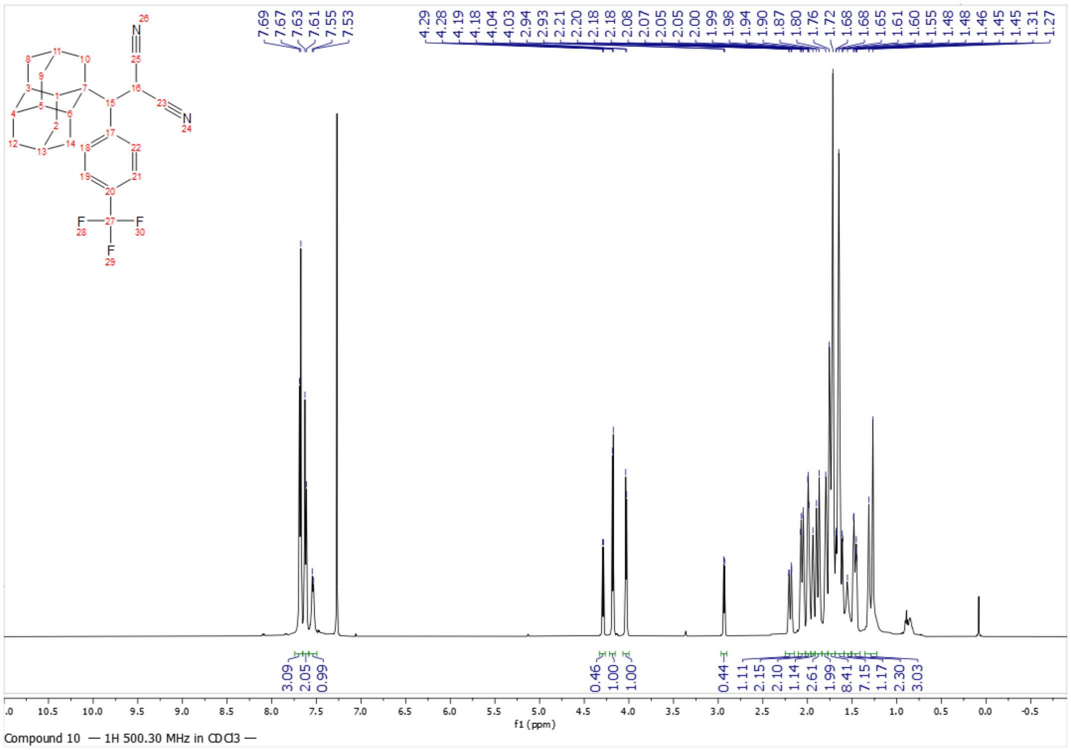

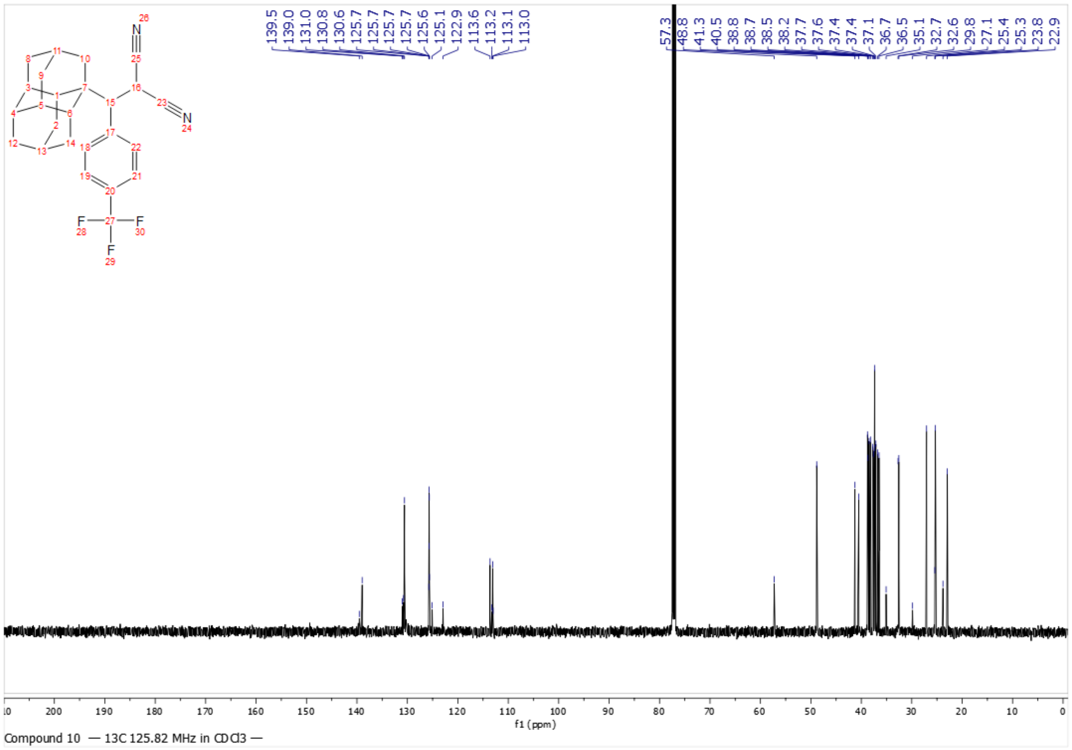

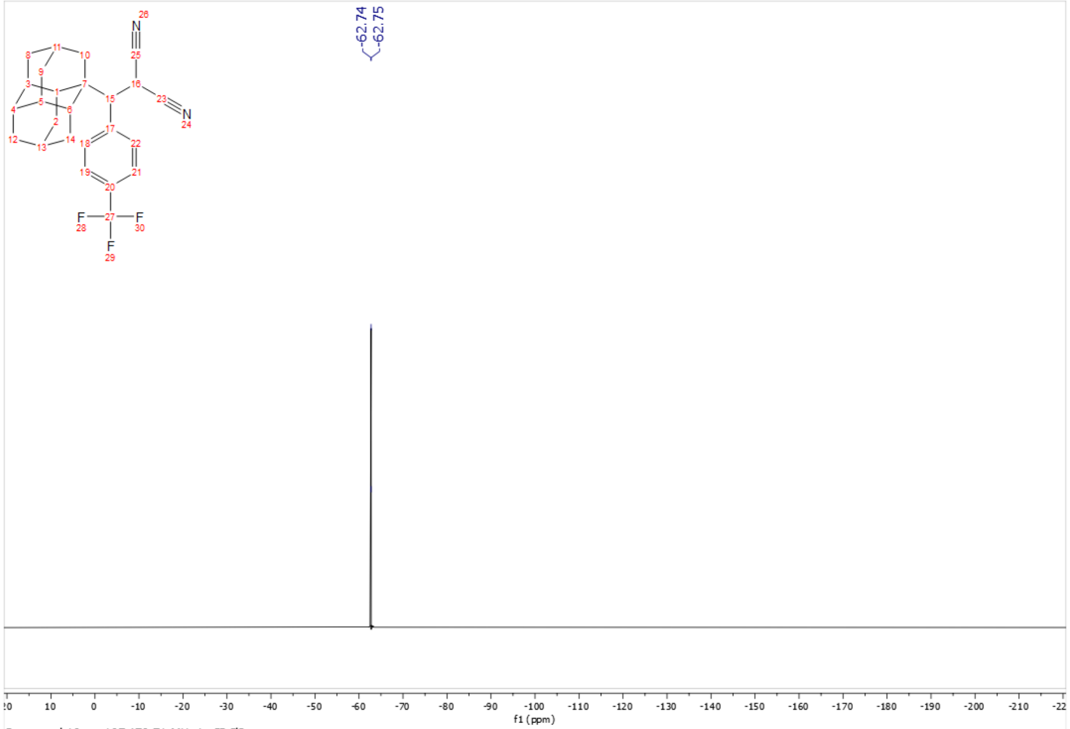

Compound 10 — 19F 470.71 MHz in CDCl3 —

2-((Diamant-1-yl)(4-nitrophenyl)methyl)malononitrile (**11**)

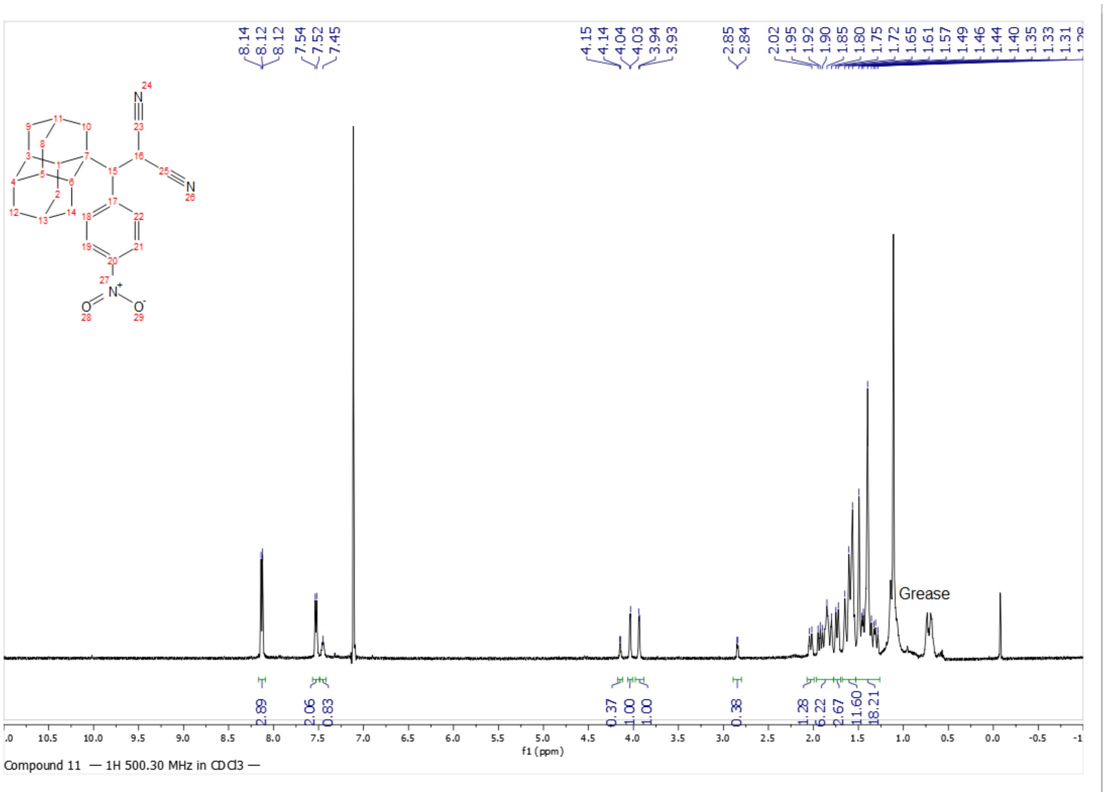

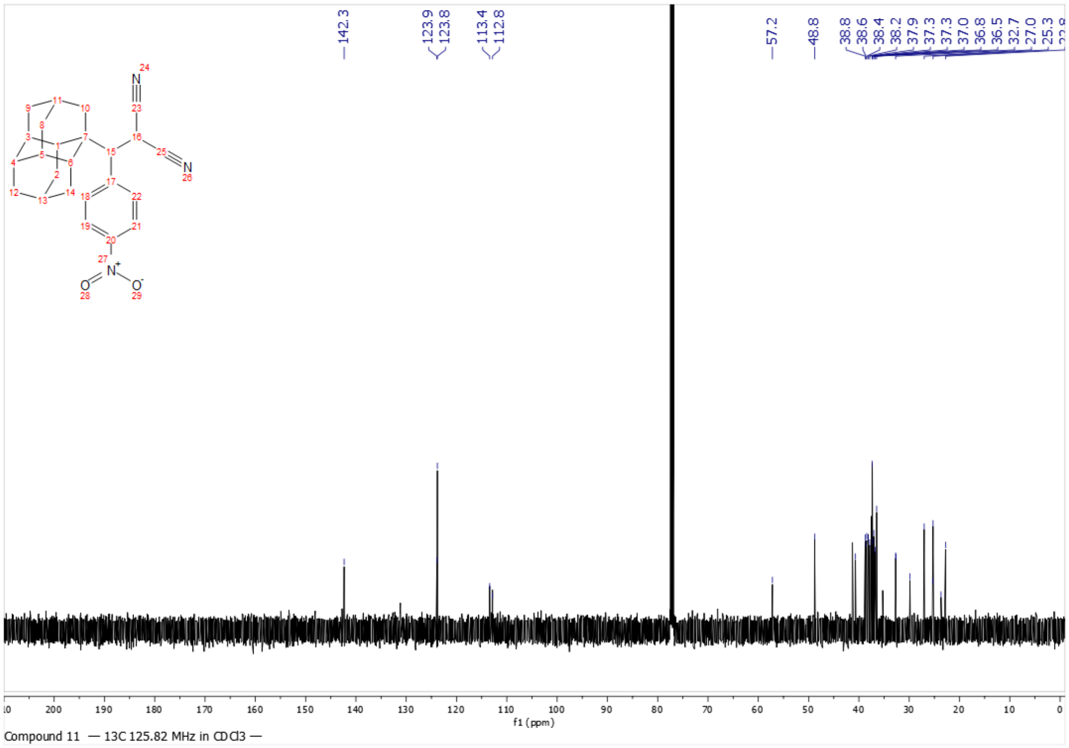

2-((Diamant-1-yl)(4-fluorophenyl)methyl)malononitrile (**12**)

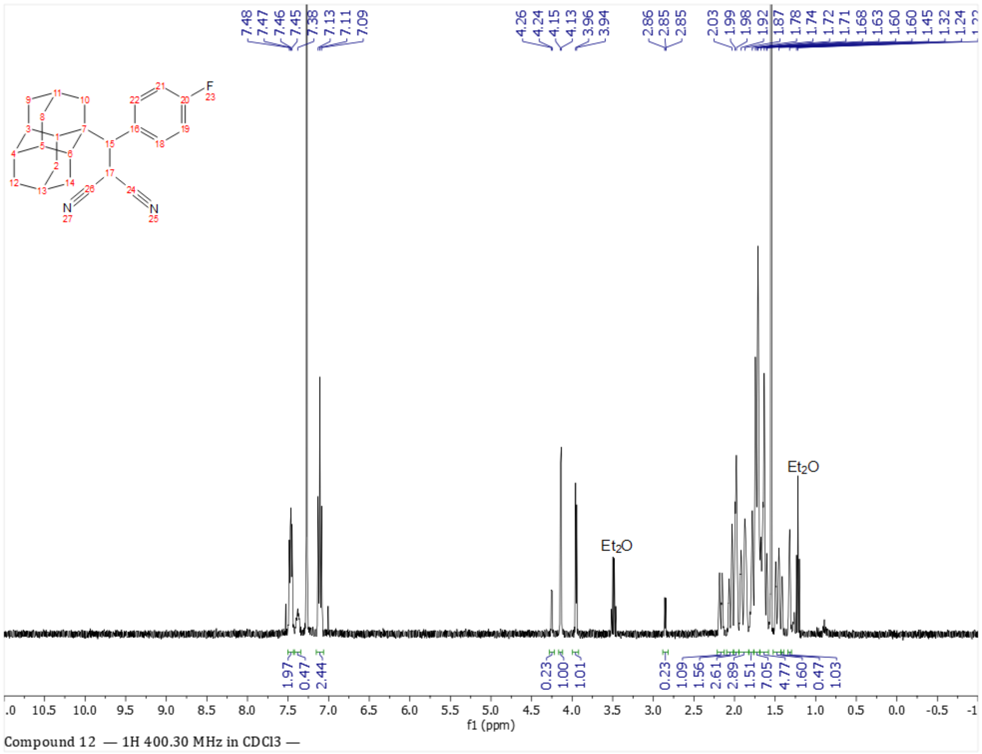

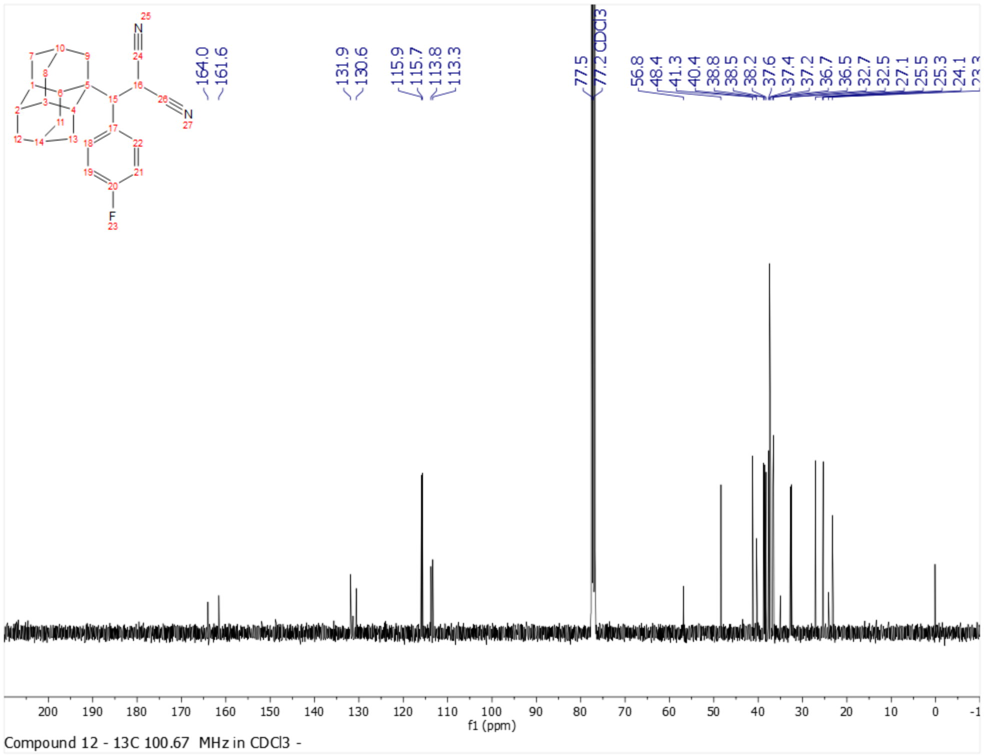

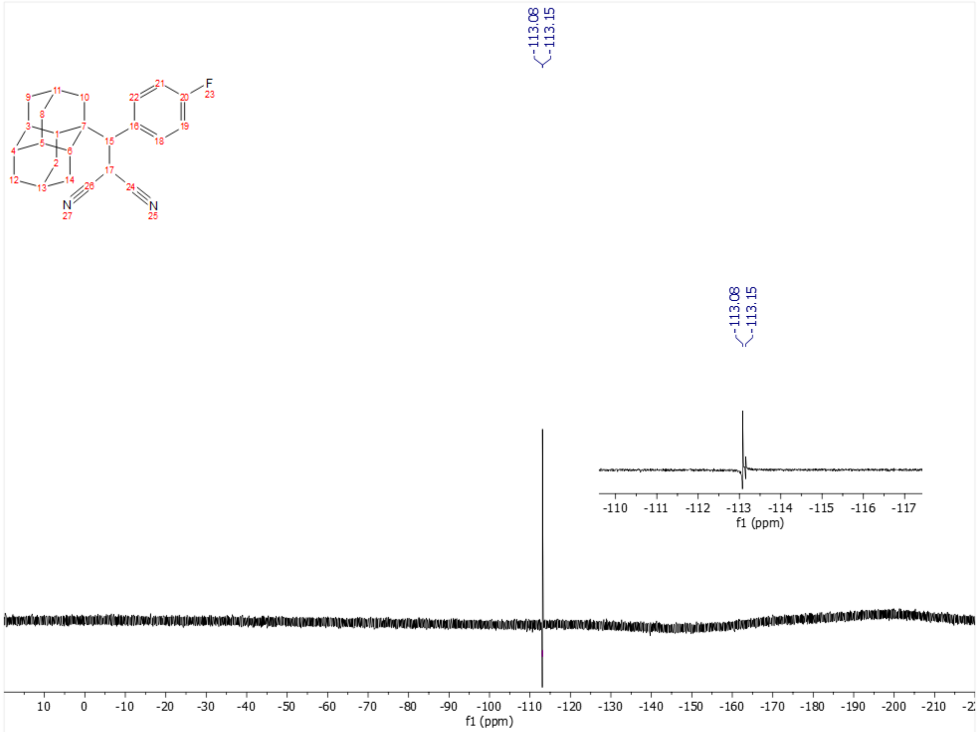

Compound 12 — 19F 282.38 MHz in CDCl3 —

2-((Diamant-1-yl)(4-chlorophenyl)methyl)malononitrile (**13**)

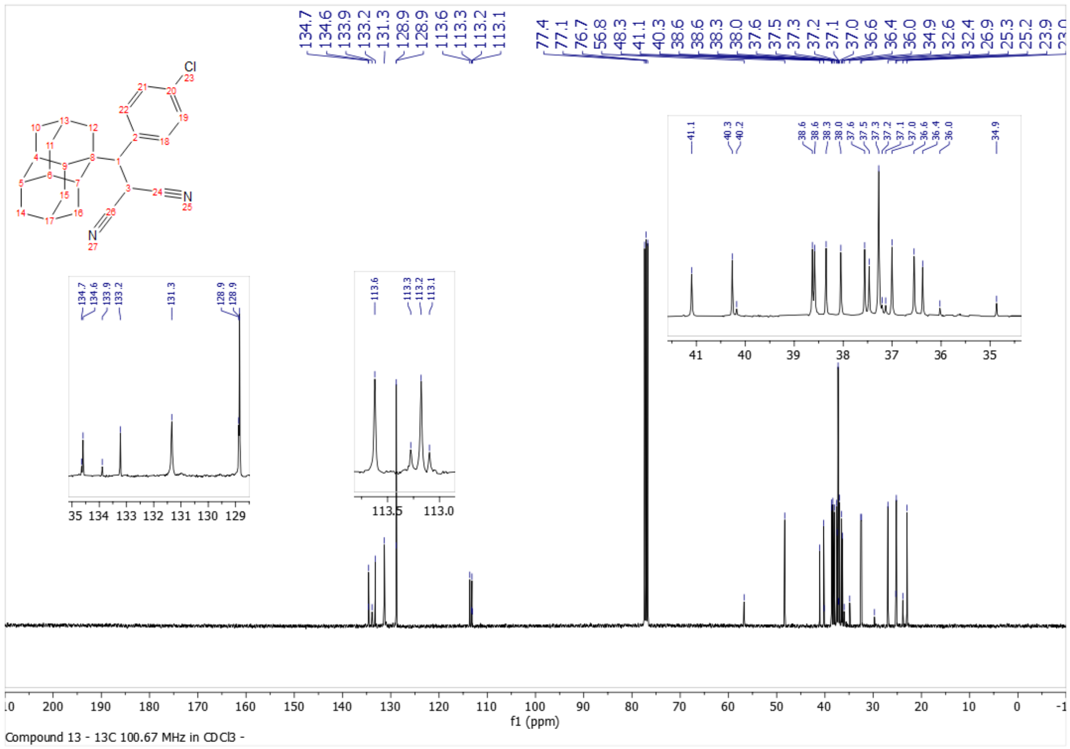

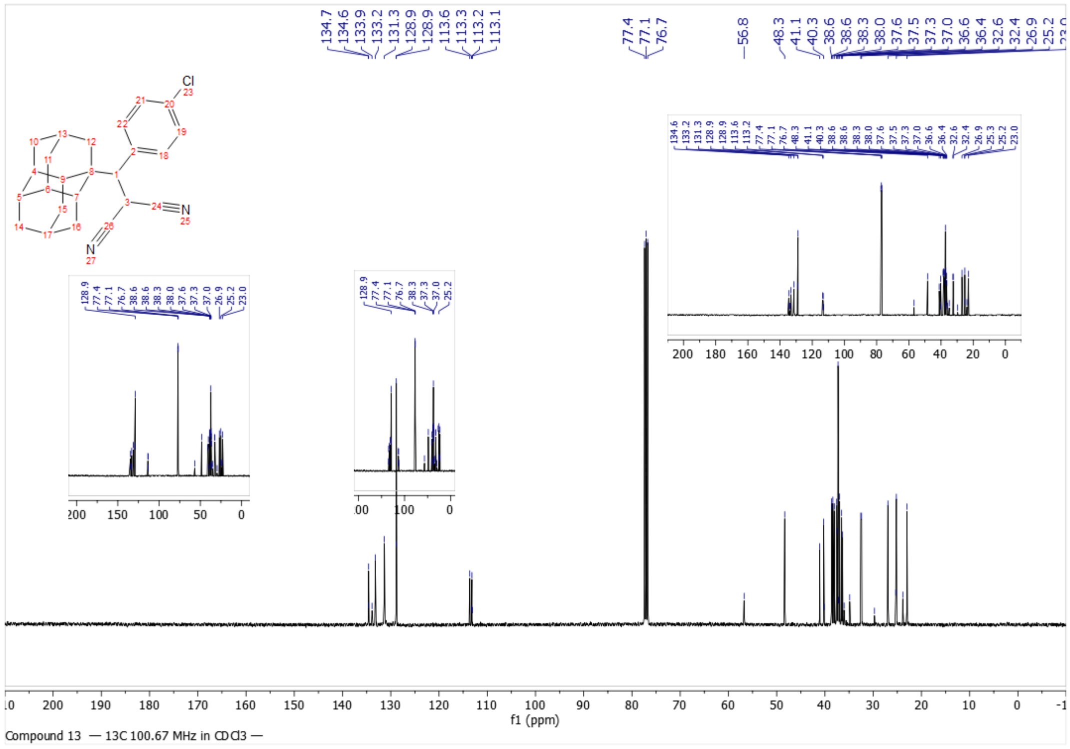

2-((Diamant-1-yl)(4-bromophenyl)methyl)malononitrile (1**4**)

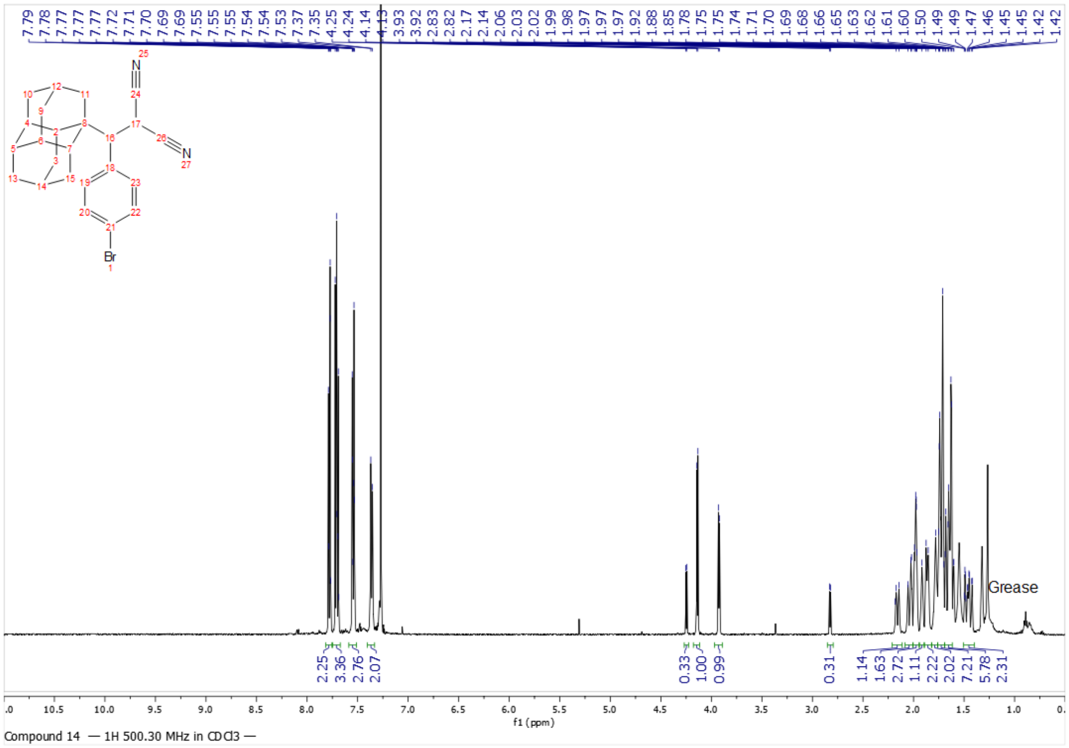

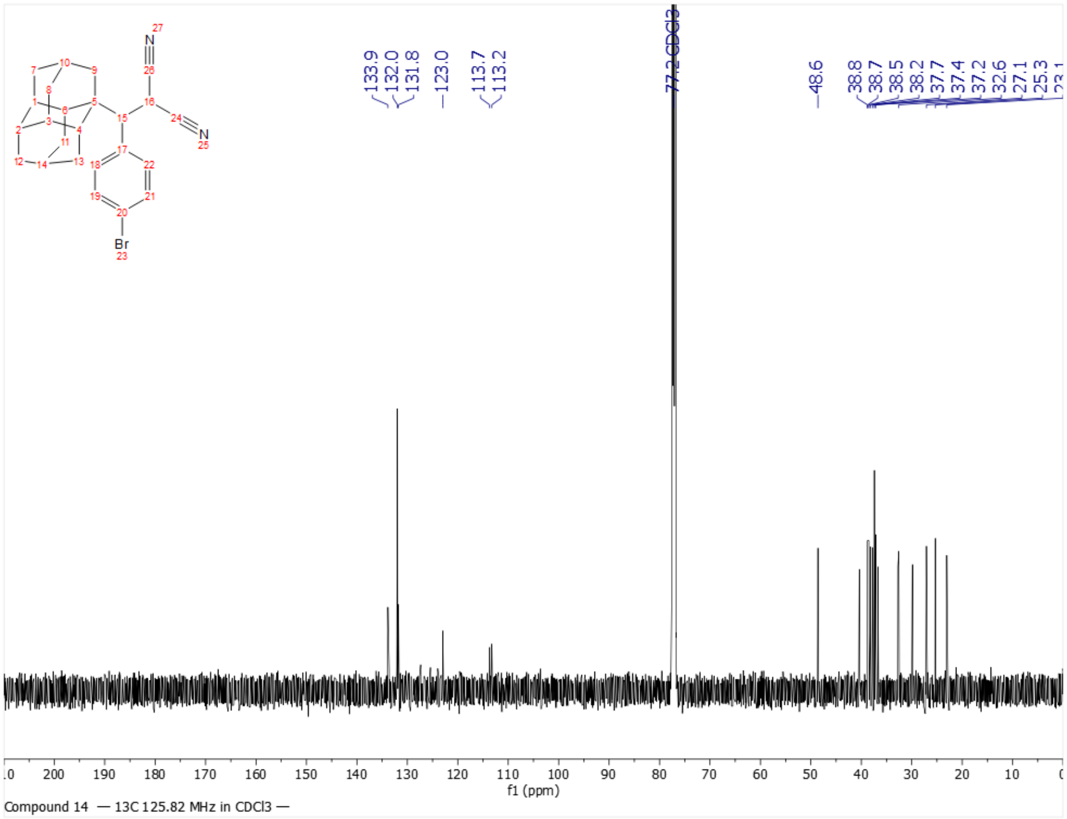

2-((Diamant-1-yl)(phenyl)methyl)malononitrile (**15**)

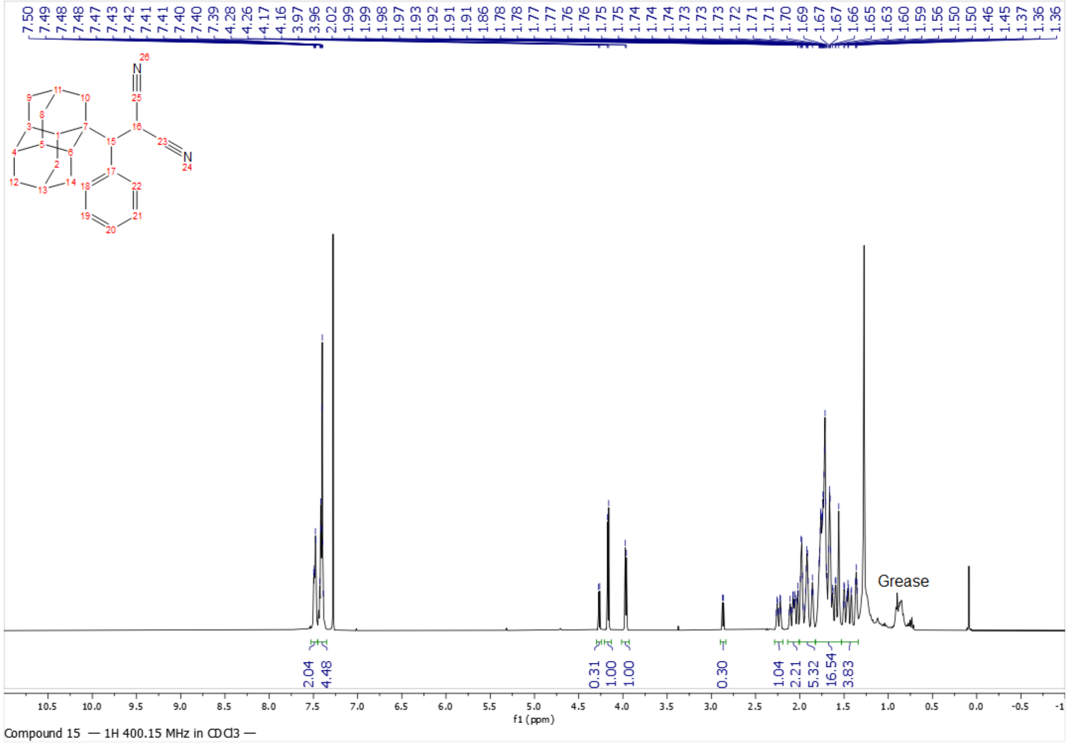

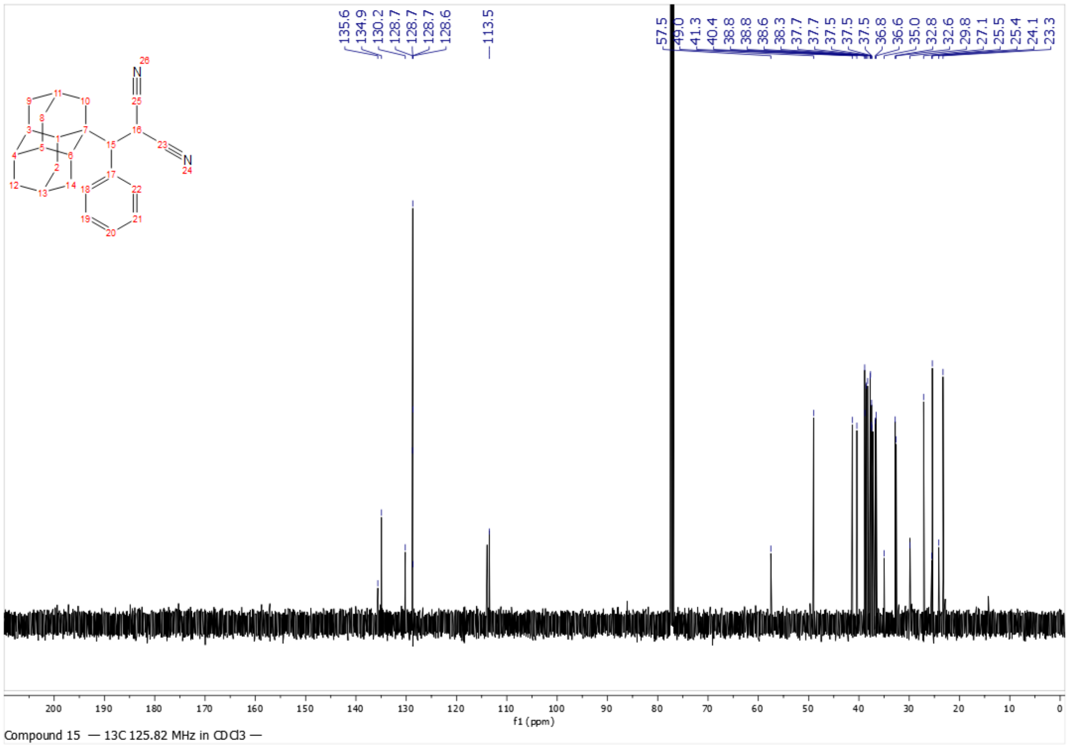

Diethyl 2-(diamant-1-yl)malonate (**16**)

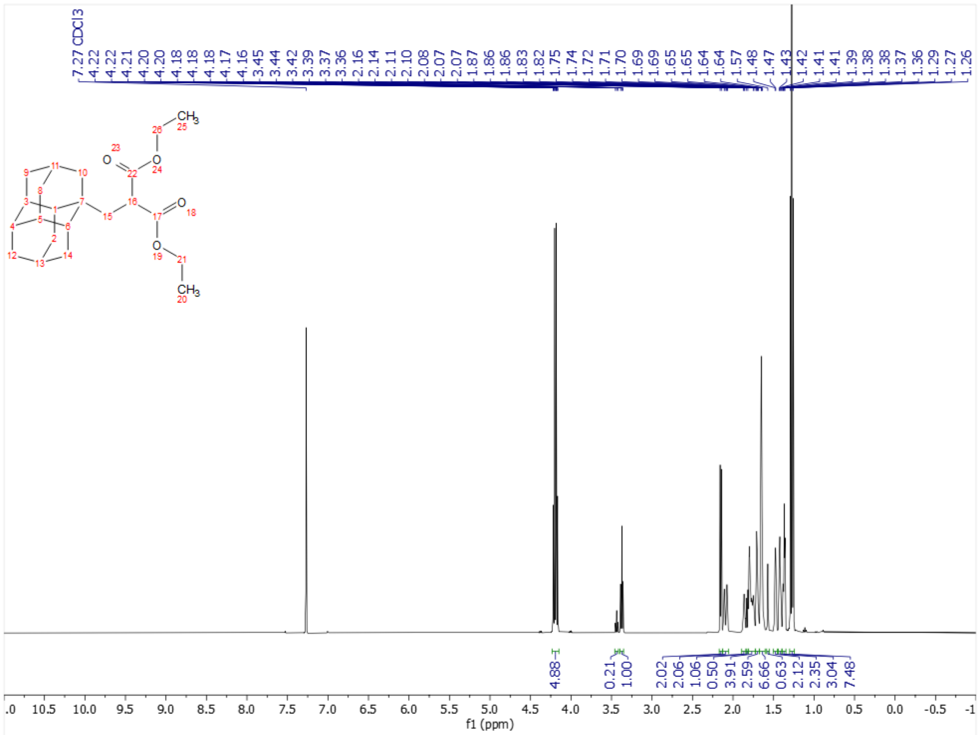

Compound 16 — 1H 400.13 MHz in CDCl3 —

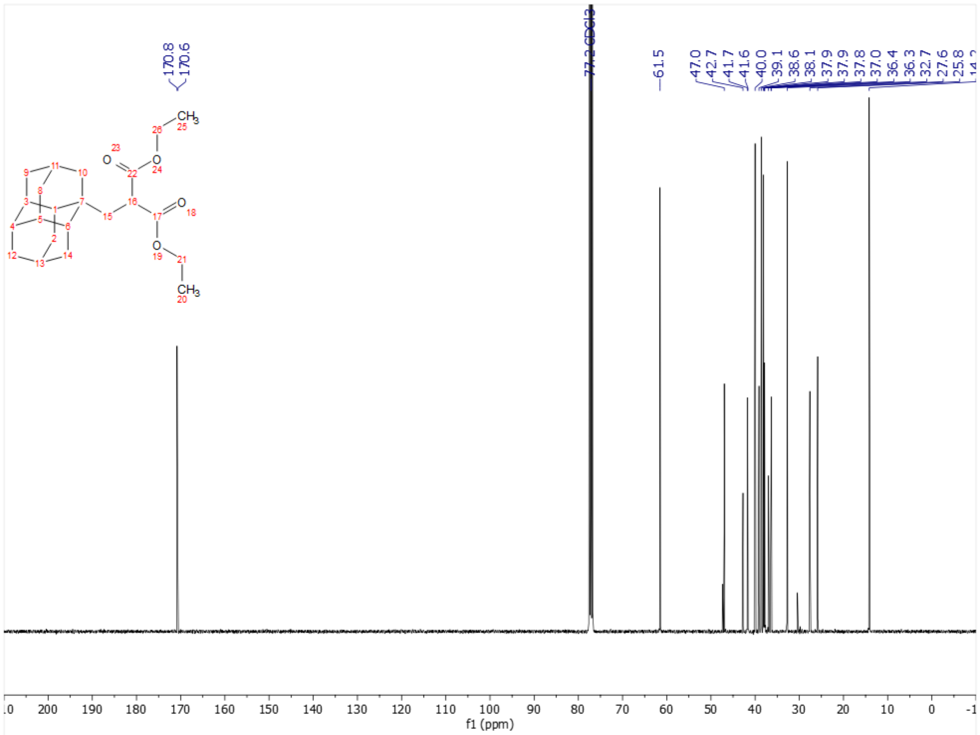

Compound 16 — 13C 100.67 MHz in CDCl3 —

2-((Diamant-1-yl)-1,1-bis(phenylsulfonyl)ethane (**17**)

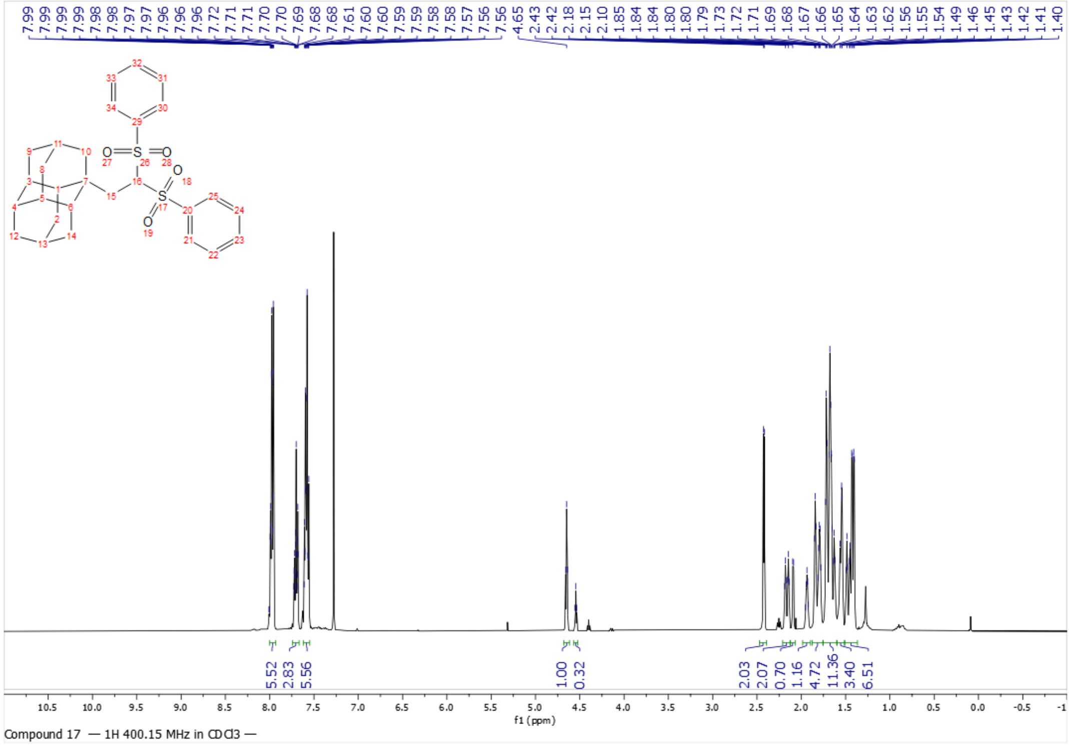

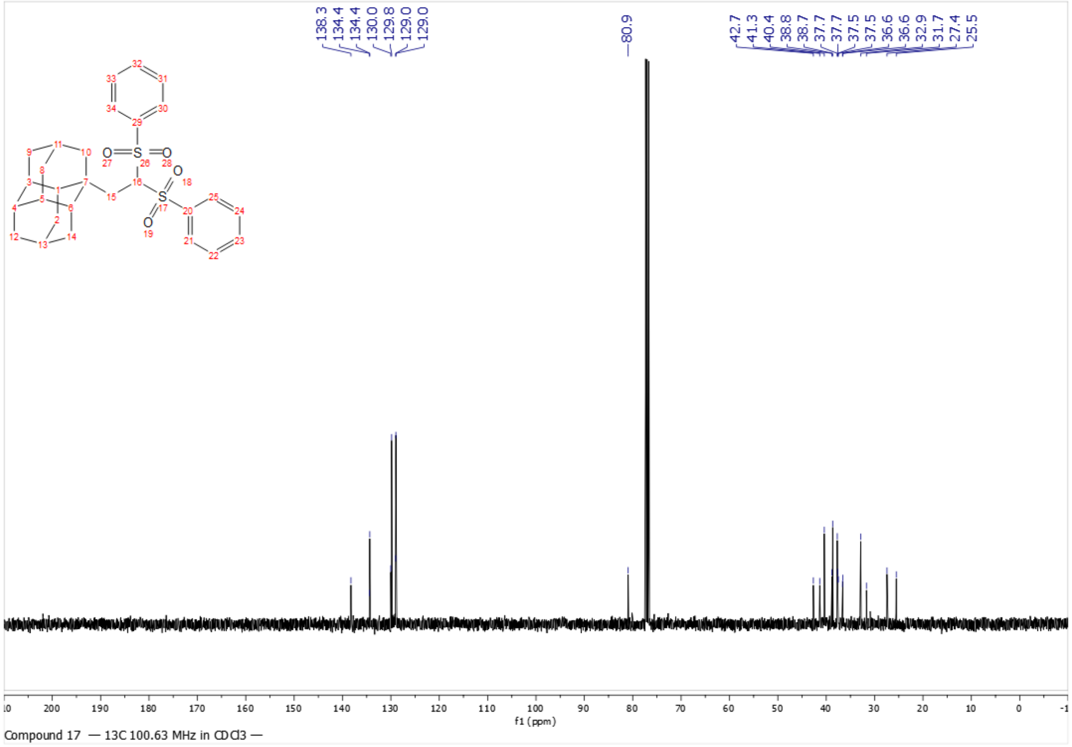

1-Azidodiamantane (**18**)

Medial azide (**18m**):

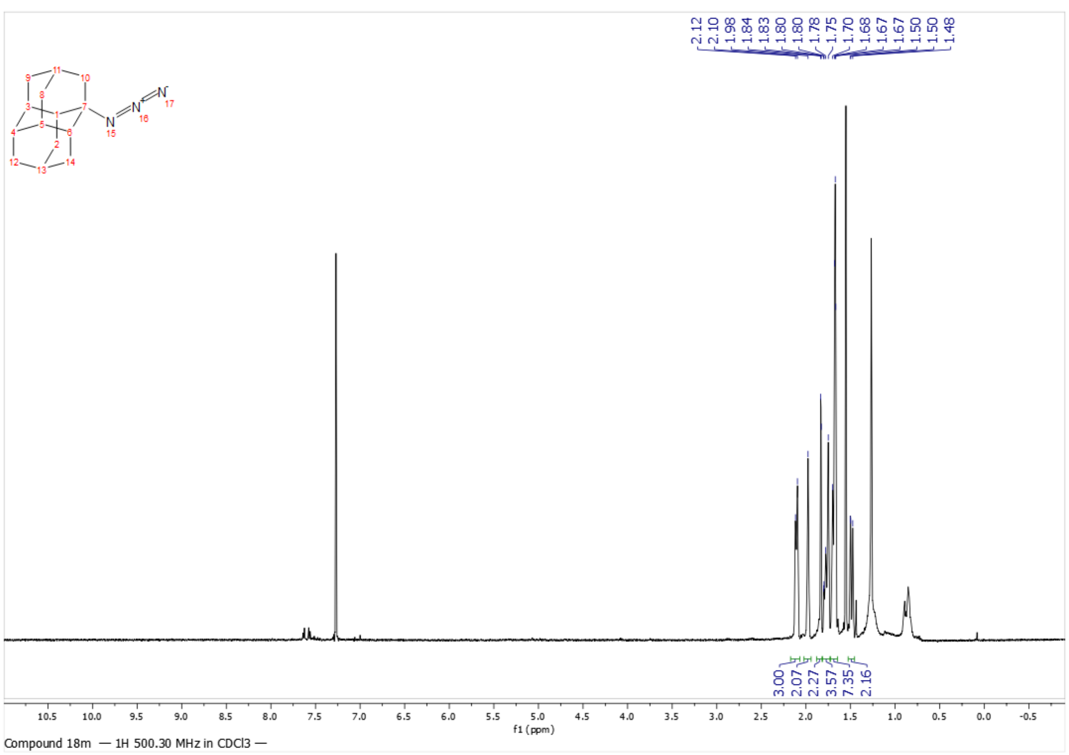

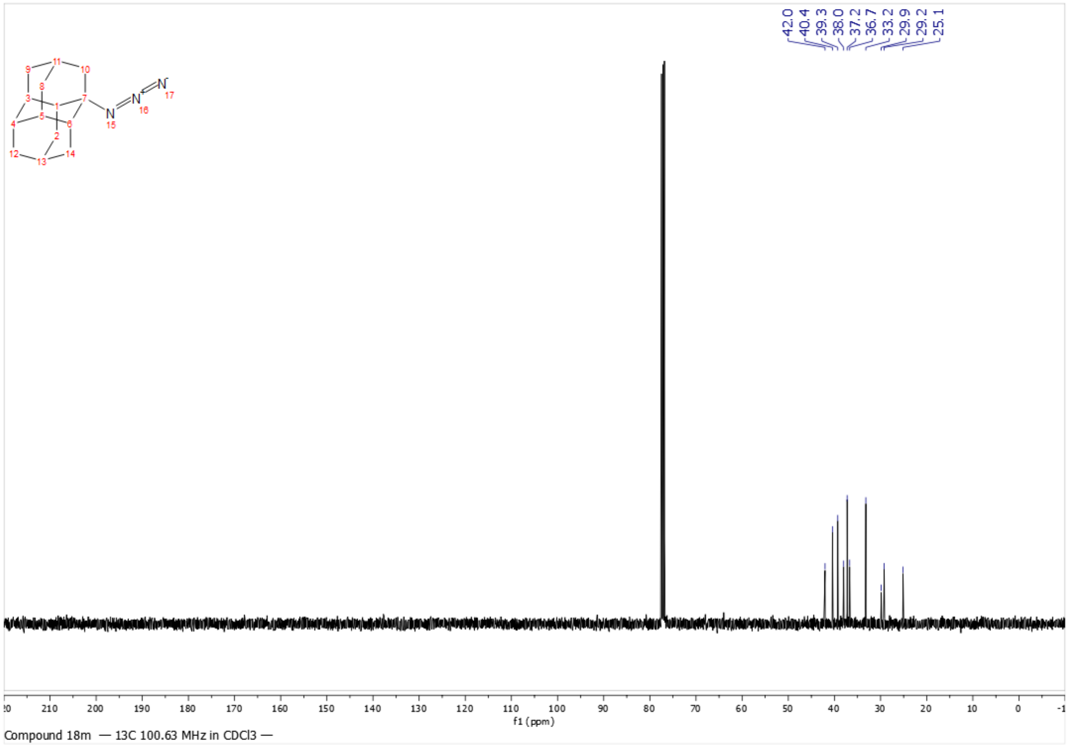

Apical azide (**18a**):

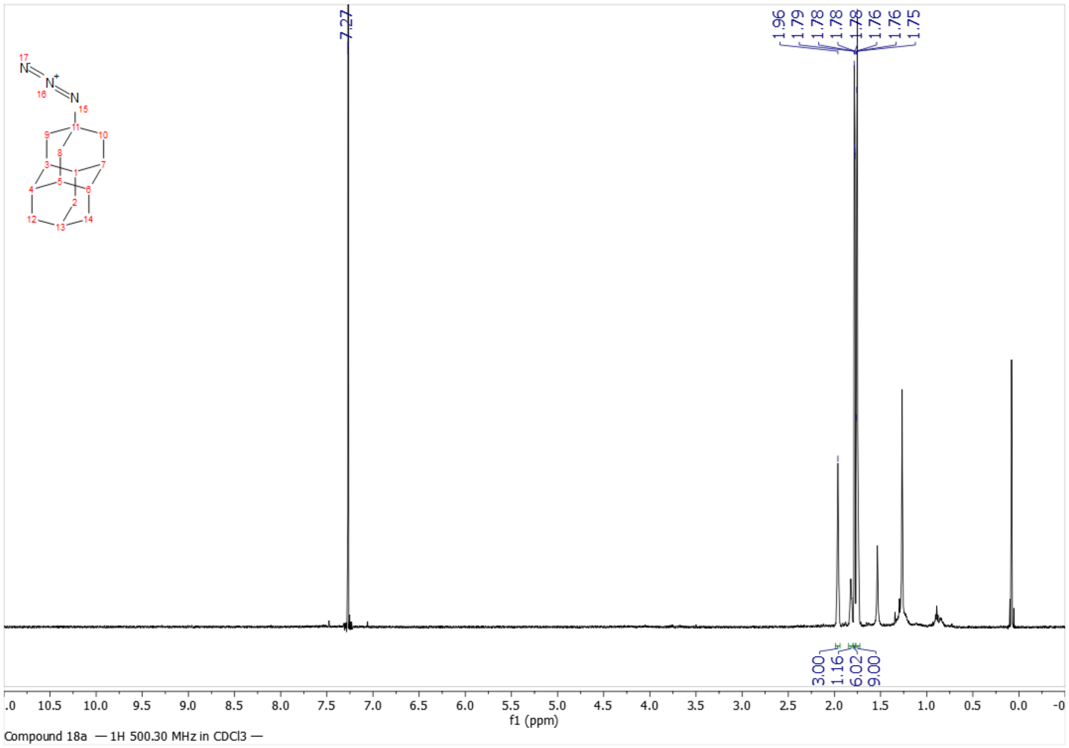

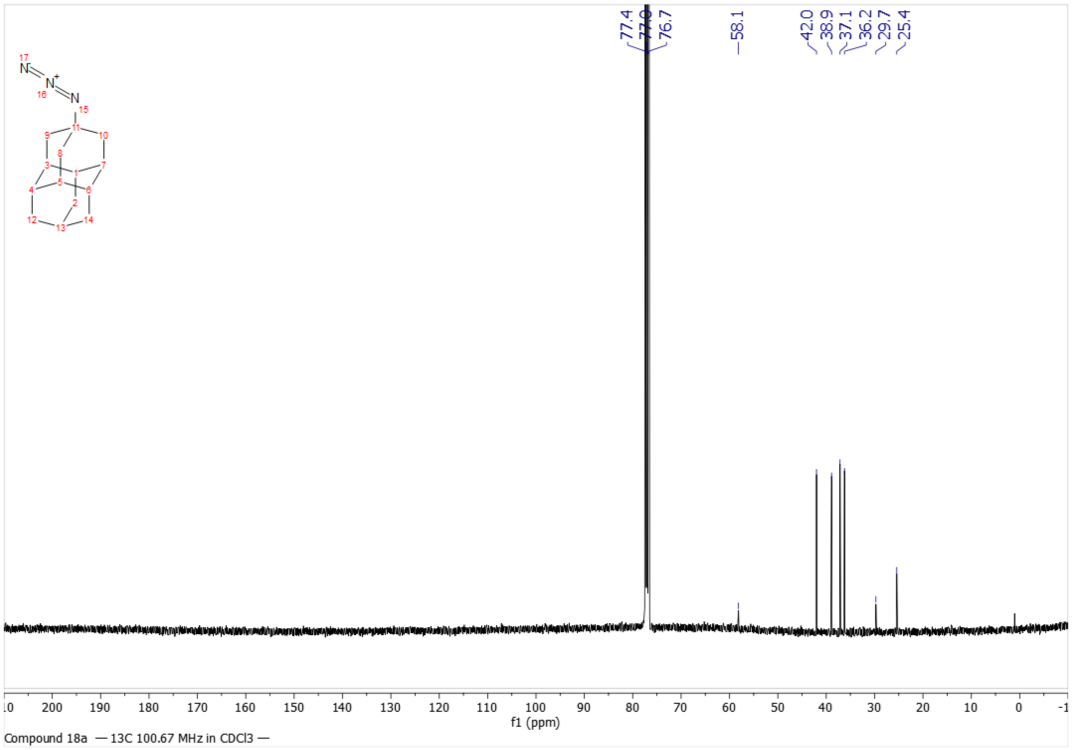

1-Diamantylcyanide (**19**)

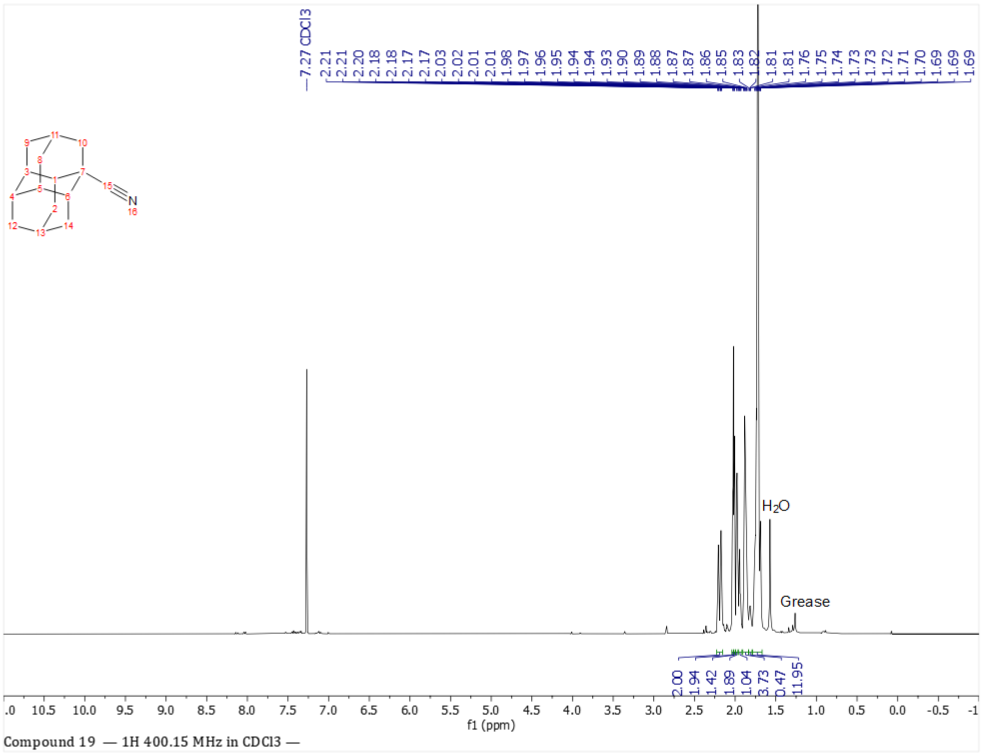

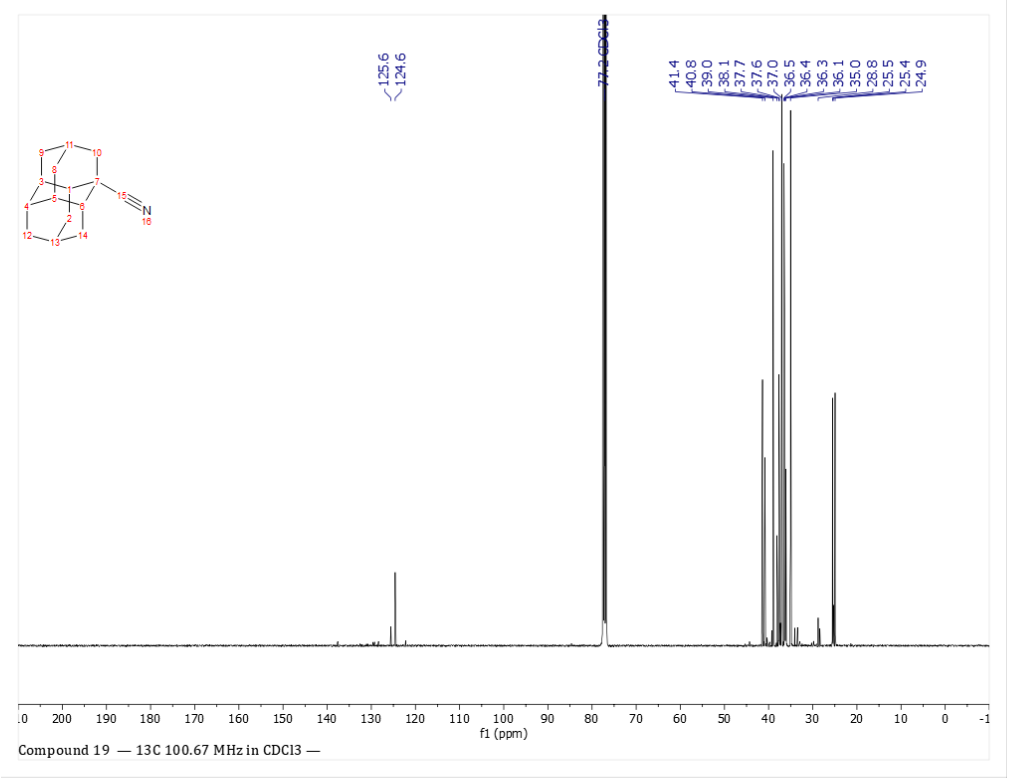

9-((Triamant-1-yl)(4-bromophenyl)methyl)malononitrile (**20**)

<u>Isomer A (apical):</u>

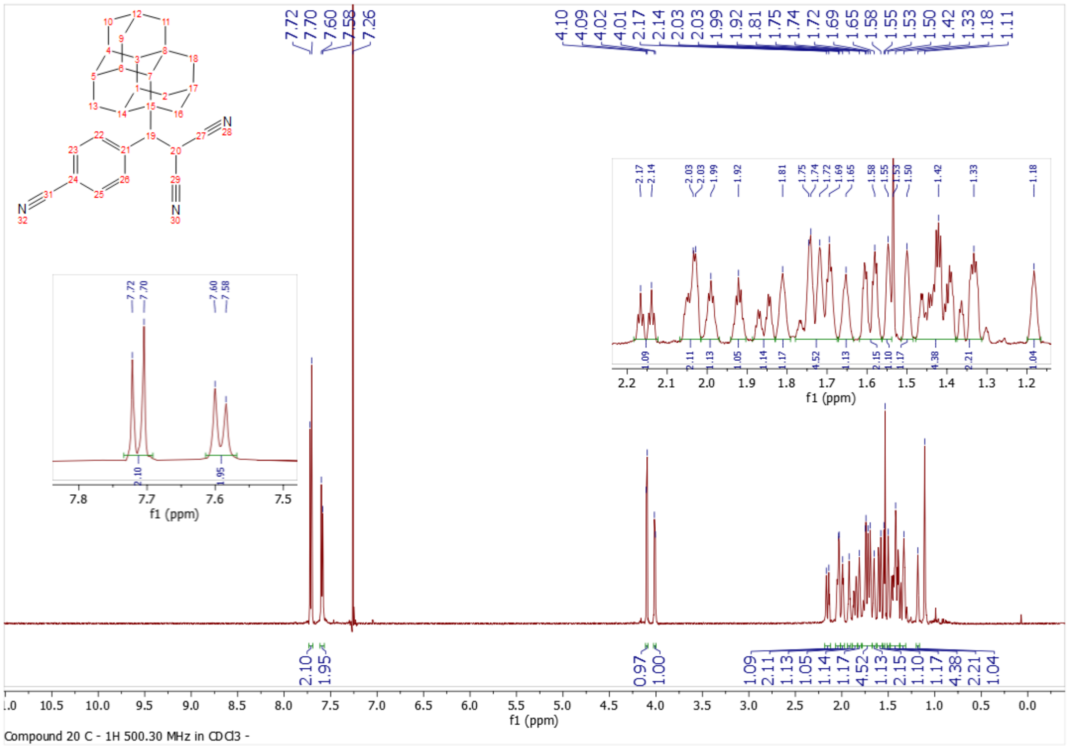

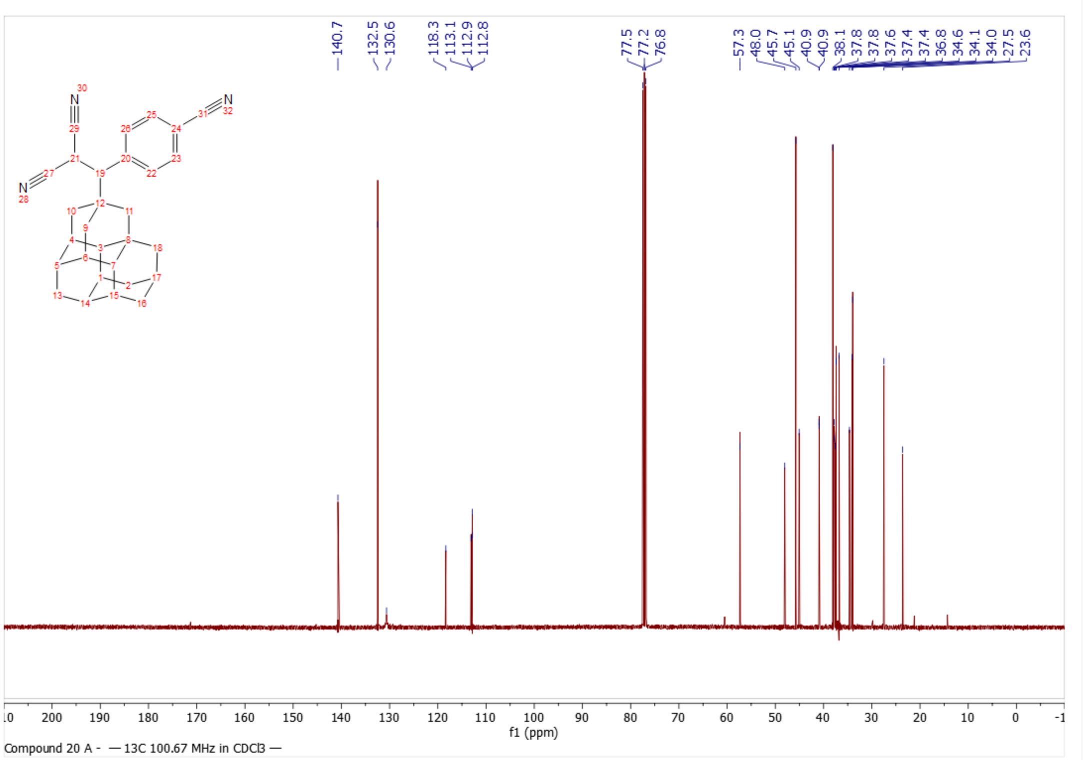

Isomer B:

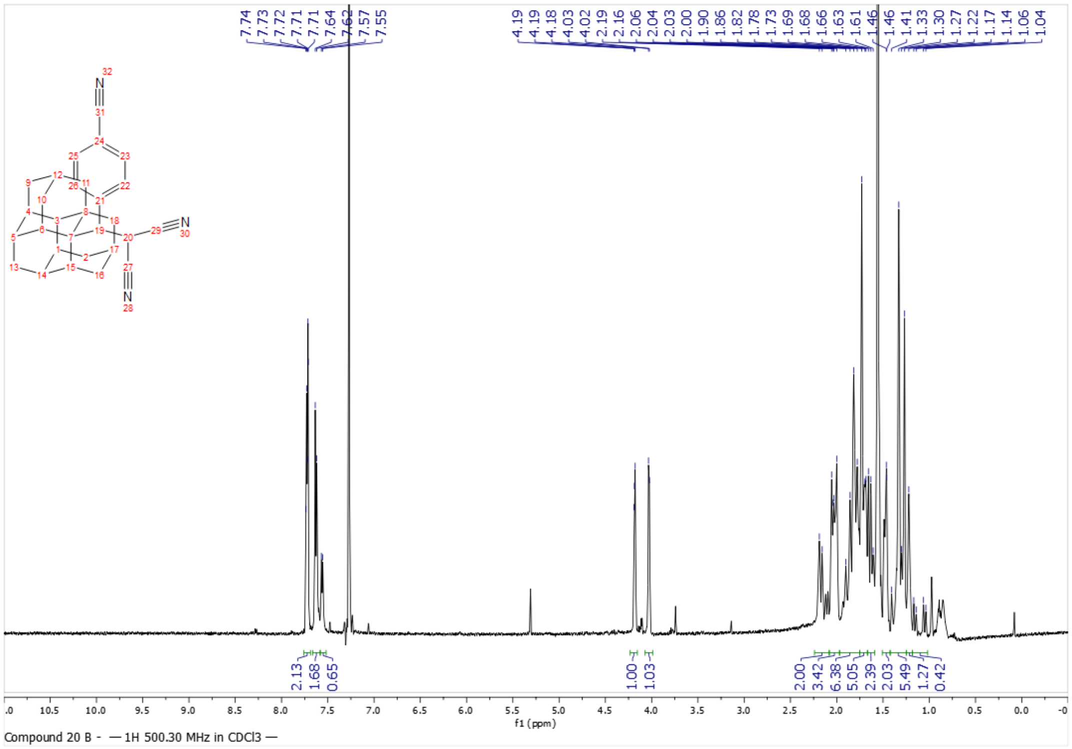

Isomer C:

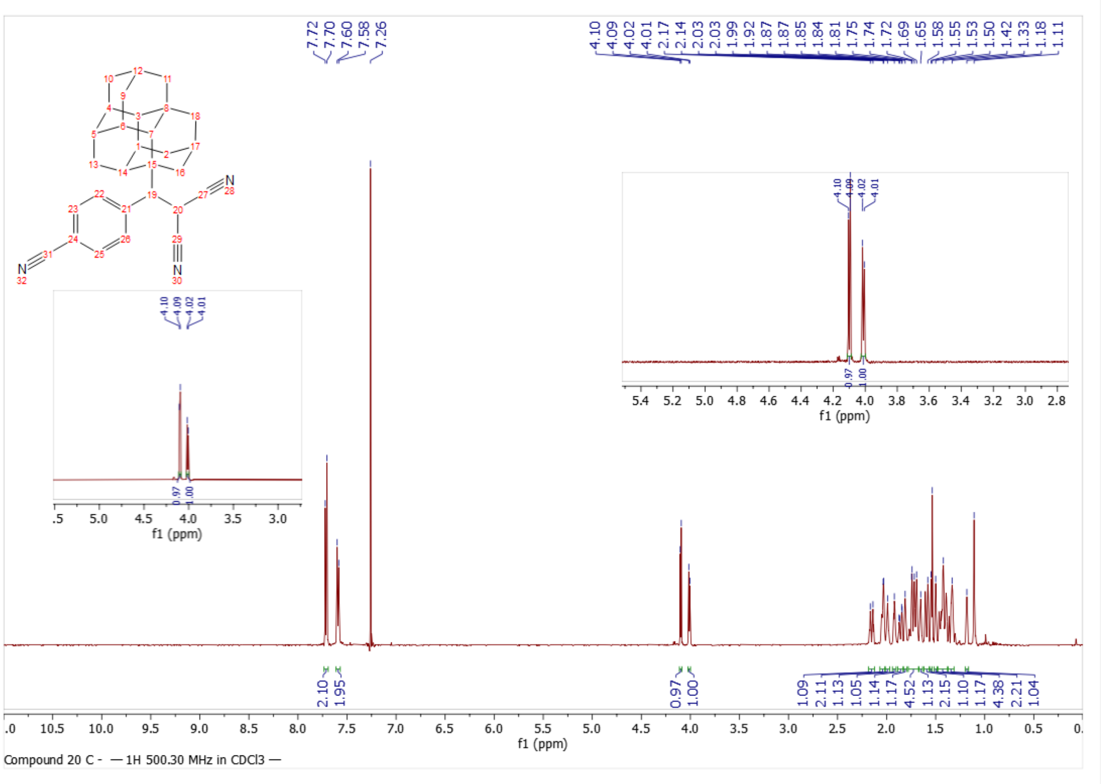
Compound 20 C - — 1H 500.30 MHz in CDCl3 —
f1 (ppm)

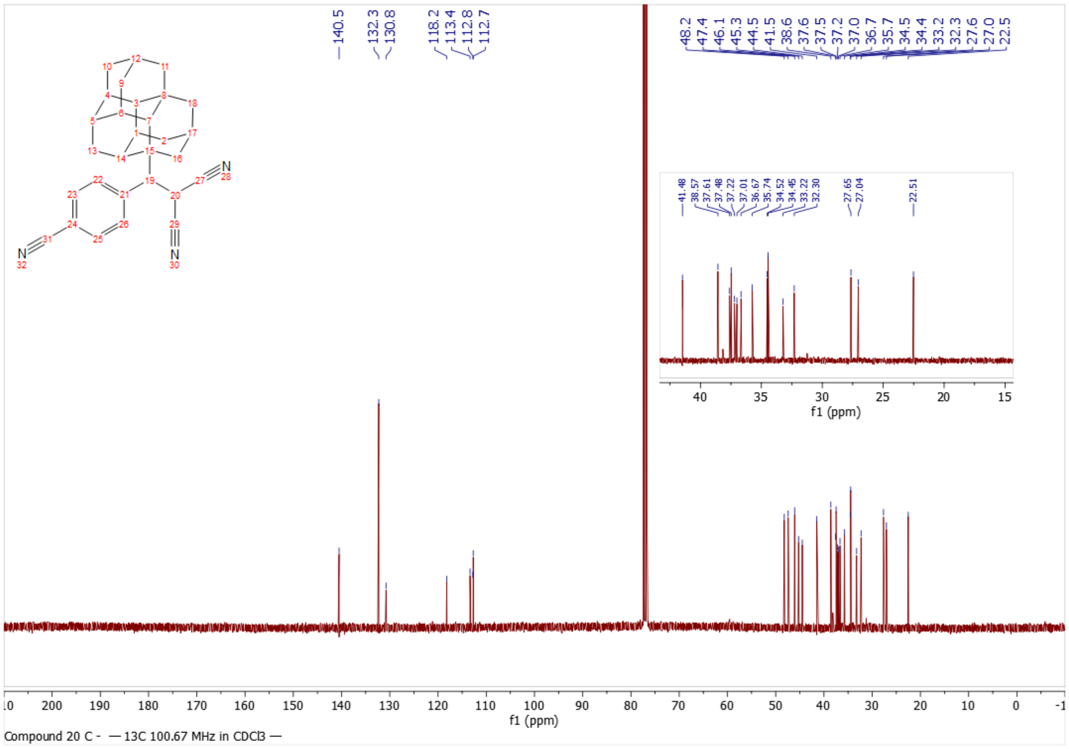
Compound 20 C - — 13C 100.67 MHz in CDCl3 —
f1 (ppm)

Isomer D:

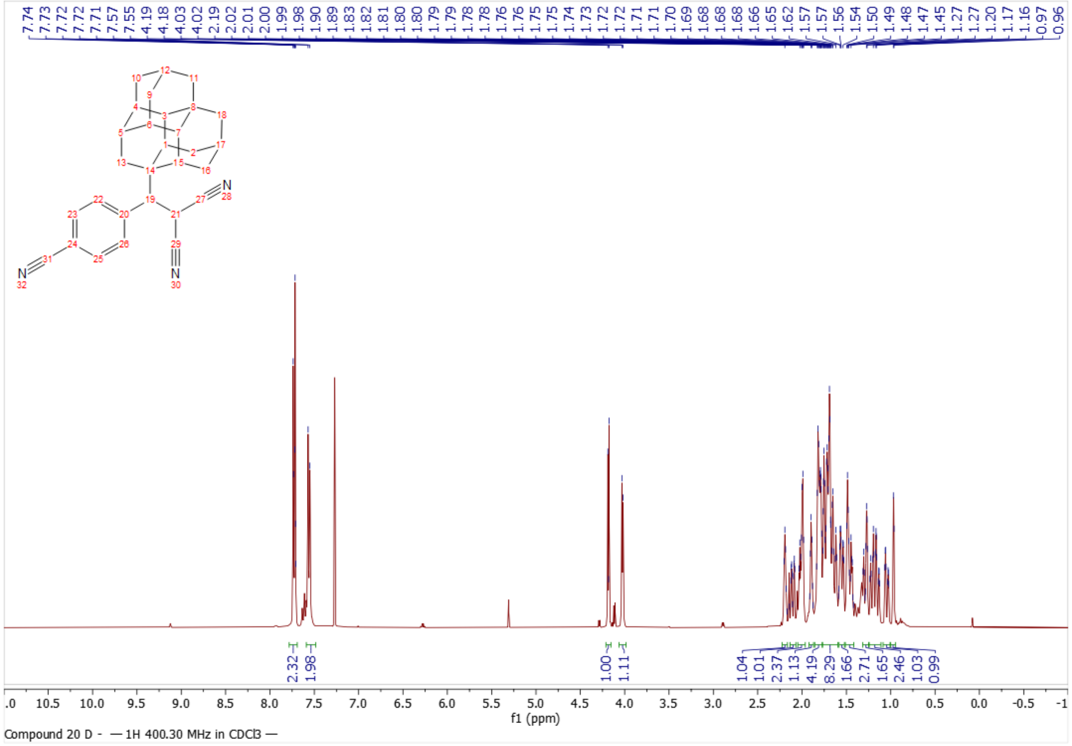
f1 (ppm)
Compound 20 D - — 1H 400.30 MHz in CDCl3 —

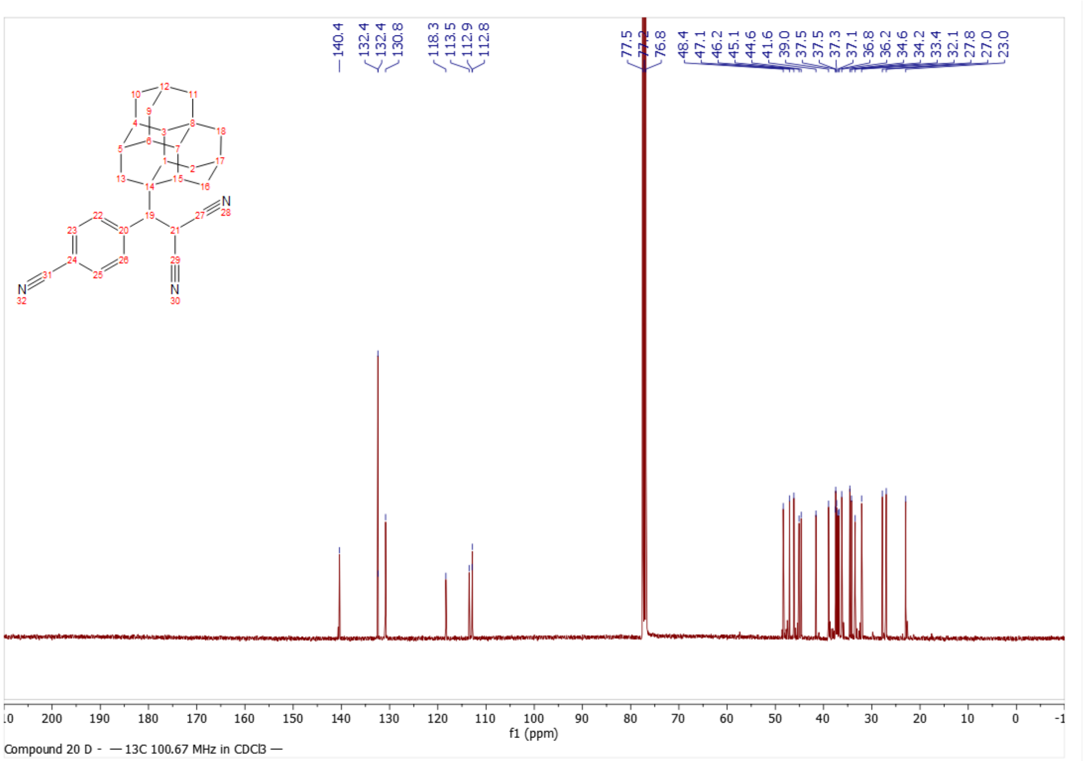
f1 (ppm)
Compound 20 D - — 13C 100.67 MHz in CDCl3 —

[121]-((tetramant-1-yl)(4-cyanophenyl)methyl)malononitrile (**21**)

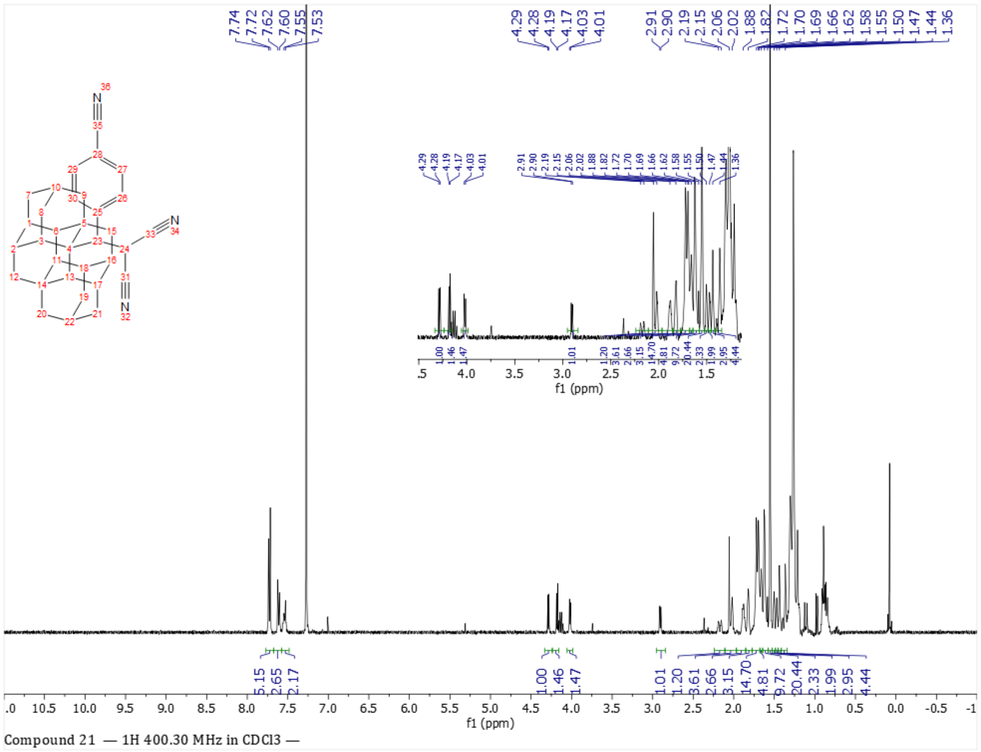

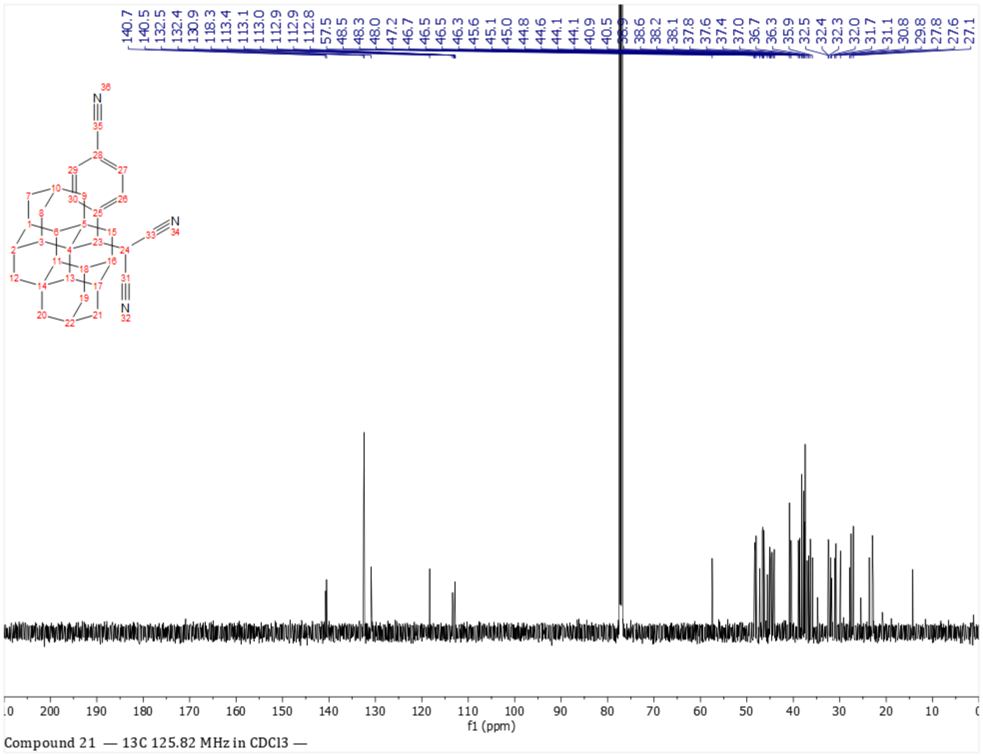

[1(2)3]-((tetramant-1-yl)(4-cyanophenyl)methyl)malononitrile (**22**)

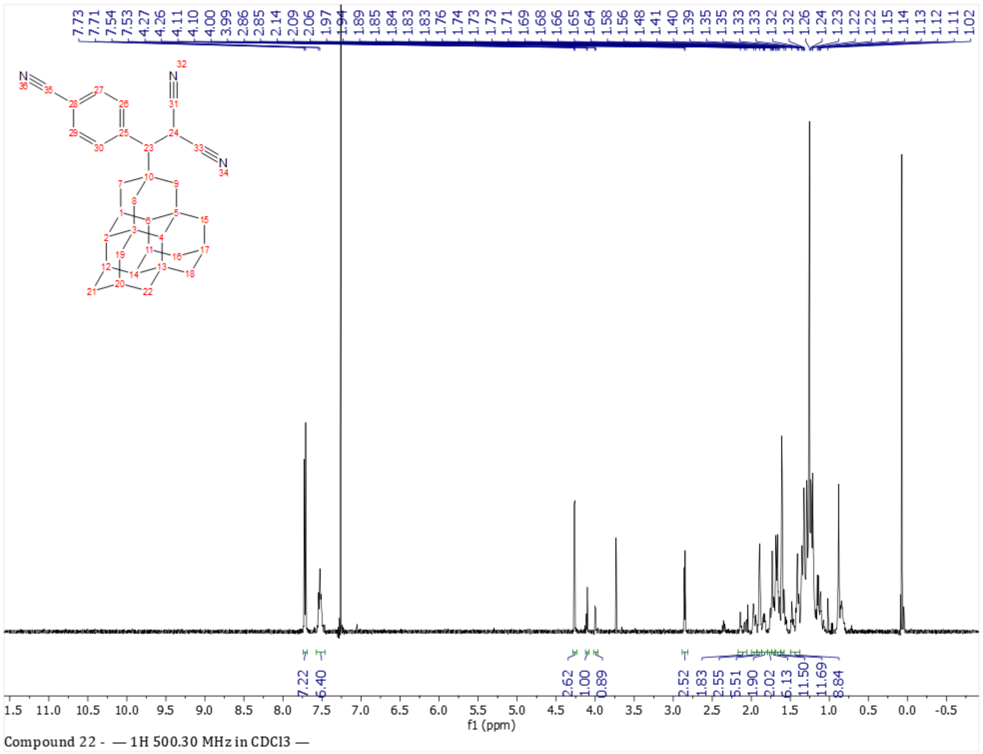

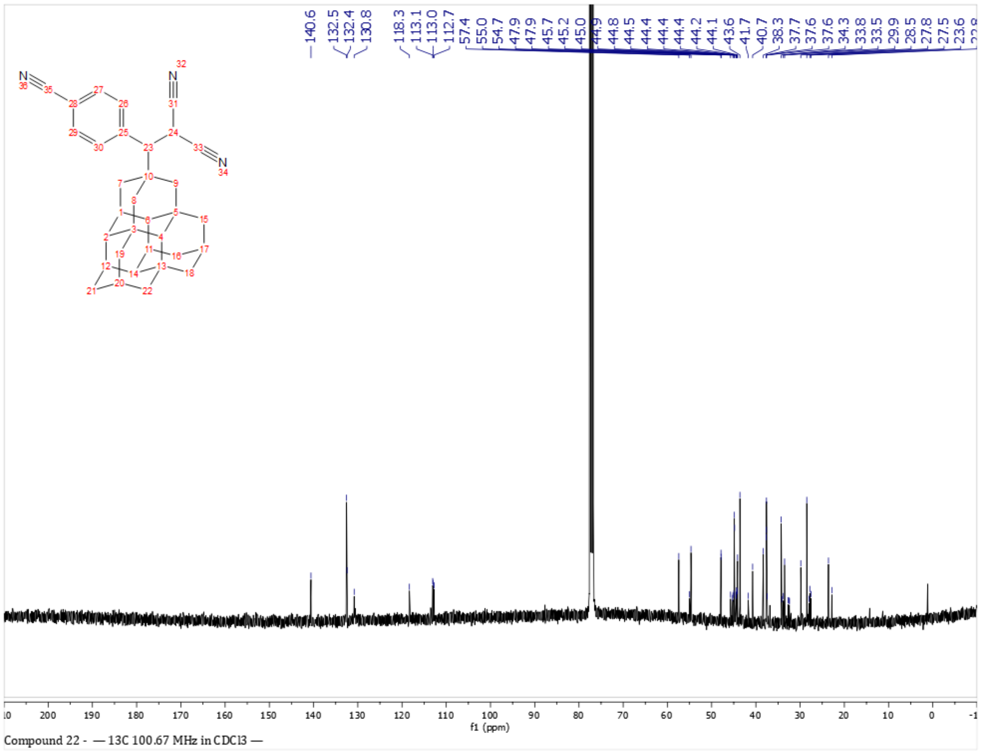

References

(1) Celis, F.; Campos-Vallette, M.; Cárcamo Vega, J.; Gómez-Jeria, J. S.; Aliaga, C. Raman and surface enhanced raman signals of the sensor 1-(4-Mercaptophenyl)-2,4,6-Triphenylpyridinium perchlorate. *J. Chil. Chem. Soc.* **2015**, *60*, 2944-2948.

(2) Sobhani, S.; Rezazadeh, S. Phosphomolybdic acid: An efficient and reusable catalyst for the synthesis of β-phosphono malonates. *J. Iran. Chem. Soc.* **2011**, *8*, 198-203.

(3) Boucher, M. M.; Furigay, M. H.; Quach, P. K.; Brindle, C. S. Liquid–Liquid Extraction Protocol for the Removal of Aldehydes and Highly Reactive Ketones from Mixtures. *Org. Proc. Res. Dev.* **2017**, *21*, 1394-1403.

(4) Zilate, B.; Fischer, C.; Schneider, L.; Sparr, C. Scalable Synthesis of Acridinium Catalysts for Photoredox Deuterations. *Synthesis* **2019**, 51, 4359-4365.

(5) Margrey, K. A.; Czaplyski, W. L.; Nicewicz, D. A.; Alexanian, E. J. A General Strategy for Aliphatic C–H Functionalization Enabled by Organic Photoredox Catalysis. *J. Am. Chem. Soc.* **2018**, *140*, 4213-4217.

(6) Berndt, J.-P.; Erb, F.; Ochmann, L.; Beppler, J.; Schreiner, P. Selective Phthalimido-*N*-oxyl (PINO)-Catalyzed C–H Cyanation of Adamantane Derivatives. *Synlett* **2019**, *30*, 493-498.

(7) Juneau, A.; Hope, T. O.; Malenfant, J.; Mesko, M.; Mcneill, J.; Frenette, M. Methods to Predict Potential Reagents in Iridium-Based Photoredox Catalysis Calibrated with Stern–Volmer Quenching Rate Constants. *ACS Catal.* **2022**, *12*, 2348-2356.

(8) Wang, Y.; Haze, O.; Dinnocenzo, J. P.; Farid, S.; Farid, R. S.; Gould, I. R. Bonded Exciplexes. A New Concept in Photochemical Reactions. Prediction of Dielectric Constants of Binary Solvents at Various Temperatures. *J. Org. Chem.* **2007**, *72*, 6970-6981.

(9) Jouyban, A.; Soltanpour, S. Prediction of Dielectric Constants of Binary Solvents at Various Temperatures. *J. Chem. Eng. Data* **2010**, *55*, 2951-2963.

(10) Buettner, G. R. Spin Trapping: ESR parameters of spin adducts 1474 1528V. *Free Rad. Bio. Med.* **1987**, *3*, 259-303.

(11) Zhou, R.; Liu, H.; Tao, H.; Yu, X.; Wu, J. Metal-free direct alkylation of unfunctionalized allylic/benzylic sp^3 C–H bonds via photoredox induced radical cation deprotonation. *Chem. Sci.* **2017**, *8*, 4654-4659.

(12) Elgrishi, N.; Rountree, K. J.; McCarthy, B. D.; Rountree, E. S.; Eisenhart, T. T.; Dempsey, J. L. A Practical Beginner's Guide to Cyclic Voltammetry. *J. Chem. Ed.* **2018**, *95*, 197-206.
